# Antiviral 4-Hydroxypleurogrisein and Antimicrobial Pleurotin Derivatives from Cultures of the Nematophagous Basidiomycete *Hohenbuehelia grisea*

**DOI:** 10.3390/molecules23102697

**Published:** 2018-10-19

**Authors:** Birthe Sandargo, Benjarong Thongbai, Dimas Praditya, Eike Steinmann, Marc Stadler, Frank Surup

**Affiliations:** 1 Department of Microbial Drugs, Helmholtz Centre for Infection Research GmbH, Inhoffenstraße 7, 38124 Braunschweig, Germany; birthe.sandargo@helmholtz-hzi.de (B.S.); benjarong.thongbai@helmholtz-hzi.de (B.T.); 2 German Centre for Infection Research (DZIF), partner site Hannover-Braunschweig, 38124 Braunschweig, Germany; 3 TWINCORE-Centre for Experimental and Clinical Infection Research (Institute of Experimental Virology) Hannover. Feodor-Lynen-Str. 7-9, 30625 Hannover, Germany; dimas.praditya@twincore.de (D.P.); eike.steinmann@rub.de (E.S.); 4 Research Center for Biotechnology, Indonesian Institute of Science, Jl. Raya Bogor KM 46, Cibinong 16911, Indonesia; 5 Department of Molecular and Medical Virology, Ruhr-University Bochum, 44801 Bochum, Germany

**Keywords:** Basidiomycota, fungi, HCV, *Hohenbuehelia grisea*, Pleurotin, secondary metabolites, structure elucidation

## Abstract

4-Hydroxypleurogrisein, a congener of the anticancer-lead compound pleurotin, as well as six further derivatives were isolated from the basidiomycete *Hohenbuehelia grisea*, strain MFLUCC 12-0451. The structures were elucidated utilizing high resolution electron spray ionization mass spectrometry (HRESIMS) and 1D and 2D nuclear magnetic resonance (NMR) spectral data and evaluated for their biological activities; for leucopleurotin, we provide Xray data. While most congeners showed moderate antimicrobial and cytotoxic activity, 4-hydroxypleurogrisein emerged as an inhibitor of hepatitis C virus infectivity in mammalian liver cells.

## 1. Introduction

**Figure 1:**
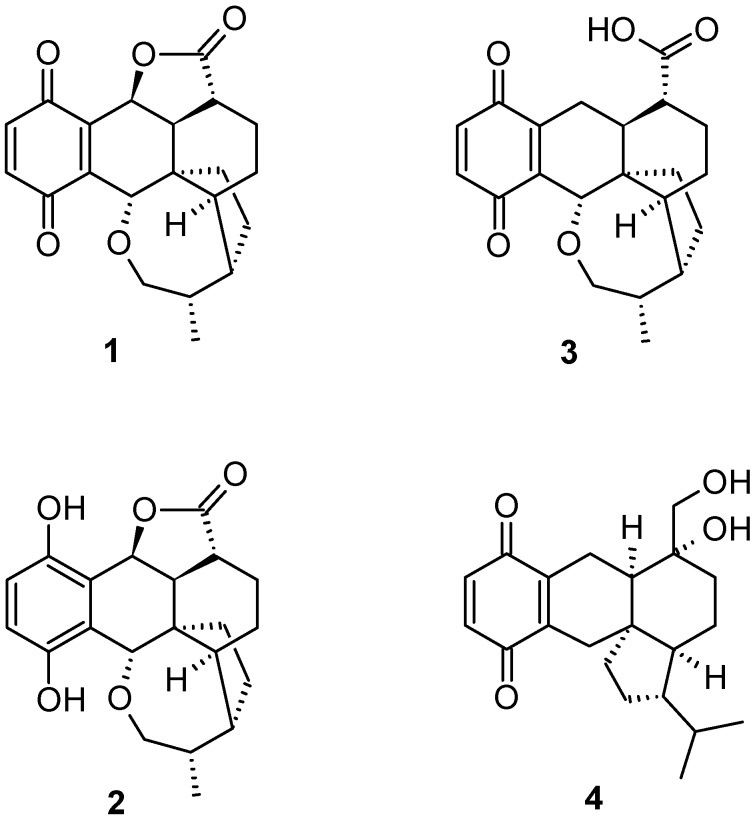
Chemical structures of previously isolated pleurotin derivatives: pleurotin (**1**), leucopleurotin (**2**), dihydropleurotinic acid (**3**) [5], and pleurogrisein (**4**) [13].

Fungi are known as talented producers of secondary metabolites [1]. With previously mainly ascomycetes studied for their potential to produce antibiotic agents, in recent years, basidiomycetes have become the center of attention in the search for new bioactive secondary metabolites [2] and the last group of antibiotics entering the market were the basidiomycete-derived pleuromutilins [3]. Another promising basidiomycete metabolite is the anti-cancer lead compound pleurotin (**1**), first isolated in 1947 from “*Pleurotus griseus* Peck” [4], which is now classified as *Hohenbuehelia grisea* (Peck) Singer. Pleurotin (**1**; Figure 1) and its derivatives leucopleurotin (**2**) and dihydropleurotinic acid (**3**) were shown to have activity against Gram-positive bacteria [4,5] and pathogenic fungi [6], as well as to exhibit anticancerogenic effects [5,7]. Moreover, the total synthesis of (±)-pleurotin (**1**) [8] and its production in multi-gram scale by fermentation [9] have already been accomplished. In recent years, pleurotin has become the center of attention as a potential new anti-cancer lead drug for its highly effective inhibition of the thioredoxin (Trx)–thioredoxin reductase (TrxR) system [10], a favorable target in the treatment of cancer as well as mercury intoxication [11].

**Figure 2:**
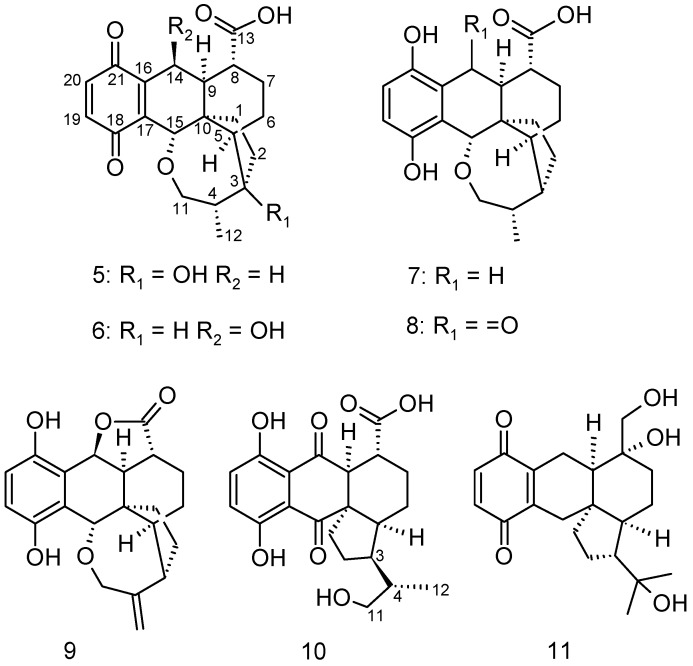
Chemical structures of newly isolated compounds **5**–**11**.

In a concurrent study, searching for promising new pleurotin derivatives from submerged cultures of *H. grisea* strain MFLUCC 12-0451, three cysteine-derived congeners of pleurotin, thiopleurotinic acids A and B, and pleurothiazole have been found, indicating a potential glutathione detoxification of basidiomycetes [12]. This discovery prompted us to investigate extracts of the strain more closely. Scale up of production and extensive, challenging chromatography procedures of the resulting crude extracts led to the isolation of seven pleurotin derivatives (Figure 2), of which three (**5**, **9**, and **11**) are entirely unprecedented. The other four metabolites (**6**–**8** and **10**) had been mentioned in the past as potential biosynthesis precursors of pleurotin during the course of pioneer studies on the biosynthesis of pleurotin in the group of Arigoni (ETH Zurich), summarized in Capaul (1992) [13]. However, the corresponding PhD theses contain no spectral data of these compounds, and they may vary in stereochemistry. The present paper is dedicated to the description of their isolation, structure elucidation and biological characterization.

## 2. Results and Discussion

As reported earlier, the producing strain MFLUCC 12-0451 was identified as *Hohenbuehelia grisea* (Peck) Singer [12]. *H. grisea*, more commonly known under its former name of the asexual morph *Nematoctonus robustus*, has been reported to produce pleurotin (**1**), dihydropleurotinic acid (**2**), and leucopleurotin (**3**) [5]. The group of Arigoni (ETH Zurich) has in the past extensively studied the biosynthesis of pleurotin and in the course of their research proposed several structures [13], which are also being reported with full NMR spectral data in this study for the first time.

**Figure 3:**
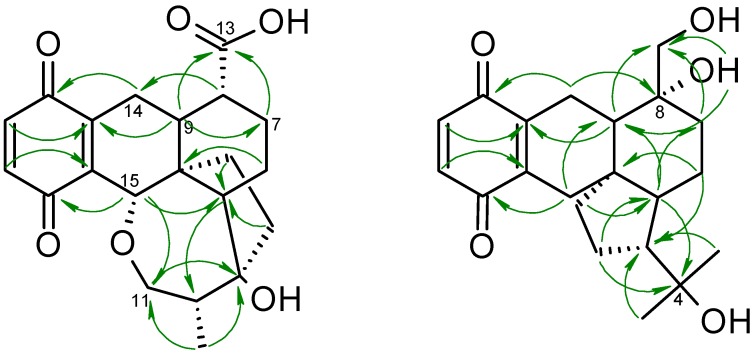
Selected ^1^H, ^13^C-HMBC correlations of 3-Hydroxy-dihydropleurotinic acid (**5**) and 4-Hydroxy-pleurogrisein (**11**).

3-Hydroxy-dihydropleurotinic acid (**5**) was isolated as an off-white to pale yellow powder with a molecular formula of C_21_H_24_O_6_, retrieved from a molecular ion cluster [M + H]^+^ at *m*/*z* 373.1661, indicating ten degrees of saturation. The correlation of ^1^H and ^13^C-NMR data (Table 1) in combination with HMBC correlations (Figure 3) led to the establishment of dihydropleurotinic acid as the underlying core structure [5,12], with the novelty of compound **5** being a hydroxyl functionality at C-3 (δ_C_ 82.8), which leads to the absolute configuration of 3*S*,4*S*,5*S*,8*R*,9*S*,10*R*,15*S*.

Highly similar to **5** is compound **6**, 14-hydroxy-dihydropleurotinic acid, obtained as a bright yellow solid with a molecular formula of C_21_H_24_O_6_ as well. The ^1^H and ^13^C-NMR data resemble largely those of compound **5**, with the major difference being two methines at δ_C/H_ 63.9/4.55 and 32.4/2.2. ^1^H,^1^H COSY and ^1^H, ^13^C-HMBC data confirmed the placement of these at positions C-14 and C-3, respectively. The chemical shift of C/H-14 also indicated the presence of a hydroxyl group (δ_H_ 4.16) and ROESY correlations of H-14 to H-9, as well as H-8 to H-15, and the absence of correlations between H-9 and H-15/H-8 indicate an *S*-configuration at C-14, and the absolute configuration is in line with the stereochemistry of leucopleurotin. A crystal structure with the absolute configuration of leucopleurotin has been attached (Figure S2), and is deposited at the Cambridge Crystallographic Data Center, CCDC 1872450. Compound **6** resembles likely the cleaved ester form of pleurotin (**1**).

The molecular formula C_21_H_26_O_5_ of leucopleurotinic acid (**7**), a white amorphous powder, was retrieved from HRESIMS, entailing nine degrees of saturation. Again, NMR data largely resembled those of dihydropleurotinic acid (**3**) with a major difference being upfield shifts in the ^13^C-NMR data of the olefinic carbons, suggesting a reduction of the quinone moiety to its corresponding hydroquinone form.

Closely related to **7** is metabolite **8**, 14-oxo-leucopleurotininc acid, acquired as an off-white amorphous powder and a molecular formula of C_21_H_24_O_6_. NMR spectral data coincide to great extent with compound **7**, yet indicate a keto group at position C-14 (δ_C_ 202.7).

A light brown amorphous powder with a molecular formula of C_21_H_22_O_5_ and eleven degrees of saturation is nematoctone (**9**). Its ^1^H and ^13^C-NMR data (Table 2) overlap in many parts with **8**, yet an ^1^H, ^13^C-HMBC correlation of C-13 to H-14 (δ_H_ 5.64) portend an ester bond between C-13 and C-14 (δ_C_ 74.9), generating leucopleurotin as the underlying core structure. However, the usual chemical shifts of C-4 and C-12 were not observed and instead carbon signals at δ_C_ 110 (C-12) and 147.7 (C-4) showed up. ^1^H, ^13^C-HMBC correlations (see Supplementary Materials) of C/H-12 to C/H-11 and C/H-3, as well as H-2, H-3, H-5, and H-11 to C-3 confirmed the position of a double bond between C-4 and C-12. The interproton distance measurements confirms an absolute configuration of 3*S*,5*S*,8*R*,9*S*,10*R*,14*S*,15*S* in line with leucopleurotin (Figure S2).

Di-oxo-leucopleurotinic acid (**10**) was isolated as a light brown powder. Its molecular formula was determined by HRMS as C_21_H_24_O_7_ and ten degrees of saturation. Compared to **8**, the molecular formula includes an additional oxygen atom. A closer look at the ^13^C spectra showed the appearance of a second keto group at δ_C_ 205.3 (C-15) in proximity to H-1 and H-9 (^1^H, ^13^C-HMBC; Supplementary Materials) and an upfield shift of C-11 to δ_C_ 67.4. This suggested that the seven-membered cyclic ether was cleaved, as compared to **9**.

Lastly, metabolite **11** (4-hydroxypleurogrisein) was isolated as an amber-colored solid, possessing the molecular formula of C_21_H_28_O_5_, calculated from the ion peak at *m*/*z* 383.1825 [M + Na]. ^13^C-NMR signals at δ_C_ 187.8 (C-21) and 188.1 (C-18) disclosed the quinone being present and showing ^1^H, ^13^C-HMBC correlations to two methylenes H-14 (δ_H_ 2.29, 3.03) and H-15 (δ_H_ 2.15, 2.61), respectively. A carbon at δ_C_ 74.8 (C-8) was observed instead of a methine, indicating a hydroxy function attached to C-8, and HMBC correlations of C-7 and C-9 to a methylene at δ_C_ 63.9 (C-13) suggested the proximity of another hydroxyl function. Another carbon at δ_C_ 72.8 (C-4), carrying an OH, showed correlations in the ^1^H, ^13^C-HMBC to two methyl groups at δ_H_ 1.18 (H-11) and 1.26 (H-12), leading to a 4-ring system as the underlying core structure, similar to **10**. This scaffold was confirmed by 1,1-ADEQUATE NMR data (measured in DMSO-*d*_6_, see Supplementary Materials). The stereochemistry of C-8 was assigned to be *R* by ROESY correlations of 8-OH to H-9, as well as a strong correlation of H-13 to H-15 (see Supplementary Materials), indicating an axial orientation of C/H_2_-13. The axial orientation of C/H_2_-13 was further confirmed by a *J*-HMBC NMR experiment, since a large coupling constant of 6.1 Hz was observed between H-7_ax_ (δ_H_ 1.23) and C-13 indicating an anti-periplanar orientation between H-7_ax_ and C-13 and thus confirming the 8*R* configuration, giving the absolute configuration of 4-hydroxypleurogrisein 3*S*,5*S*,8*R*,9*R*,10*R*.

### Biological Activities

To evaluate the biological activity of the isolated derivatives, compounds **5**–**11** were subjected to antimicrobial, cytotoxicity, and nematicidal activity assays. None of the isolated compounds, including **1**–**3**, displayed any signs of nematode toxicity in our assay, despite *H. grisea* being a nematode-trapping fungus and injecting a substance, the color of pleurotin, into captured nematodes in a water agar test (see Supplementary Materials, Figure S1). This is in accordance with previous results on pleurotin and other congeners (**1**–**3**), which also did not affect *C. elegans* [5]. Metabolites **5**–**11** have also been tested against a selection of microorganisms (Table 3). All, but **7**, showed signs of antimicrobial activity against yeasts, such as *Candida tenuis* (**5**: 100 µg/mL; **9**: 25 µg/mL) and *Rhodotorula glutinis* (**5**: 33.3 µg/mL), or Gram-positive bacteria such as *Bacillus subtilis* (**5** and **10**: 100 µg/mL, **8** and **11**: 50 µg/mL), *Micrococcus luteus* (**11**: 66.7 µg/mL), and *Staphylococcus aureus* (**11**: 33.3 µg/mL).

Assessing the cytotoxicity, only 4-hydroxypleurogrisein (**11**) displayed a comparable cytotoxicity to pleurotin, with IC_50_ values of 6.9 µg/mL for the murine fibroblast cell line L929 and IC_50_ 7.5 µg/mL for the cervix carcinoma cell line KB3.1 (Table 3).

**Figure 4:**
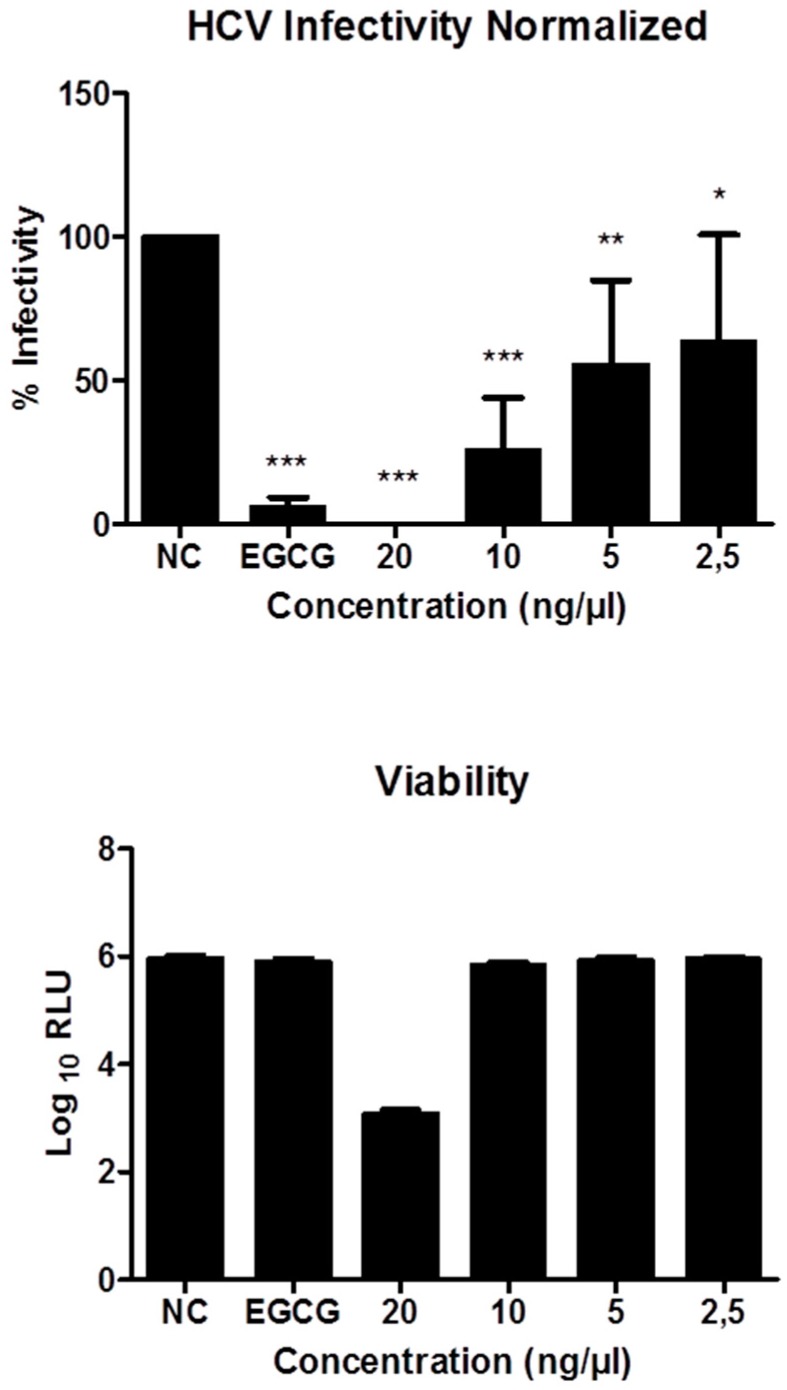
Antiviral activity of 4-hydroxypleurogrisin (**11**). NC-Negative control, EGCG-Positive control. HCV assay was performed in triplicates and is presented as the mean ± standard deviation. The asterisk indicates statistically significant differences (* *p* ≤ 0.05, ** *p* ≤ 0.01, *** *p* ≤ 0.001). Huh-7.5 cells were inoculated with RLuc-Jc1 reporter viruses in the presence of 4-hydroxypleurogrisein. The inoculum was removed 4 h later and monolayers were washed three times with PBS and overlaid with fresh medium containing no inhibitors. Infected cells were lysed three days later, and reporter virus infection was determined by renilla luciferase activity (**top**). The cell viability was measured by determination of firefly luciferase (**bottom**), which is stably expressed in the target cells.

Next, the compounds were tested for their inhibitory effect against hepatitis C virus (HCV). HCV infections is continuing to impose a global threat to human health with 71 million people infected worldwide. Although various potent direct acting antiviral agents have been licensed, high costs prevent the majority of infected individuals from having access to treatment. Out of the compounds tested, only 4-hydroxypleurogrisein (**11**) showed significant activities in vitro, while compound **5**, which was tested concurrently, was devoid of any activity on the host cells. As depicted in Figure 4, HCV infectivity decreased in a dose-dependent manner with an IC_50_ value of around 5 ng/μL and strong inhibitory effect at 10 ng/µL, while at 20 ng/µL cytotoxic effects were noted. The green tea molecule epigallocatechin gallate (EGCG) was used as positive control [14].

## 3. Materials and Methods

### 3.1. General

1D and 2D-NMR spectra were recorded on a Bruker Avance III 500 MHz spectrometer (Bremen, Germany) with a BBFO(plus) SmartProbe (^1^H 500 MHz, ^13^C 126 MHz), and a Bruker Avance III 700 MHz spectrometer (Bremen, Germany) with a 5 mm TCI cryoprobe (^1^ H 700 MHz, ^13^ C 175 MHz).

Chemical shifts were referenced to the solvents: chloroform-*d* (^1^H, δ = 7.27 ppm; ^13^C, δ = 77.0 ppm) and acetone-*d*_6_ (^1^H, δ = 2.05 ppm; 13C, δ = 29.3 ppm). HRMS mass spectra were measured on the Agilent 1200 series HPLC-UV system (Santa Clara, CA, USA) combined with ESI-TOF-MS (Maxis, Bruker, Bremen, Germany), scan range 100−2500 *m*/*z*, capillary voltage 4500 V, temperature 200 °C, (column 2.1 × 50 mm, 1.7 µm, C18 Acquity UPLC BEH [Waters, MZ-Analysetechnik, Mainz, Germany], solvent A: 95% 5 mM ammonium acetate buffer [pH 5.5, adjusted with 1 M acetic acid] with 5% acetonitrile; solvent B: 95% acetonitrile with 5% 5 mM ammonium acetate buffer); gradient: 10% solvent B increasing to 100% solvent B within 30 min, continuing at 100% B for further 10 min, *R*_F_ = 0.3 mL min^−1^, UV detection 200–600 nm. UV spectra were recorded using a Shimadzu UV-vis spectrophotometer UV-2450 (Shimadzu, Duisburg, Germany). Optical rotation was determined using a PerkinElmer 241 polarimeter (PerkinElmer LAS, Rodgau Jürgesheim, Germany).

### 3.2. Fungal Material

Basidiomes of the nematode-trapping fungus of *H. grisea* were collected from decaying wood in the tropical rainforest of Thailand near the Mushroom Research Centre, Chiang Mai Province, Thailand (http://www.mushroomresearchcentre.com/), in August of 2012 and the corresponding culture was obtained from basidiospores. The dried specimen and a corresponding culture are deposited at the mycological herbarium of the Mae Fah Luang University Culture Collection, Chiang Rai, Thailand, under the accession number MFLUCC 12-0451. Its 5.8S gene region, the internal transcribed spacer 1 and 2 (ITS) and part of the large subunit (LSU) were previously sequenced and published by Sandargo et al. [12] and the sequence data are deposited with GenBank, accession number MF150036.

### 3.3. Fermentation and Extraction

The strain *H. grisea* MFLUCC 12-0451 was cultivated in two different liquid media BAF (DSMZ 392), and Robbins medium [4] modified based on results of Shipley et al. [9] using alder extract (100 g *Alnus glutinosa* dead wood and branches without leaves, collection site Helmholtz Centre for Infection Research, campus Braunschweig, Germany, soaked for 24 h at room temperature in 1 L of deionized water)]. A sufficiently grown culture of *H. grisea* MFLUCC 12-0451 on BAF (DSMZ 392) agar was utilized to inoculate 200 mL of each medium in 500 mL Erlenmeyer flasks, incubated on a rotary shaker at 24 °C and 140 rpm. After 14 days, 30 mL of these seed cultures were shifted into 6 × 2 L Erlenmeyer flasks with 800 mL of the respective medium each. Incubation of the cultures at 24 °C and 140 rpm on rotary shakers occurred until all free glucose was consumed, after 21 days in BAF [12] and 24 days in modified Robbin’s media. Extraction followed an earlier published protocol, described in Sandargo et al. (2018) [12], leading to 4 g of crude extract for BAF medium and 2 g for modified Robbins medium. All crude extracts were filtered using an SPME Strata™-X 33 u Polymeric RP cartridge (Phenomenex, Inc., Aschaffenburg, Germany).

### 3.4. Isolation of Metabolites ***5**–**11***

The crude extracts of both media were pre-fractionated as described by Sandargo et al. (2018) [12] using RP. MPLC fractions were first subjected to analytical NP-HPLC using the Orbit 100 Diol column, 250 × 4 mm, 5 µm (MZ-Analysetechnik, Mainz, Germany), gradient: 100% solvent A (75% *n*-heptane and 25% tert.-butyl methyl ether) for 10 min, increasing over a period of 30 min to 100% solvent B (67% tert.-butyl methyl ether and 23% *n*-heptane with 10% acetonitrile), staying on 100% Solvent B for 10 min; flow rate: 2 mL/min and analytical RP-HPLC (maXis, Bruker) The isolation of compounds from MPLC pre-fractions was then performed using preparative NP-HPLC (Orbit 100 Diol column, 250 × 20 mm, 5 µm [MZ-Analysentechnik, Mainz, Germany]; solvent A: 75% *n*-heptane + 25% tert.-butyl methyl ether, solvent B: 67% tert.-butyl methyl ether + 23% *n*-heptane + 10% acetonitrile) or RP-HPLC (VP Nucleodur 100-5 C_18_ ec column, 250 × 20 mm, 5 µm [Macherey-Nagel, Düren, Germany], solvent A: water [MilliQ, Darmstadt, Germany], solvent B: acetonitrile). In both cases, applying a flow rate of 15 mL/min, UV detection at 215 and 248 nm, with an optimized gradient for each fraction minus 5–10% to plus 5–10% of the previously established eluting percentage, using the analytical NP-HPLC or RP-HPLC, over a period of 45 min. Table S1 lists the isolated compounds with their individual retention times using analytical RP-HPLC-MS (MaXis Bruker, Bremen, Germany) and the gradient used with the respective preparative HPLC. While compound 5 can only be found in modified Robbins media, all other metabolites appear in both media.

*3-Hydroxy-dihydropleurotinic acid* (**5**): off-white to pale yellow amorphous powder; [α${]}_{D}^{21}$ + 35° (*c* 1, ACN); UV (CHCl_3_) λ_max_ (log ε) 249 (5.0), 334 (3.7); IR: KBr, ῡ (cm^−1^) = 3400 s, 2950 s, 2900 m, 1700 s, 1600 vs, 1290 s, 1100 s; ^1^H- and ^13^C-NMR in CHLOROFORM-*d* see Table 1; HRESIMS *m*/*z* 373.1661 [M + H]^+^ (calcd. for C_21_H_25_O_6_^+^, 373.1651).

*14-Hydroxy-dihydropleurotinic acid* (**6**): yellow amorphous powder; [α${]}_{D}^{21}$ − 33° (*c* 1, ACN); UV (CHCl_3_) λ_max_ (log ε) 249 (4.9), 333 (3.6); ^1^H- and ^13^C-NMR in ACETONE-*d*_6_ see Table 1; HRESIMS *m*/*z* 373.1651 [M + H]^+^ (calcd. for C_21_H_25_O_6_^+^, 373.1651).

*Leucopleurotinic acid* (**7**): white amorphous powder; [α${]}_{D}^{21}$ + 16° (*c* 1, ACN); UV (CHCl_3_) λ_max_ (log ε) 233 (5.0), 297 (4.0); ^1^H- and ^13^C-NMR in ACETONE-*d*_6_ see Table 1; HRESIMS *m*/*z* 359.1868 [M + H]^+^ (calcd. for C_21_H_27_O_5_^+^, 359.1853).

*14-oxo-leucopleuotinic acid* (**8**): off-white amorphous powder; [α${]}_{D}^{21}$ + 19° (*c* 1, ACN); UV (CHCl_3_) λ_max_ (log ε) 233 (5.0), 271 (4.0), 378 (3.8); ^1^H- and ^13^C-NMR in CHLOROFORM-*d* see Table 1; HRESIMS *m*/*z* 373.1653 [M + H]^+^ (calcd. for C_21_H_25_O_6_^+^, 373.1651).

*Nematoctone* (**9**): light brown amorphous powder; [α${]}_{D}^{21}$ + 21° (*c* 1, ACN); UV (CHCl_3_) λ_max_ (log ε) 219 (5.1), 302 (4.1); IR: KBr, ῡ (cm^−1^) = 3400 vs, 2950 s, 2900 s, 2850 s, 1750 s, 1600 m, 1420 s, 1290 s, 1190 m, 1150 s, 1050 s, 800 s; ^1^H- and ^13^C-NMR in CHLOROFORM-*d* see Table 1; HRESIMS *m*/*z* 355.1539 [M + H]^+^ (calcd. for C_21_H_23_O_5_^+^, 355.1545).

*Di-oxo-leucopleurotinic acid* (**10**): light brown amorphous powder; [α${]}_{D}^{21}$ + 11° (*c* 1, ACN); UV (CHCl_3_) λ_max_ (log ε) 229 (5.0), 399 (3.5); ^1^H- and ^13^C-NMR in CHLOROFORM-*d* see Table 1; HRESIMS *m*/*z* 387.1447 [M − H]^−^ (calcd. for C_21_H_23_O_7_^−^, 387.1444).

*4-Hydroxypleurogrisein* (**11**): golden yellow amorphous powder; [α${]}_{D}^{21}$ + 24° (*c* 1, ACN); UV (CHCl_3_) λ_max_ (log ε) 249 (4.7) 348 (3.7); IR: KBr, ῡ (cm^−1^) = 3400 s, 2950 s, 2900 s, 1650 vs, 1310 s, 1200 s, 1070s, 850 m; ^1^H- and ^13^C-NMR in ACETONE-*d*_6_ see Table 1 and DMSO-*d*_6_ (see Supplementary Materials); HRESIMS *m*/*z* 383.1825 [M + Na], 743.3760 [2M + Na], 721.3952 [2M + H]^+^, 343.1908 [M − H_2_O + H]^+^, 325.1795 [M − 2 H_2_O + H]^+^, (calcd. for C_21_H_29_O_5_^+^, 361.2169).

### 3.5. Biological Activities

#### 3.5.1. Antimicrobial Activities

The minimum inhibitory concentration (MIC) for each compound was ascertained in a serial dilution assay in 96-well microtiter plates, as previously published by Kuhnert et al. [15], using YM media for yeasts and filamentous fungi and BD^TM^ Difco™ Mueller Hinton Broth for bacteria.

#### 3.5.2. Cytotoxicity Assay

In vitro cytotoxicity assay was performed as described by Richter et al. [16] against the mouse fibroblast cell line L929 and the cervix carcinoma cell line KB3.1.

#### 3.5.3. Nematicidal Activity Assay

Nematicidal activity of the isolated metabolites against *Caenorhabditis elegans*, grown on nematode agar (soy peptone 2 g, NaCl 1 g, agar 20 g, 1000 mL deionized water; adding 0.5 mL cholesterol (1 mg/mL EtOH), 1 mL 1 M CaCl_2_, 1 mL 1 M MgSO_4_, and 12.5 mL 40 mM potassium phosphate buffer after autoclaving; pH adjusted to 6.8) with living *E. coli* DSM498, at 20 °C for a week, was assessed according to a protocol by Kuephadungphan et al. [17].

#### 3.5.4. Inhibitory Effects on HCV Infectivity

This assay was carried out as described previously by Mulwa et al. [18]. Huh7.5 cells stably expressing Firefly luciferase (Huh7.5 Fluc) were cultured in Dulbecco’s modified minimum essential medium (DMEM, Life Technologies, Darmstadt, Germany) containing 2 mM L glutamine, 1 × minimum essential medium nonessential amino acids (MEM NEAA, Life Technologies), 100 μg/mL streptomycin, 100 IU/mL penicillin (Life Technologies), 5 μg/mL blasticidin and 10% fetal bovine serum. Cells were maintained in a 37 °C environment with 5% CO_2_ supply. Cells were infected with Jc1-derived Renilla reporter viruses in the presence or absence of compounds as described previously [19]. Infected cells were lysed and then frozen at −80 °C for 1 h following measurements of Renilla and Firefly luciferase activities on a Berthold Technologies Centro XS3 Microplate Luminometer (Bad Wildbad, Germany) as indicators of viral genome replication and cell viability, respectively.

## Figures and Tables

**Table 1:** ^1^H- and ^13^C-NMR spectroscopic data (^1^H 500 MHz, ^13^C 125 MHz, CDCl_3_) for **5**; (^1^H 700 MHz, ^13^C 176 MHz, CDCl_3_) for **8**, and (^1^H 500 MHz, ^13^C 125 MHz, acetone-*d*_6_) for **6**, (^1^H 700 MHz, ^13^C 176 MHz, acetone-*d*_6_) for **7**.

Pos	5		6		7		8	
	δ_C_, Type	δ_H_ (*J* in Hz)	δ_C_, Type	δ_H_ (*J* in Hz)	δ_C_, Type	δ_H_ (*J* in Hz)	δ_C_, Type	δ_H_ (*J* in Hz)
1	31.9, CH_2_	1.50, m	36.9, CH_2_	2.27, ddd (12.2, 9, 2.6)	34.4, CH_2_	1.35, m	33.6, CH_2_	1.29, m
		1.93, m				1.90, m		1.94, m
2	34.8, CH_2_	1.53, m	25.9, CH_2_	1.69, m	26.4, CH_2_	1.77, m	25.0, CH_2_	1.78, m
		2.30, m		1.75, m		1.90, m		1.93, m
3	82.8, C		46.0, CH	2.08, m	46.3, CH	2.31, m	43.9, CH	2.34, m
4	39.1, CH	2.14, m	32.4, CH	2.20, m	34.8, CH	2.14, m	33.7, CH	2.11, m
5	59.3, CH	1.94, m	52.6, CH	1.65, m	53.3, CH	1.86, m	50.6, CH	1.81, m
6	19.3, CH_2_	1.78, m	22.6, CH_2_	1.78, m 1.86,	23.2, CH_2_	1.85, m	21.7, CH_2_	1.87, m
		1.94, m		1.86, td (12.9, 3.9)		2.06, m		1.98, m
7	30.2, CH_2_	1.63, m	31.2, CH_2_	1.58, m	32.3, CH_2_	1.67, qd (12.8, 4.7)	29.5, CH_2_	1.87, m
		2.20, m		2.10, m		2.15, m		2.23, m
8	42.9, CH	2.13, m	43.3, CH	1.94, td (12.2, 4.0)	44.9, CH	2.35, m	42.4, CH	2.62, m
9	43.5, CH	2.05, dd (12, 3.2)	51.4, CH	2.06, m	46.2, CH	1.99, dd (12, 5.8)	58.9, CH	2.63, m
10	44.5, C		46.1, C		48.0, C		49.8, C	
11	75.9, CH_2_	3.69, dd (13.2, 3.6)	75.3, CH_2_	3.34, dd (12.3, 6.9)	77.6, CH_2_	3.91, dd (12.8, 8.0)	77.6, CH_2_	4.02, br d (12.8)
		4.02, dd (13.2, 8.3)		3.96, dd (12.3, 8.6)		4.18, dd (12.8, 2.0)		4.12, dd (12.8, 8.0)
12	16.6, CH_3_	1.09, d (6.9)	21.3, CH_3_	0.93, d (7.0)	21.8, CH_3_	1.07, d (7.3)	20.7, CH_3_	1.10, d (7.3)
13	179.6, C		176.8, C		177.5, C		176.8, C	
14	24.2, CH_2_	2.51, m	63.9, CH	4.55, br s	26.5, CH_2_	2.66, dd (17.5, 6.2)	202.7, C	
						2.74, br d (17.5)		
15	74.2, CH	4.43, s	73.2, CH	4.46, s	82.9, CH	5.0, s	82.2, CH	5.21, s
16	140.7, C		141.8, C		123.8, C		113.6, C	
17	139.8, C		139.9, C		121.6, C		121.6, C	
18	186.3, C		187.6, C		152.8, C		149.7, C	
19	137.7, CH	6.70 *, d (10.2)	138.6, CH	6.79, d (10.2)	114.9, CH	6.43, d (8.5)	128.0, CH	7.09, d (9.0)
20	135.6, CH	6.70 *, d (10.2)	136.8, CH	6.78, d (10.2)	115.8, CH	6.62, d (8.5)	119.0, CH	6.88, d (9.0)
21	186.8, C		187.8, C		148.8, C		157.4, C	
14-OH				4.16, br s				
18-OH						8.17, s		8.71

* Overlapping signal, C-19/20 may therefore be interchange.

**Table 2:** ^1^H- and ^13^C-NMR spectroscopic data (^1^H 700 MHz, ^13^C 176 MHz, CDCl_3_) for **9**–**10**, and (^1^H 500 MHz, ^13^C 125 MHz, acetone-*d*_6_) for **11**.

Pos	9		10		11	
	δ_C_, Type	δ_H_ (*J* in Hz)	δ_C_, Type	δ_H_ (*J* in Hz)	δ_C_, Type	δ_H_ (*J* in Hz)
1	32.0, CH_2_	2.10, m	36.9, CH_2_	1.89, m	39.4, CH_2_	1.11, m
		1.33, m		1.59, m		1.54, dd (11.6, 7.1)
2	30.0, CH_2_	1.9, m	28.5, CH_2_	1.41, m	27.22, CH_2_	1.73, m
		2.12, m		1.95, m		1.83, m
3	47.3, CH	3.04, m	41.6, CH	2.11, m	50.9, CH	2.31, m
4	147.7, C		37.0, CH	2.38, m	72.8, C	
5	52.5, CH	1.69, m	50.3, CH	1.90, m	54.55, CH	1.94, m
6	22.9, CH_2_	1.9, m	23.3, CH_2_	2.11, m	23.2, CH_2_	1.72, m
		1.56, m		1.90, m		2.05, m
7	24.0, CH_2_	1.47, dd (12.3, 3.9) 2.27, m	30.3, CH_2_	2.14, m	39.4, CH_2_	1.23, m
				1.74, m		2.29, m
8	39.3, CH	2.27, m	45.9, CH	2.54, td (12.1, 4.0)	74.8, C	
9	50.4, CH	2.44, d (7.1)	60.5, CH	2.81, d (12.1)	51.3, CH	1.8, m
10	48.9, C		59.4, C		43.8, C	
11	73.7, CH_2_	4.87, d (16.0)	67.4, CH_2_	3.64, dd (10.6, 3.3)	29.9, CH_3_	1.18, s
		4.50, d (16.0)		3.46, dd (10.6, 6.1)		
12	110.0, CH_2_	4.79, br s 4.88, br s	18.4, CH_3_	1.14, d (6.6)	31.5, CH_3_	1.26, s
13	174.3, C		174.9, C		63.9, CH_2_	3.12, dd (5, 1.7)
						3.40, dd (10.6, 5.0)
14	74.9, CH	5.64, d (7.3)	202.3, C		22.3, CH_2_	2.29, m
						3.03, m
15	80.0, CH	4.97, s	205.3, C		27.16 CH_2_	2.15, m
						2.61, m
16	118.4, C		112.2, C		142.6, C	
17	118.4, C		112.9, C		142.9, C	
18	152.2, C		155.8, C		188.1, C	
19	118.8, CH	6.87, d (8.8)	129.0, CH	7.24 *, d (0.6)	137.4, CH	6.73 *, s
20	119.5, CH	6.82, d (8.2)	128.5, CH	7.24 *, d (0.6)	137.2, CH	6.73 *, s
21	149.4, C		155.3, C		187.8, C	
4-OH						3.14, br s
8-OH						3.48, br s
13-OH						2.84, br s
18-OH		8.61, s		11.88, s		
21-OH				11.64, s		

* Overlapping signal, C-19/20 may therefore be interchanged.

**Table 3:** Minimum inhibitory concentration (MIC) in the serial dilution assay for bacteria and fungi and half-inhibitory concentrations (IC_50_ for cell lines) in µg/mL. For determination of MICs, 20 µL of either 1 mg/mL stock solution (67 µg/mL) or 1.5 mg/mL (100 µg/mL) of **1**–**3**, **5**–**11** were tested. Cell density was adjusted to 6.7 × 10^5^ cells/mL. Twenty microliters of Ethanol were used as negative control and displayed no activity against the selected test organisms. For IC_50_ values, 6 × 10^3^ cells/well were sown in 96-well microtiter plates and treated with **1**–**3**, **5**–**11** over five days.

Organism	MIC (µg/mL)										Reference (MIC)
	1 *	2 *	3 *	5	6	7	8	9	10	11	
*Mucor plumbeus* MUCL49355	100	-	100	-	-	-	-	-	-	-	Nystatin (12.5)
*Candida tenuis* MUCL29892	25	100	100	100	-	-	-	25	-	-	Nystatin (12.5)
*Bacillus subtilis* DSM10	50	25	100	100	-	-	50	-	100	50	Penicillin (6.3)
*Pichia anomala* DSM6766	66.7	66.7	66.7	-	-	-	-	-	-	-	Nystatin (16.7)
*Candida albicans* DSM1665	33.3	-	-	-	-	-	-	-	-	-	Nystatin (16.7)
*Rhodotorula glutinis* DSM10134	16.7	33.3	33.3	33.3	-	-	-	-	-	-	Nystatin (16.7)
*Mucor hiemalis* DSM2656	8.3	33.3	16.7	-	-	-	-	-	-	-	Nystatin (16.7)
*Micrococcus luteus* DSM1790	66.7	66.7	-	-	-	-	-	-	-	66.7	Oxytetracycline (0.4)
*Staphylococcus aureus* DSM346	33.3	33.3	66.7	-	-	-	-	-	-	33.3	Oxytetracycline (6.7)
Cell line	IC_50_ (µg/mL)										Reference (IC_50_)
L929 (IC_50_)	7.5	2.2	18	22	23	17	22	21	22	6.9	Epothilone B (0.00062)
KB3.1 (IC_50_)	8.5	2.8	18	22	22	20	22	18	22	7.5	Epothilone B (0.00003)

* Pleurotin (**1**), dihydropleurotinic acid (**2**), and leucopleurotin (**3**) used for comparison. - no inhibition observed under test conditions.

## Supplementary Materials

The following are available online. Table S1: Retention time of isolated peaks and gradients used for preparative HPLC, water agar test with *C. elegans*, Figure S1: Nematode captured by *H. grisea*, HRESIMS data of **5**–**11**, and full NMR data of **5**–**11**.
