# Supplementary material for: Antiviral 4-Hydroxypleurogrisein and Antimicrobial Pleurotin Derivatives from Cultures of the Nematophagous Basidiomycete Hohenbuehelia grisea
Source: Molecules. 2018 Oct 19;23(10):2697. doi: 10.3390/molecules23102697 (PMC6222660; doi:10.3390/molecules23102697)

Supplementary Material

Antiviral 4-hydroxypleurogrisein and antimicrobial pleurotin derivatives from cultures of the nematophagous basidiomycete *Hohenbuehelia grisea*

Birthe Sandargo [1], Benjarong Thongbai [1], Dimas Praditya [2,3], Eike Steinmann [2,4], Marc Stadler [1,*], and Frank Surup [1,*]

[1] Department of Microbial Drugs, Helmholtz Centre for Infection Research GmbH, Inhoffenstraße 7, 38124 Braunschweig, Germany and German Centre for Infection Research (DZIF), partner site Hannover-Braunschweig, 38124 Braunschweig, Germany; birthe.sandargo@helmholtz-hzi.de; benjarong.thongbai@helmholtz-hzi.de; marc.stadler@helmholtz-hzi.de.

[2] TWINCORE-Centre for Experimental and Clinical Infection Research (Institute of Experimental Virology) Hannover. Feodor-Lynen-Str. 7-9, 30625 Hannover, Germany; dimas.praditya@twincore.de

[3] Research Center for Biotechnology, Indonesian Institute of Science, Jl. Raya Bogor KM 46, Cibinong, Indonesia.

[4] Department of Molecular and Medical Virology, Ruhr-University Bochum, 44801 Bochum, Germany; eike.steinmann@rub.de.

* Correspondence:

Email**:** marc.stadler@helmholtz-hzi.de and frank.surup@helmholtz-hzi.de;

Tel.: +49-531-6181-4256.

Content

Isolation of metabolites 5 – 11 5

Table S1: Retention time of isolated peaks and gradients used for preparative HPLC. 5

Water agar test with *C. elegans* 6

Figure S1: Nematode captured by *H. grisea* 6

Results 6

Reference 6

Figure S2: Crystal structure of Leucopleurotin (2). 7

Crystallographic data of Leucopleurotin (2; CCDC 1872450): 7

HRESIMS data of 3-Hydroxy-dihydropleurotinic acid (5) 8

^{1}H NMR spectrum (500 MHz, Chloroform-*d*) of 3-Hydroxy-dihydropleurotinic acid (5). 9

^{13}C NMR spectrum (125 MHz, Chloroform-*d*) of 3-Hydroxy-dihydropleurotinic acid (5). 10

HSQC NMR spectrum (500 MHz, Chloroform-*d*) of 3-Hydroxy-dihydropleurotinic acid (5). 11

^{1}H, ^{13}C HMBC NMR spectrum (500 MHz, Chloroform-*d*) of 3-Hydroxy-dihydropleurotinic acid (5). . 11

^{1}H, ^{1}H COSY NMR spectrum (500 MHz, Chloroform-*d*) of 3-Hydroxy-dihydropleurotinic acid (5)..... 12

^{1}H, ^{1}H ROESY NMR spectrum (500 MHz, Chloroform-*d*) of 3-Hydroxy-dihydropleurotinic acid (5). . 13

Figure S3: IR spectrum (KBr) of 3-Hydroxy-dihydropleurotinic acid (5). 16

HRESIMS data of 14-Hydroxy-dihydropleurotinic acid (6) 17

^{1}H NMR spectrum (500 MHz, Acetone-*d*$_6$) of 14-Hydroxy-dihydropleurotinic acid (6). 18

^{13}C NMR spectrum (125 MHz, Acetone-*d*$_6$) of 14-Hydroxy-dihydropleurotinic acid (6). 19

HSQC NMR spectrum (500 MHz, Acetone-*d*$_6$) of 14-Hydroxy-dihydropleurotinic acid (6). 19

^{1}H, ^{1}H COSY NMR spectrum (500 MHz, Acetone-*d*$_6$) of 14-Hydroxy-dihydropleurotinic acid (6)........ 20

HMBC NMR spectrum (500 MHz, Acetone-*d*$_6$) of 14-Hydroxy-dihydropleurotinic acid (6). 22

^{1}H, ^{1}H ROESY NMR spectrum (500 MHz, Acetone-*d*$_6$) of 14-Hydroxy-dihydropleurotinic acid (6). 23

HRESIMS data of Leucopleurotinic acid (7). 24

^{1}H NMR spectrum (700 MHz, Acetone-*d*$_6$) of Leucopleurotinic acid (7). 25

^{13}C NMR spectrum (176 MHz, Acetone-*d*$_6$) of Leucopleurotinic acid (7). 26

HSQC NMR spectrum (700 MHz, Acetone-*d*$_6$) of Leucopleurotinic acid (7). 27

^{1}H, ^{1}H COSY NMR spectrum (700 MHz, Acetone-*d*$_6$) of Leucopleurotinic acid (7). 28

HMBC NMR spectrum (700 MHz, Acetone-*d*$_6$) of Leucopleurotinic acid (7). 29

^{1}H, ^{1}H ROESY NMR spectrum (700 MHz, Acetone-*d*$_6$) of Leucopleurotinic acid (7) 30

HRESIMS data of 14-Oxo-leucopleurotinic acid (8). 31

^{1}H NMR spectrum (700 MHz, Chloroform-*d*) of 14-Oxo-leucopleurotinic acid (8). 32

^{13}C NMR spectrum (176 MHz, Chloroform-*d*) of 14-Oxo-leucopleurotinic acid (8). 33

^{1}H, ^{1}H COSY NMR spectrum (700 MHz, Chloroform-*d*) of 14-Oxo-leucopleurotinic acid (8). 34

HSQC NMR spectrum (700 MHz, Chloroform-*d*) of 14-Oxo-leucopleurotinic acid (8). 35

HMBC NMR spectrum (700 MHz, Chloroform-*d*) of 14-Oxo-leucopleurotinic acid (8). 36

^{1}H, ^{1}H ROESY NMR spectrum (700 MHz, Chloroform-*d*) of 14-Oxo-leucopleurotinic acid (8). 37

HRESIMS data of Nematoctone (9). 38

^{1}H NMR spectrum (700 MHz, Chloroform-*d*) of Nematoctone (9). 39

^{13}C NMR spectrum (176 MHz, Chloroform-*d*) of Nematoctone (9). 40

^{1}H, ^{1}H COSY NMR spectrum (700 MHz, Chloroform-*d*) of Nematoctone (9). 41

HSQC NMR spectrum (700 MHz, Chloroform-*d*) of Nematoctone (9). 42

HMBC NMR spectrum (700 MHz, Chloroform-*d*) of Nematoctone (9). 43

^{1}H, ^{1}H ROESY NMR spectrum (700 MHz, Chloroform-*d*) of Nematoctone (9). 44

Figure S4: IR spectrum (KBr) of Nematoctone (9). 46

HRESIMS data of Di-oxo-leucopleurotinic acid (10). 47

^{1}H NMR spectrum (700 MHz, Chloroform-*d*) of Di-oxo-leucopleurotinic acid (10). 48

^{13}C NMR spectrum (176 MHz, Chloroform-*d*) of Di-oxo-leucopleurotinic acid (10). 48

^{1}H, ^{1}H COSY NMR spectrum (700 MHz, Chloroform-*d*) of Di-oxo-leucopleurotinic acid (10). 50

HSQC NMR spectrum (700 MHz, Chloroform-*d*) of Di-oxo-leucopleurotinic acid (10). 51

HMBC NMR spectrum (700 MHz, Chloroform-*d*) of Di-oxo-leucopleurotinic acid (10). 52

^{1}H, ^{1}H ROESY NMR spectrum (700 MHz, Chloroform-*d*) of Di-oxo-leucopleurotinic acid (10) 53

HRESIMS data of 4-Hydroxy-pleurogrisein (11). 54

^{1}H NMR (500 MHz, Acetone-d_6) of 4-Hydroxy-pleurogrisein (11) 55

^{13}C NMR (125 MHz, Acetone-d_6) of 4-Hydroxy-pleurogrisein (11). 56

^{1}H, ^{1}H COSY NMR (500 MHz, Acetone-d_6) of 4-Hydroxy-pleurogrisein (11). 57

HSQC NMR (500 MHz, Acetone-d_6) of 4-Hydroxy-pleurogrisein (11). 57

HMBC NMR (500 MHz, Acetone-d_6) of 4-Hydroxy-pleurogrisein (11). 58

^{1}H, ^{1}H ROESY NMR (500 MHz, Acetone-d_6) of 4-Hydroxy-pleurogrisein (11). 59

Figure S5: IR spectrum (KBr) of 4-Hydroxy-pleurogrisein (11). 62

Table S2: ^{1}H, ^{13}C NMR data (500 MHz, DMSO-d_6) of 4-Hydroxy-pleurogrisein (11). 63

^{1}H NMR (500 MHz, DMSO-d_6) of 4-Hydroxy-pleurogrisein (11). 64

^{13}C NMR (125 MHz, DMSO-d_6) of 4-Hydroxy-pleurogrisein (11). 65

^{1}H, ^{1}H COSY NMR (500 MHz, DMSO-d_6) of 4-Hydroxy-pleurogrisein (11). 66

HSQC NMR (500 MHz, DMSO-d_6) of 4-Hydroxy-pleurogrisein (11). 66

HMBC NMR (500 MHz, DMSO-d_6) of 4-Hydroxy-pleurogrisein (11). 67

1,1 ADEQUATE NMR (500 MHz, DMSO-d_6) of 4-Hydroxy-pleurogrisein (11). 68

^{1}H, ^{1}H ROESY NMR (500 MHz, DMSO-d_6) of 4-Hydroxy-pleurogrisein (11).. 70

Isolation of metabolites 5 – 11

The isolation of compounds from MPLC pre-fractions was performed using preparative NP-HPLC, indicated as **NP** in the table, [Orbit 100 Diol column, 250 × 20 mm, 5 µm (MZ-Analysentechnik, Mainz, Germany); solvent A: 75% n-heptane + 25% tert.-butyl methyl ether, solvent B: 67% tert.-butyl methyl ether + 23% n-heptane + 10% acetonitrile] or RP-HPLC (indicated as **RP**) [VP Nucleodur 100-5 C_{18} ec column, 250 x 20 mm, 5µm (Macherey-Nagel, Düren, Germany), solvent A: water (MilliQ), solvent B: acetonitrile]. In both cases applying a flow rate of 15 mL/min, UV detection at 215 and 248 nm, with an optimized gradient for each fraction minus 5-10% to plus 5-10% of the previously established eluting gradient/percentage using analytical NP-HPLC or RP-HPLC over a period of 45 minutes.

Table S1: Retention time of isolated peaks and gradients used for preparative HPLC.

Compound	Elution time analytical RP-HPLC (maXis, Bruker)	Gradient for preparative HPLC and % of solvent B at elution time	Obtained amount [mg]
**5**	2.7 min	RP: 20 – 50% B, eluted at 40% B	94.4
**6**	7.5 min	RP: 30 - 60% B, eluted at 57% B	45
**7**	14 min	NP: 55 – 70% B, eluted at 60% B	16
**8**	14 min	RP: 30 – 55% B, eluted at 50% B	0.8
**9**	9.1 min	RP: 35 – 55% B, eluted at 47% B	0.6
**10**	7.5 min	RP 30 – 60% B, eluted at 55% B	5
**11**	8.5 min	NP: 40 – 70% B, eluted at 56% B	28

Water agar test with *C. elegans*

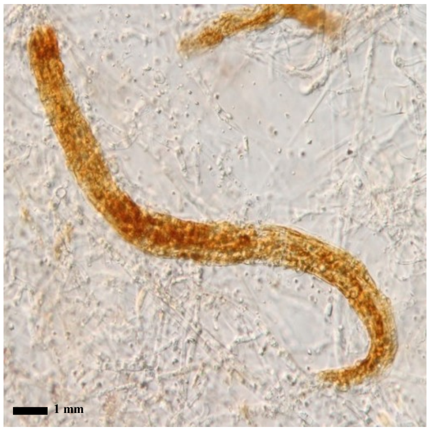

Figure S1: Nematode captured by *H. grisea*

To examine the general nematicidal activity of *H. grisea* MFLUCC12-0451, a water agar test was performed as described by Stadler (1993). For this purpose, a piece of a solid plate culture of the fungus was placed on one site of a water agar petri dish. On the opposite site *C. elegans* was placed and the petri dish inoculated for two to three weeks. After one week, additional nematodes were placed on the petri dish. The petri dishes were regularly checked for any salience using a stereomicroscope (Stemi 200-c, Zeiss, Germany).

Results

After three weeks in co-cultivation with nematodes, the fungus captured the nematodes by means of adhesive knobs. After another week, a noticeable strong amber staining of the nematodes occurred (Figure S1).

Reference

M. Stadler (1993) PhD thesis, TU Kaiserslautern.

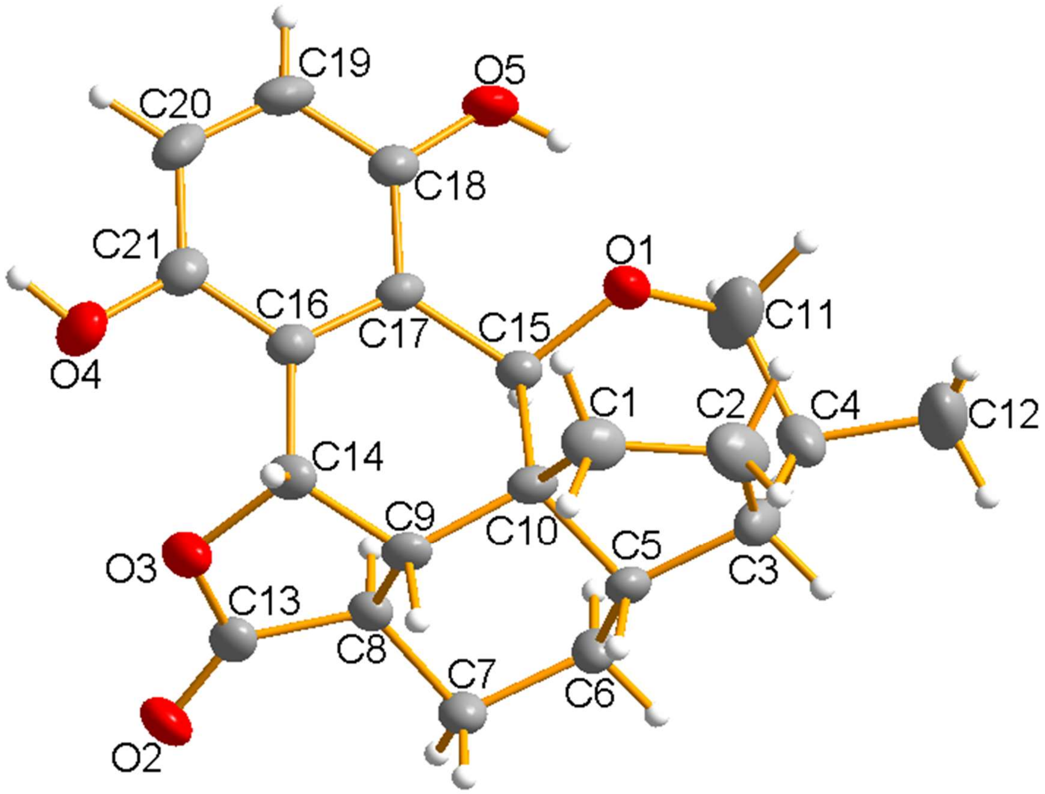

Figure S2: Crystal structure of Leucopleurotin (**2**).

Crystallographic data of Leucopleurotin (2; CCDC 1872450):

$C_{21}H_{24}O_5$, M = 356.40 g/mol, monoclinic, a = 8.6791(3) Å, b = 9.8923(4) Å, c = 10.5032(4), V = 897.19(6) Å^3, space group P2_1, Z = 2, Dcalc = 1.319 Mg/m^3, 13144 reflections measured in the range 1.95° ≤ θ ≤ 27.19°, 3722 independent reflections, R(int) = 0.0200, 320 parameters, 1 restraints. The final refinement resulted in R1 = 0.0445, wR2 = 0.0929 (I > 2σ(I)). The goodness of fit on F2 was 1.104.

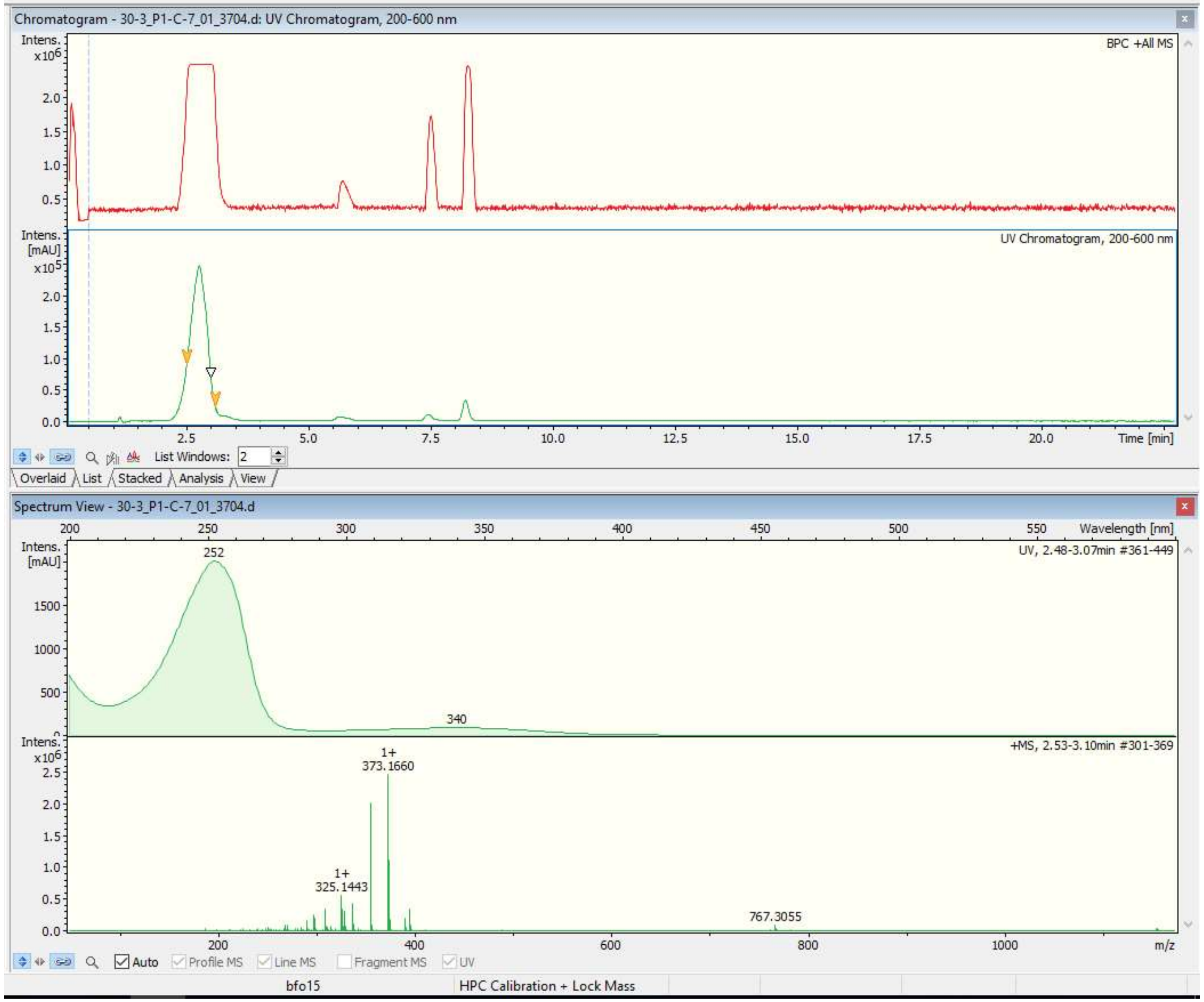

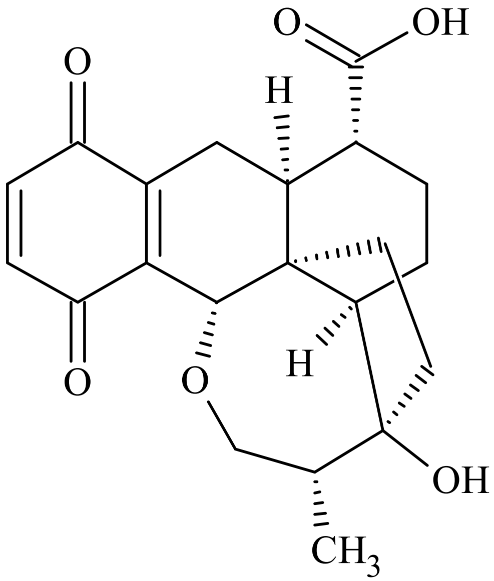

HRESIMS data of 3-Hydroxy-dihydropleurotinic acid (5)

^{1}H NMR spectrum (500 MHz, Chloroform-*d*) of 3-Hydroxy-dihydropleurotinic acid (5).

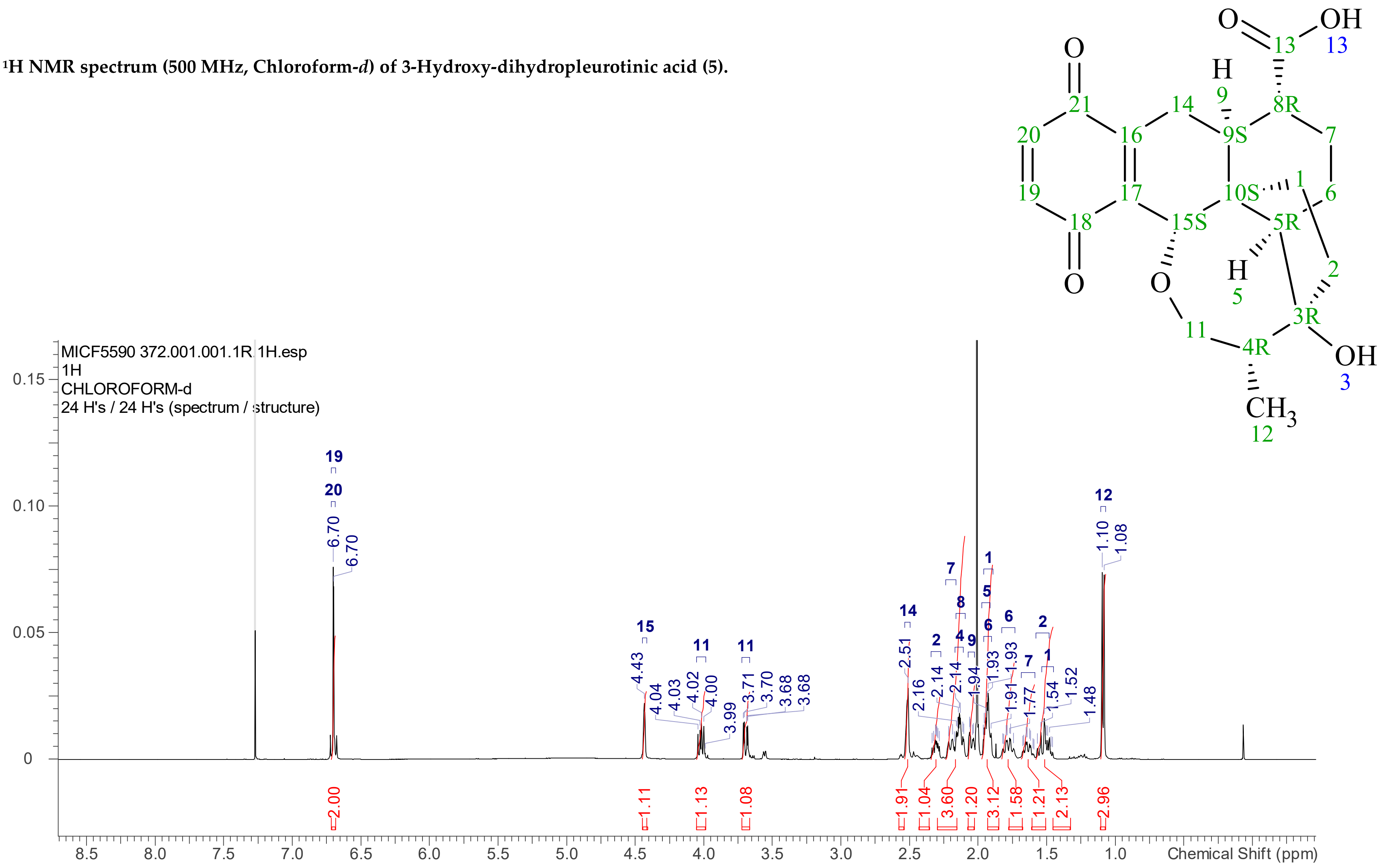

^{13}C NMR spectrum (125 MHz, Chloroform-*d*) of 3-Hydroxy-dihydropleurotinic acid (5).

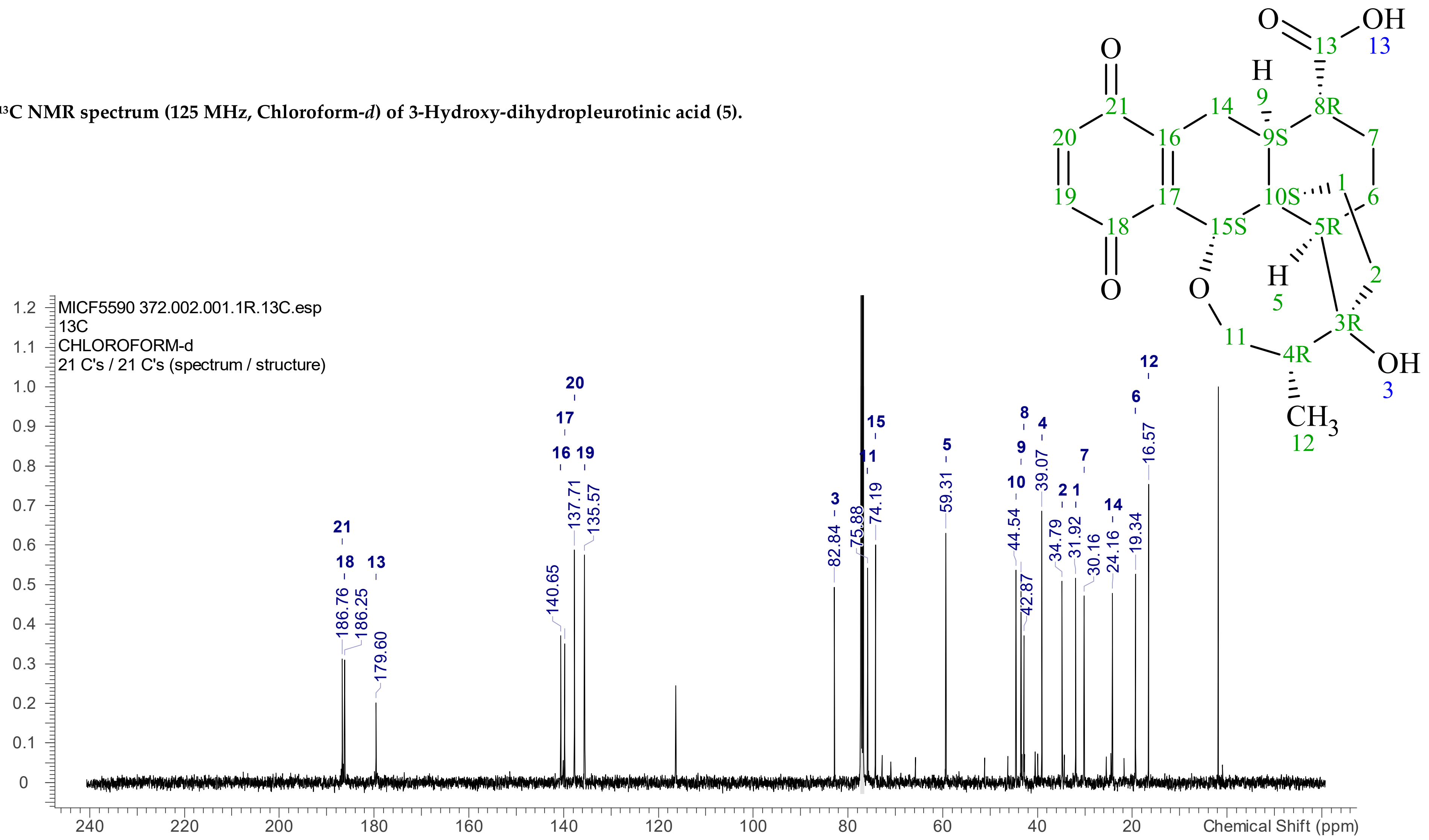

HSQC NMR spectrum (500 MHz, Chloroform-*d*) of 3-Hydroxy-dihydropleurotinic acid (5).

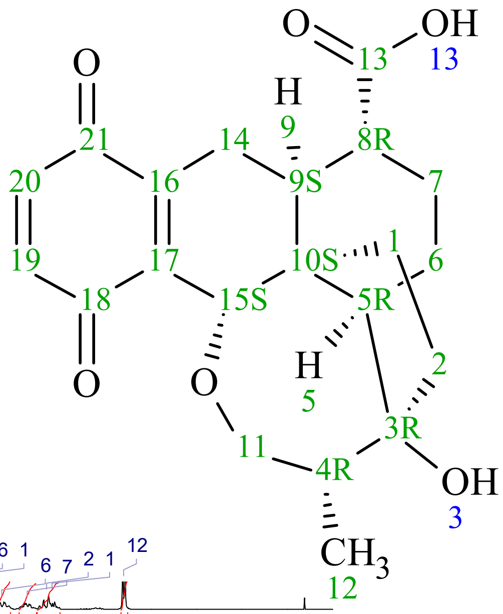

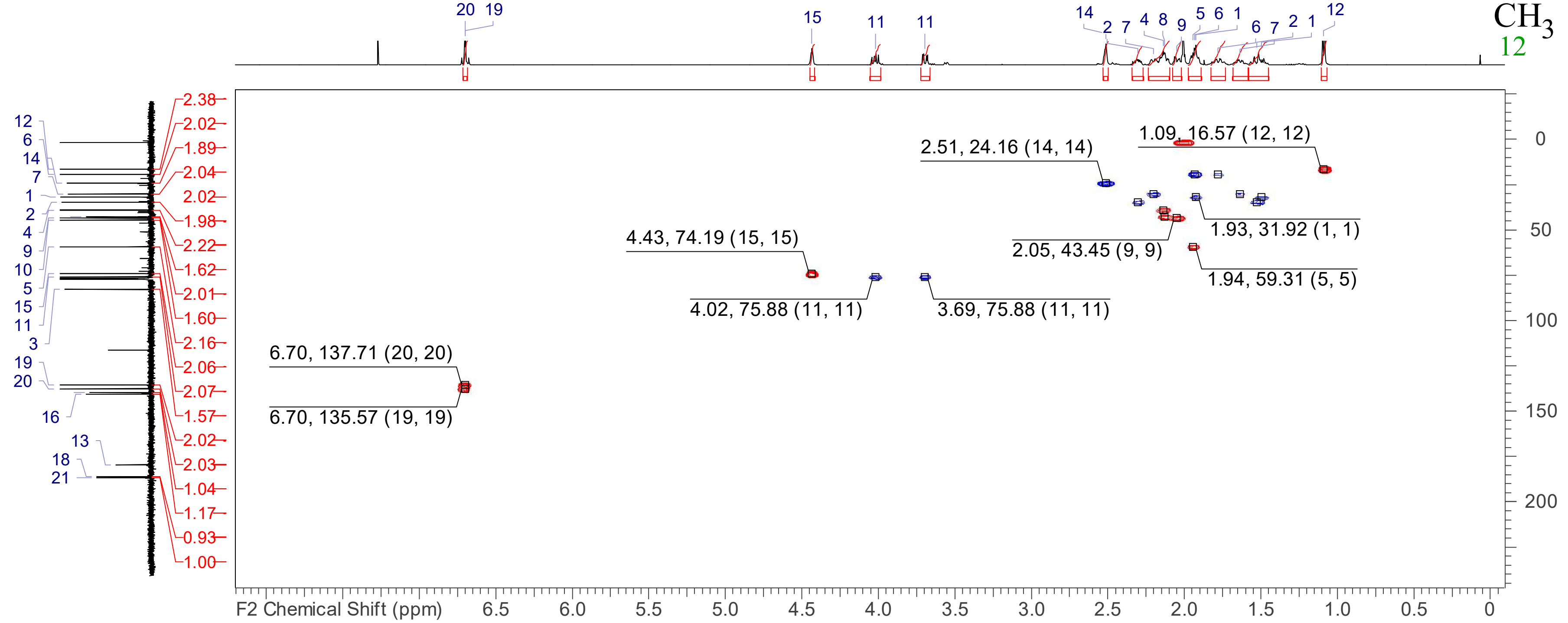

^{1}H, ^{13}C HMBC NMR spectrum (500 MHz, Chloroform-*d*) of 3-Hydroxy-dihydropleurotinic acid (5).

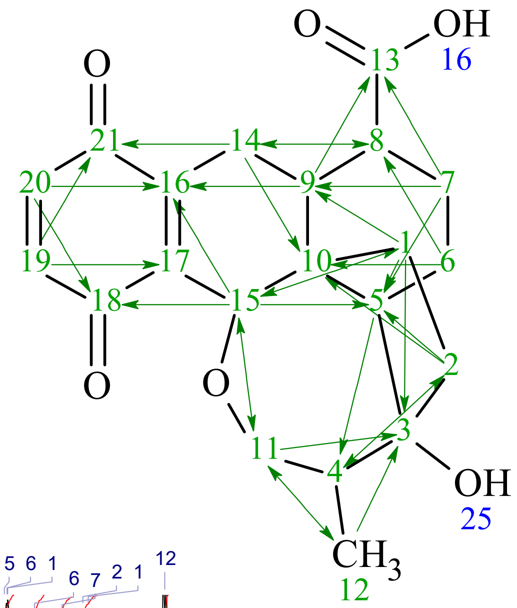

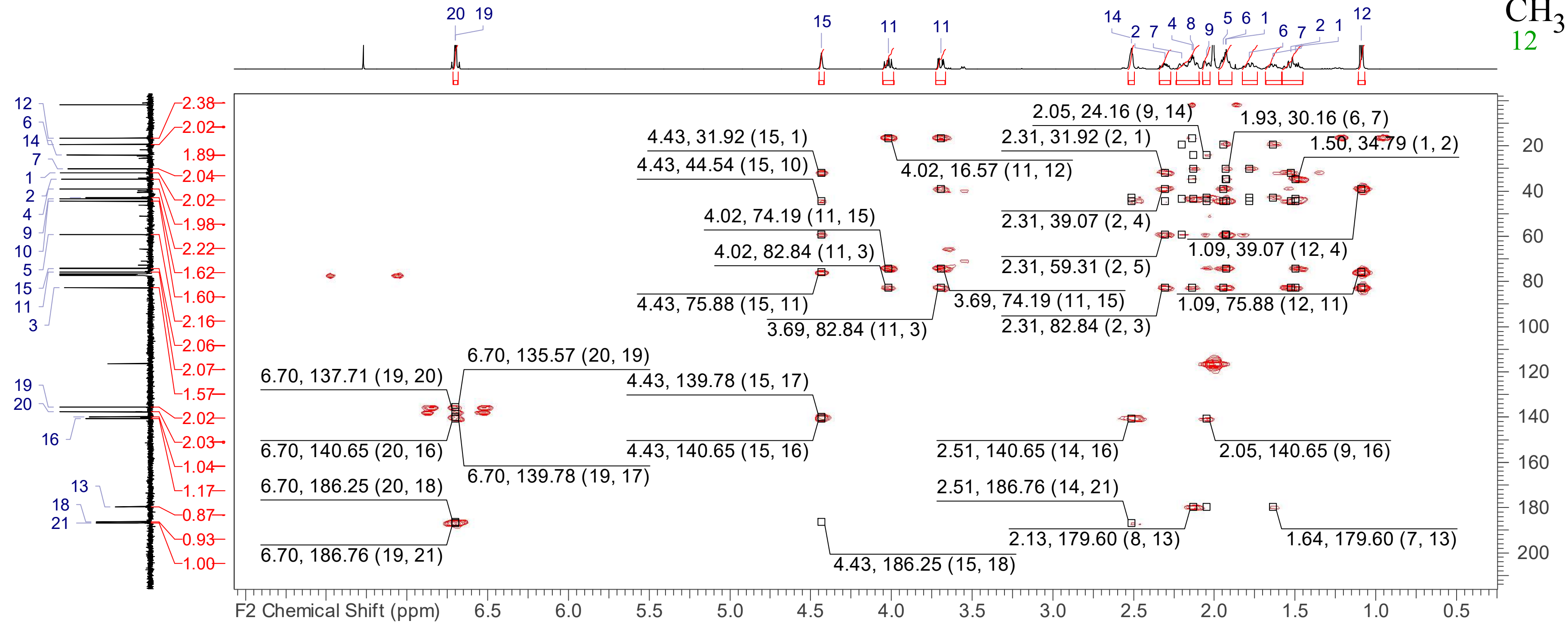

^{1}H, ^{1}H COSY NMR spectrum (500 MHz, Chloroform-*d*) of 3-Hydroxy-dihydropleurotinic acid (5).

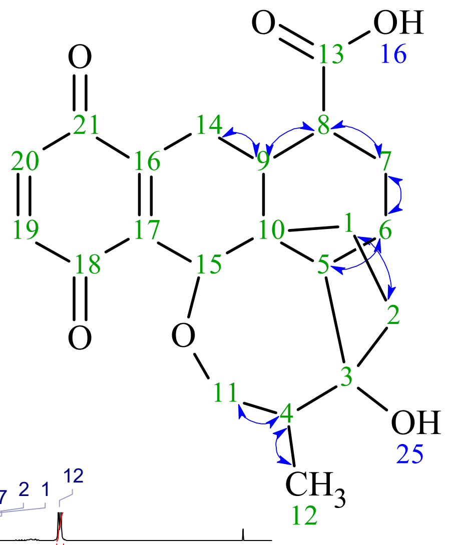

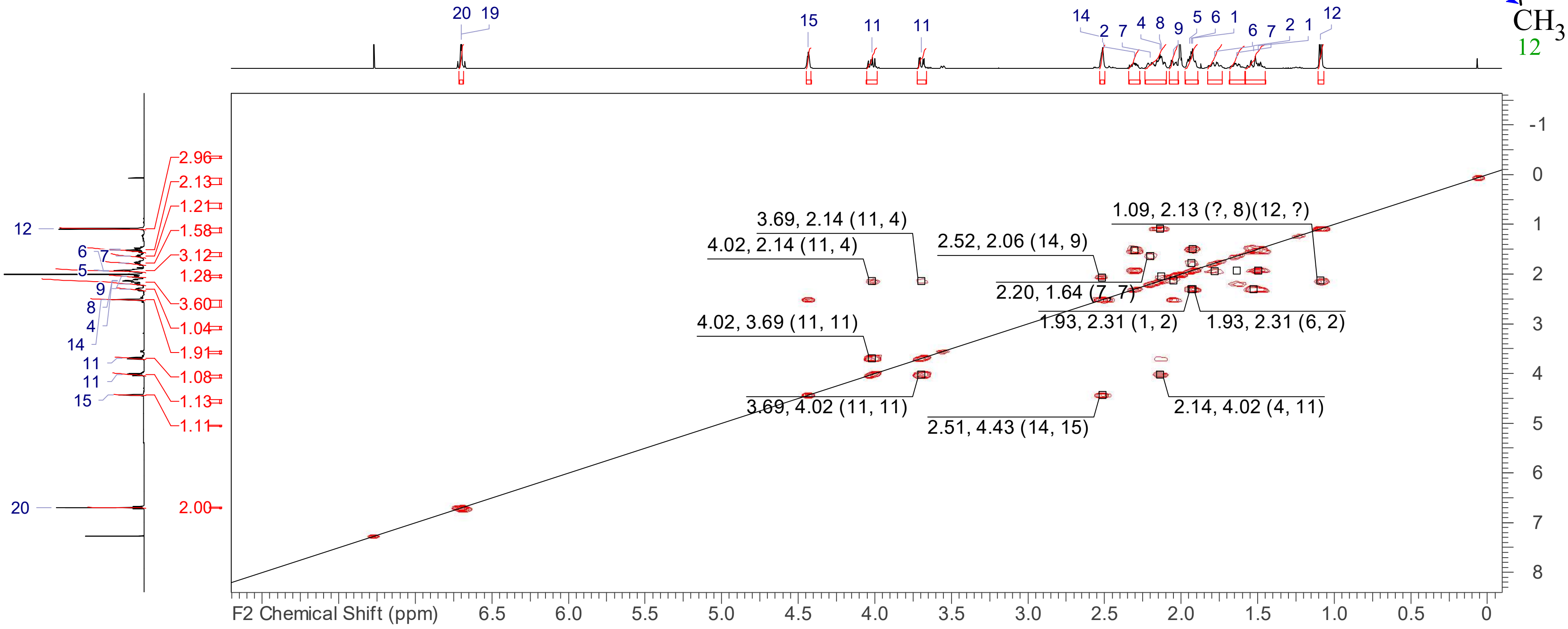

^{1}H, ^{1}H ROESY NMR spectrum (500 MHz, Chloroform-*d*) of 3-Hydroxy-dihydropleurotinic acid (5).

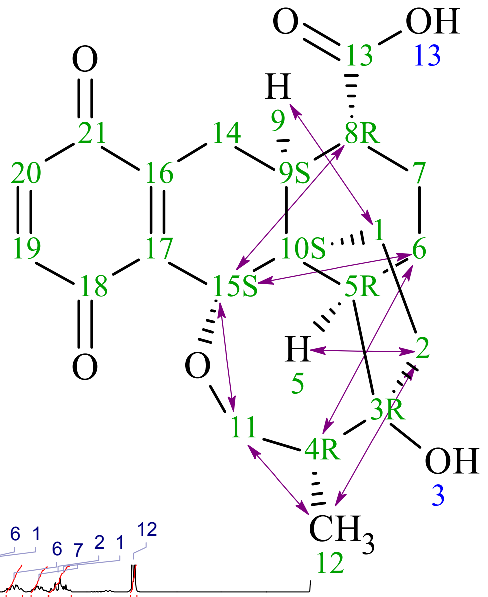

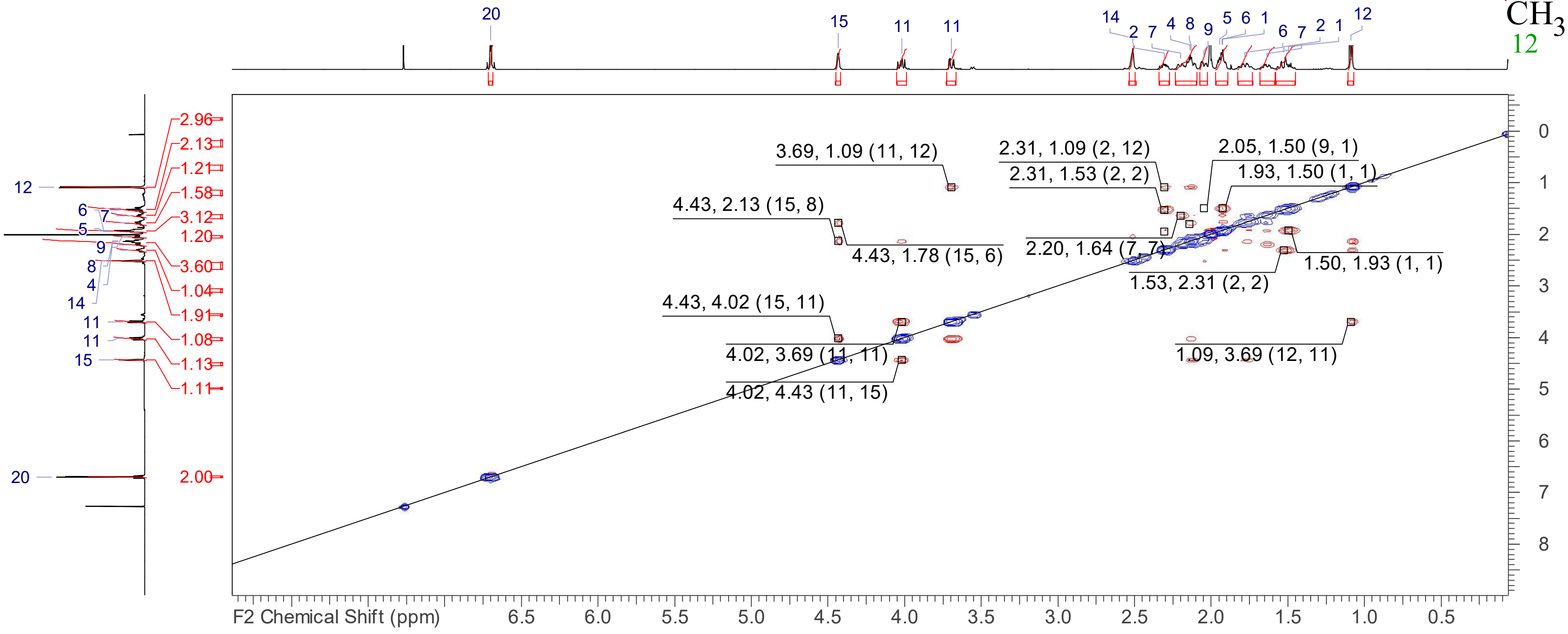

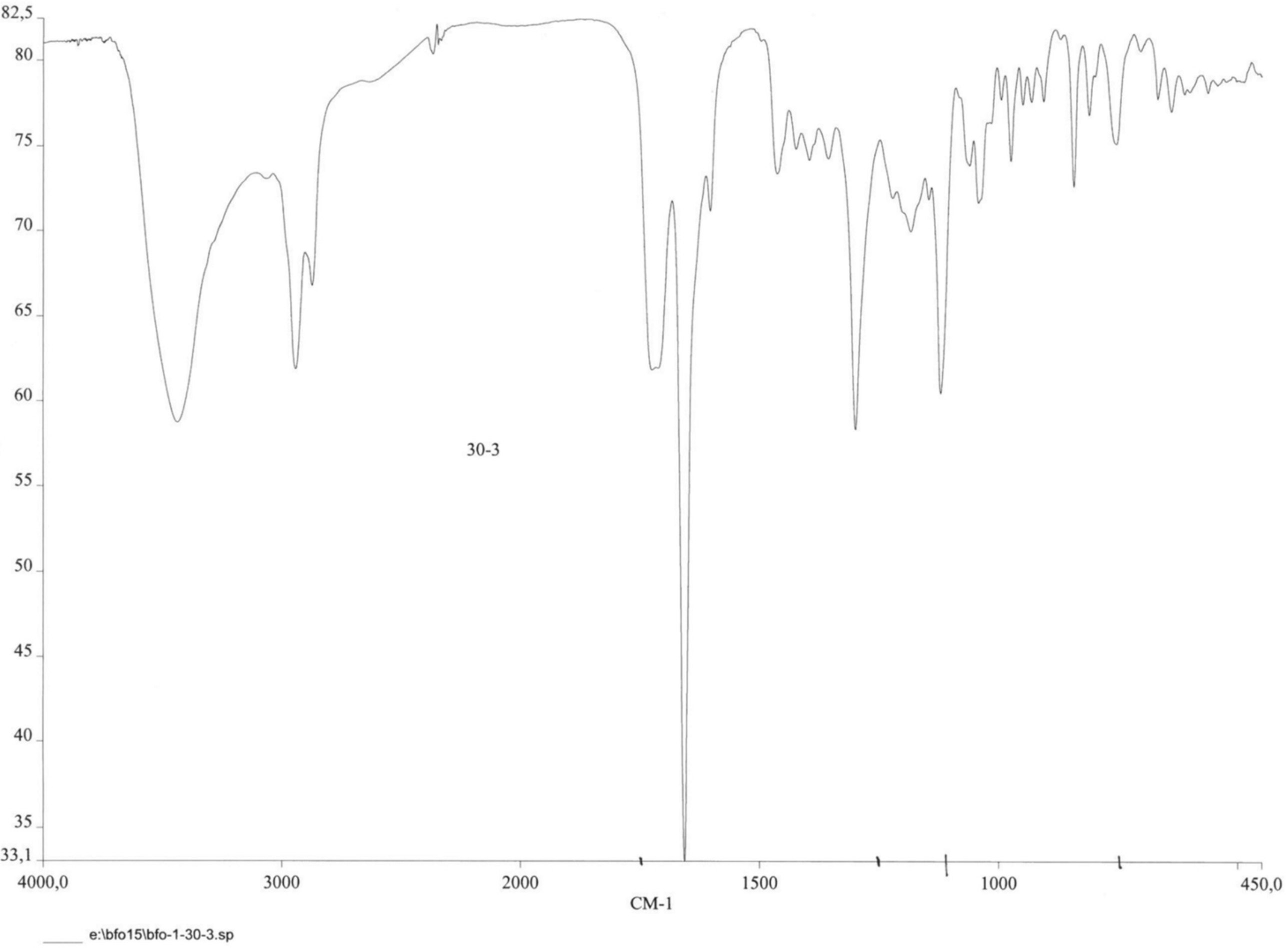

82,5
80
75
70
65
60
55
50
45
40
35
33,1
30-3
4000,0
3000
2000
1500
1000
450,0
CM-1
e:\bfo15\bfo-1-30-3.sp

Figure S3: IR spectrum (KBr) of 3-Hydroxy-dihydropleurotinic acid (5).

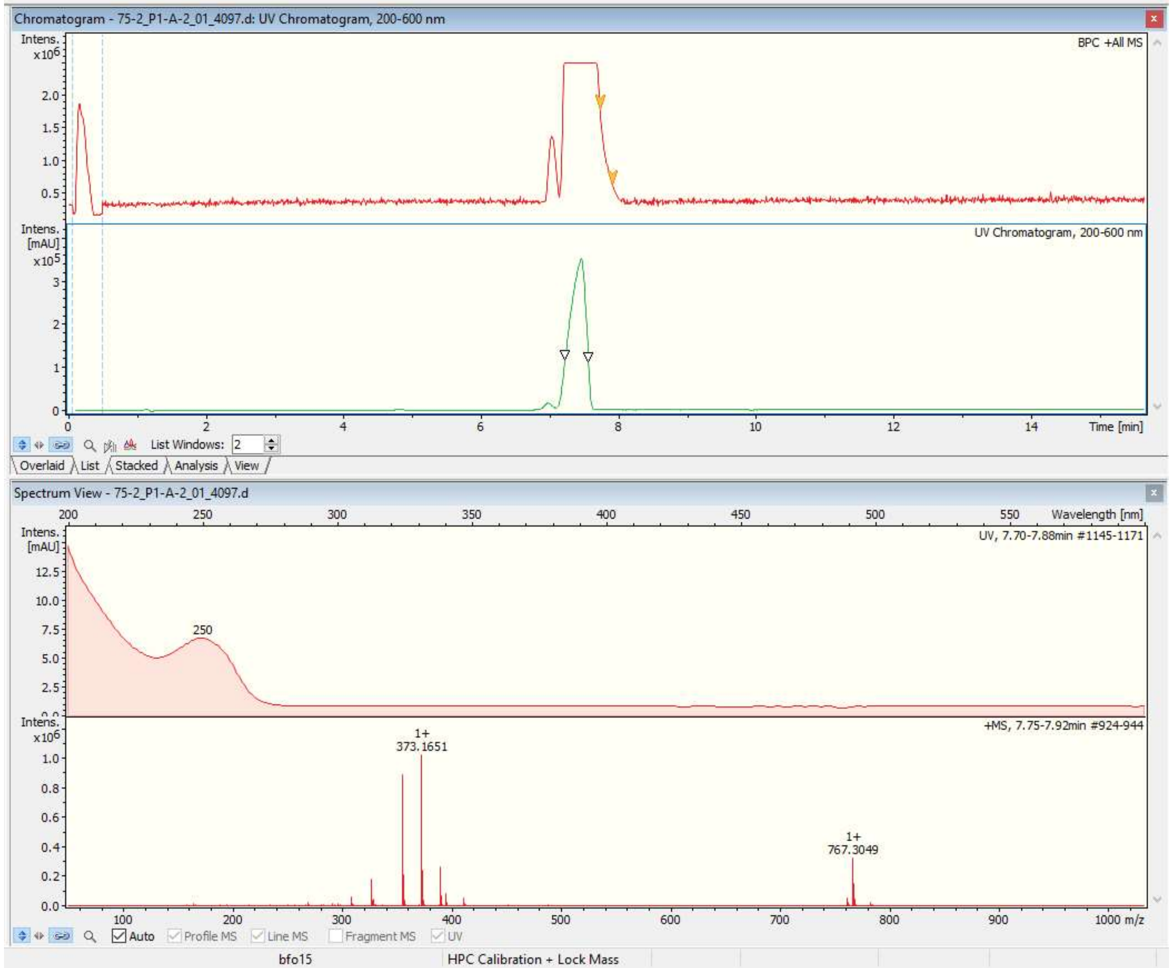

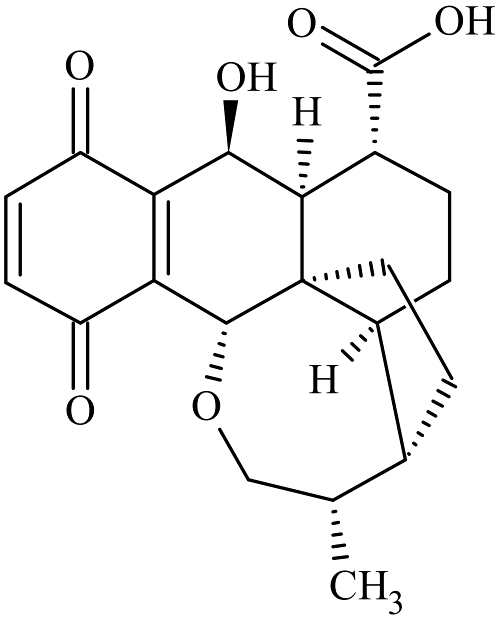

HRESIMS data of 14-Hydroxy-dihydropleurotinic acid (6)

^{1}H NMR spectrum (500 MHz, Acetone-*d*6) of 14-Hydroxy-dihydropleurotinic acid (6).

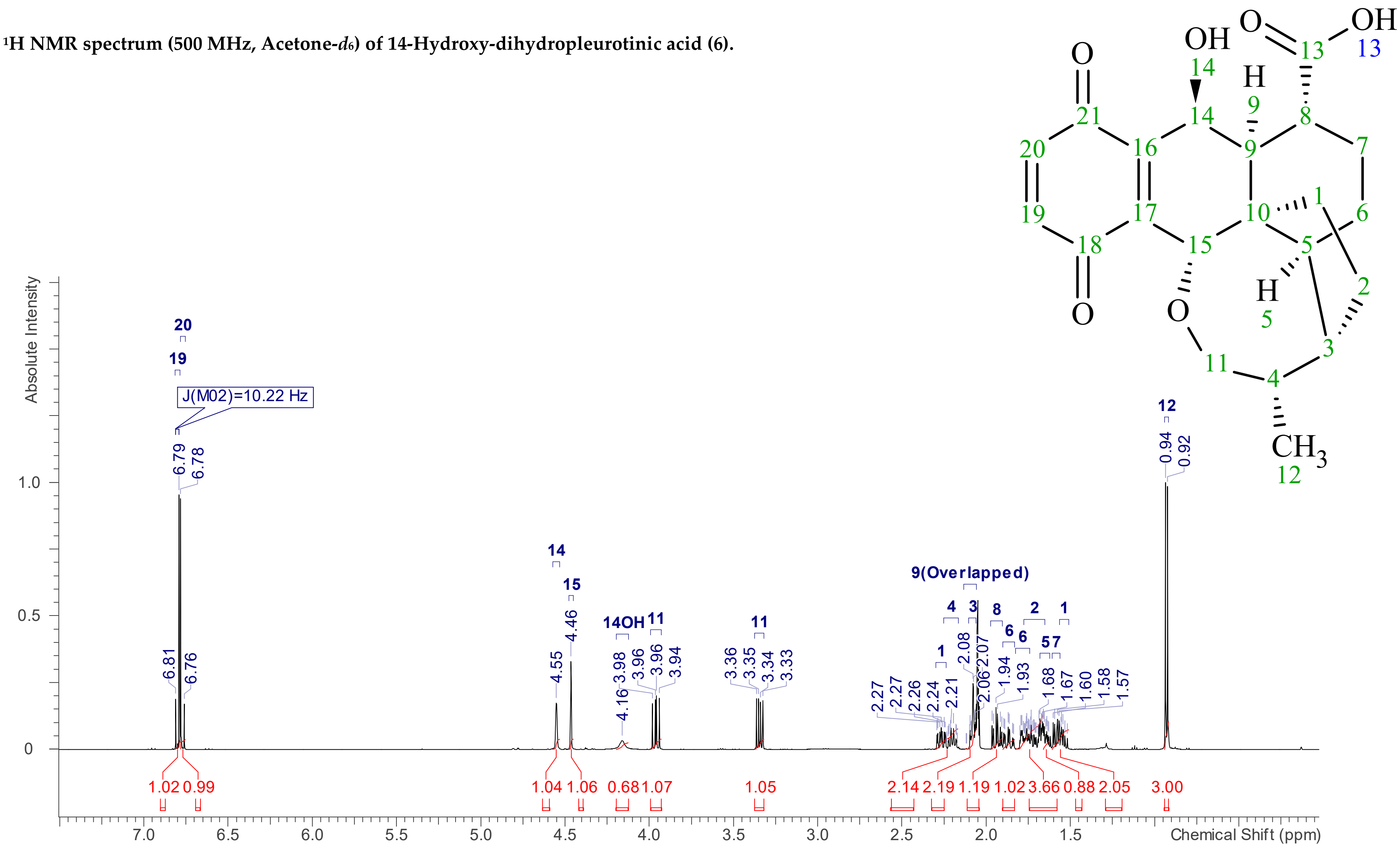

^{13}C NMR spectrum (125 MHz, Acetone-*d*6) of 14-Hydroxy-dihydropleurotinic acid (6).

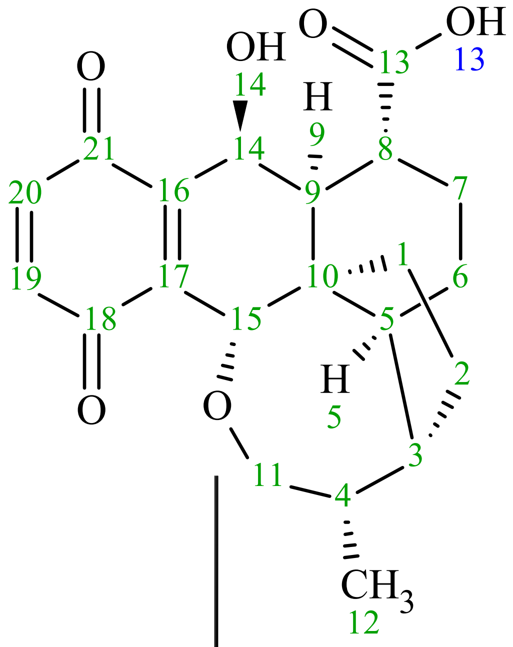

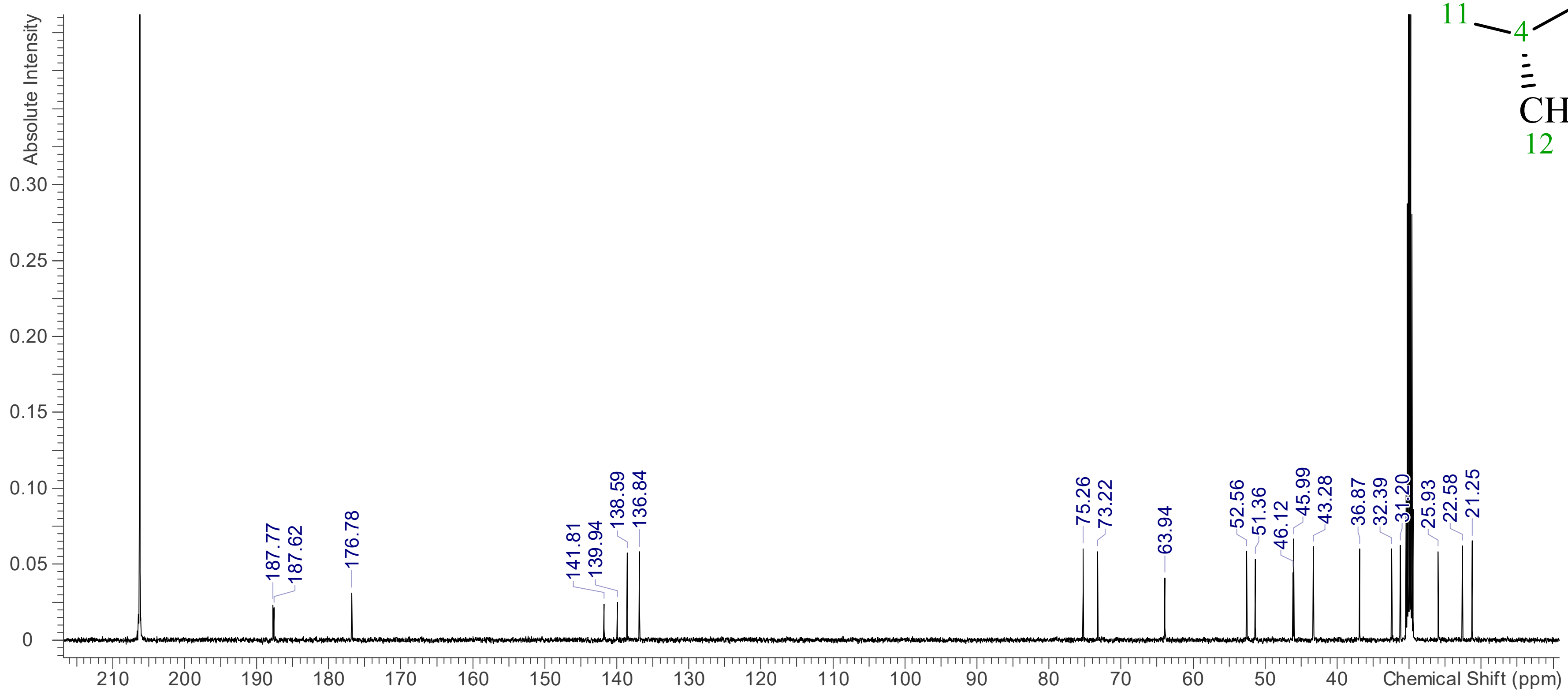

HSQC NMR spectrum (500 MHz, Acetone-*d*₆) of 14-Hydroxy-dihydropleurotinic acid (6).

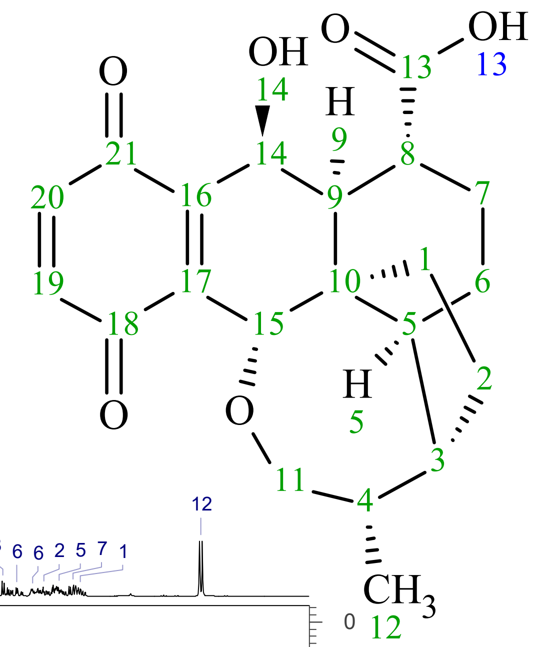

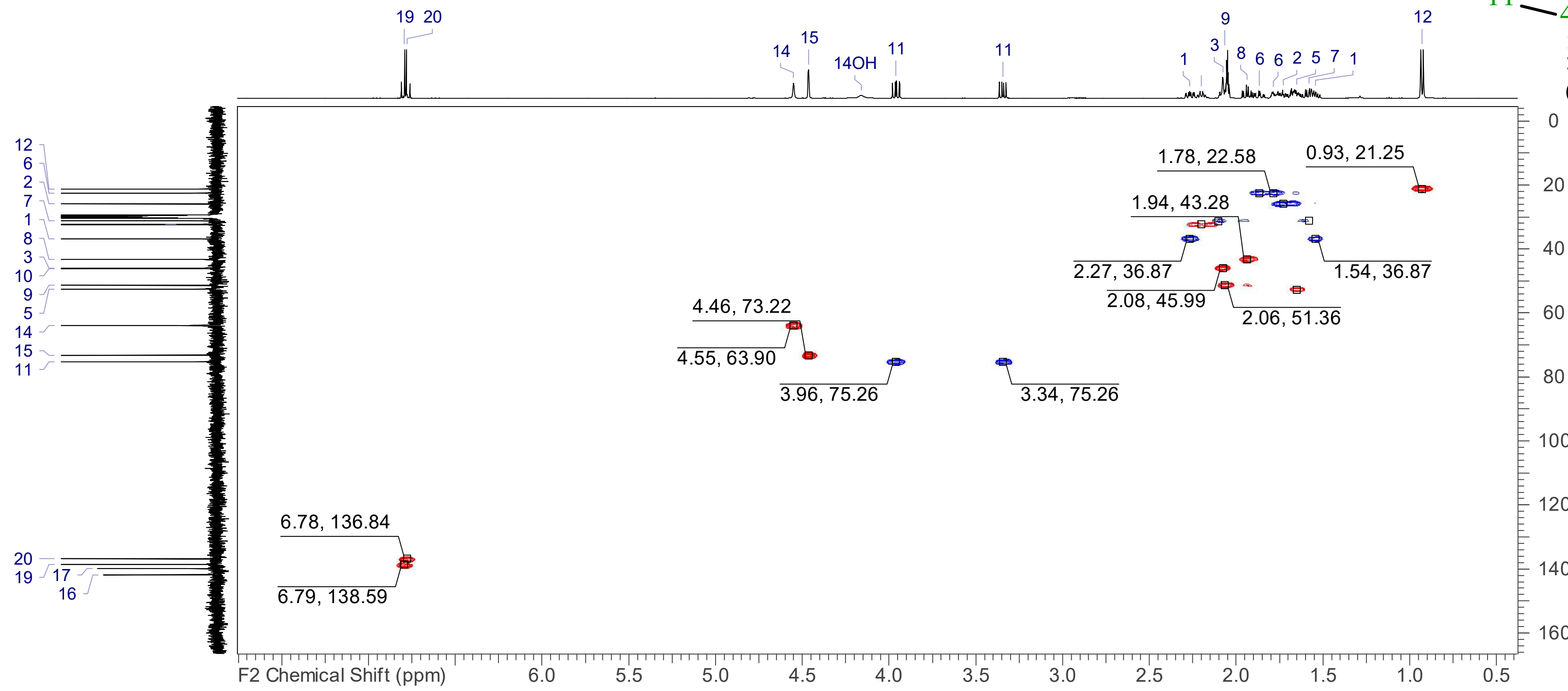

^{1}H, ^{1}H COSY NMR spectrum (500 MHz, Acetone-*d*$_6$) of 14-Hydroxy-dihydropleurotinic acid (6).

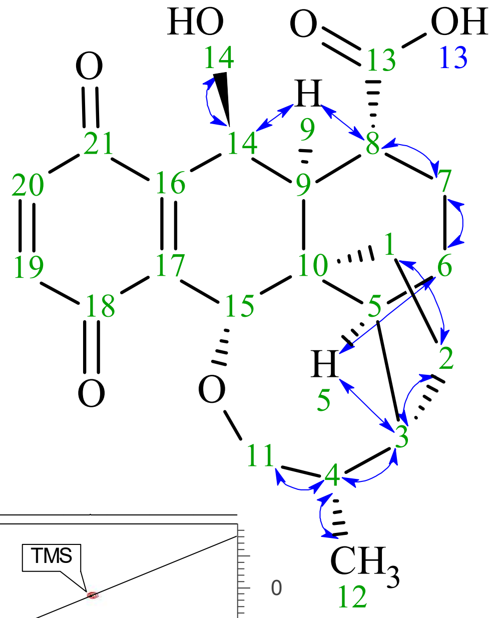

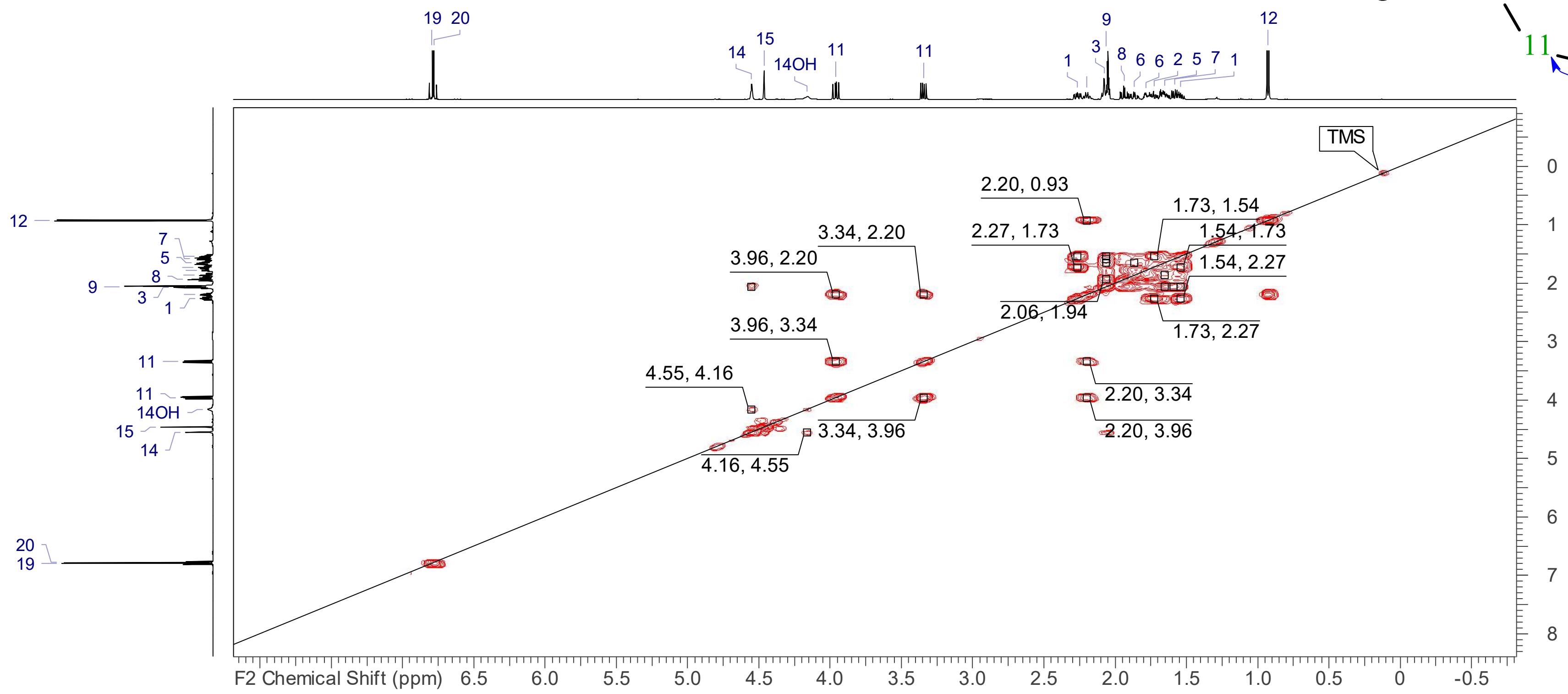

HMBC NMR spectrum (500 MHz, Acetone-d_6) of 14-Hydroxy-dihydropleurotinic acid (6).

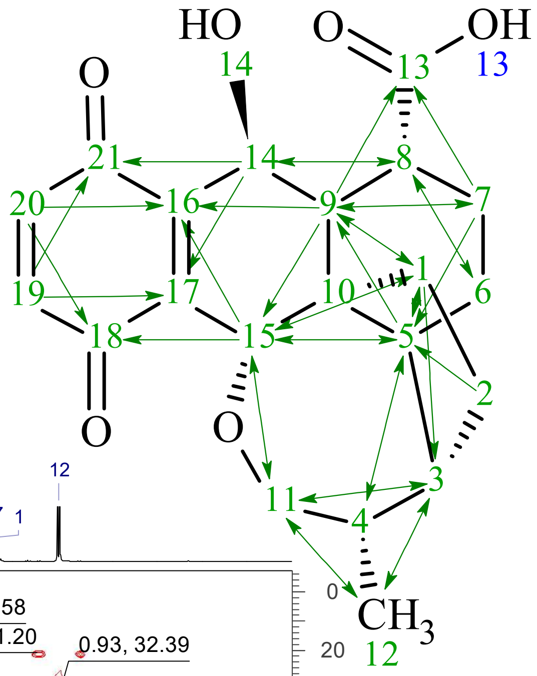

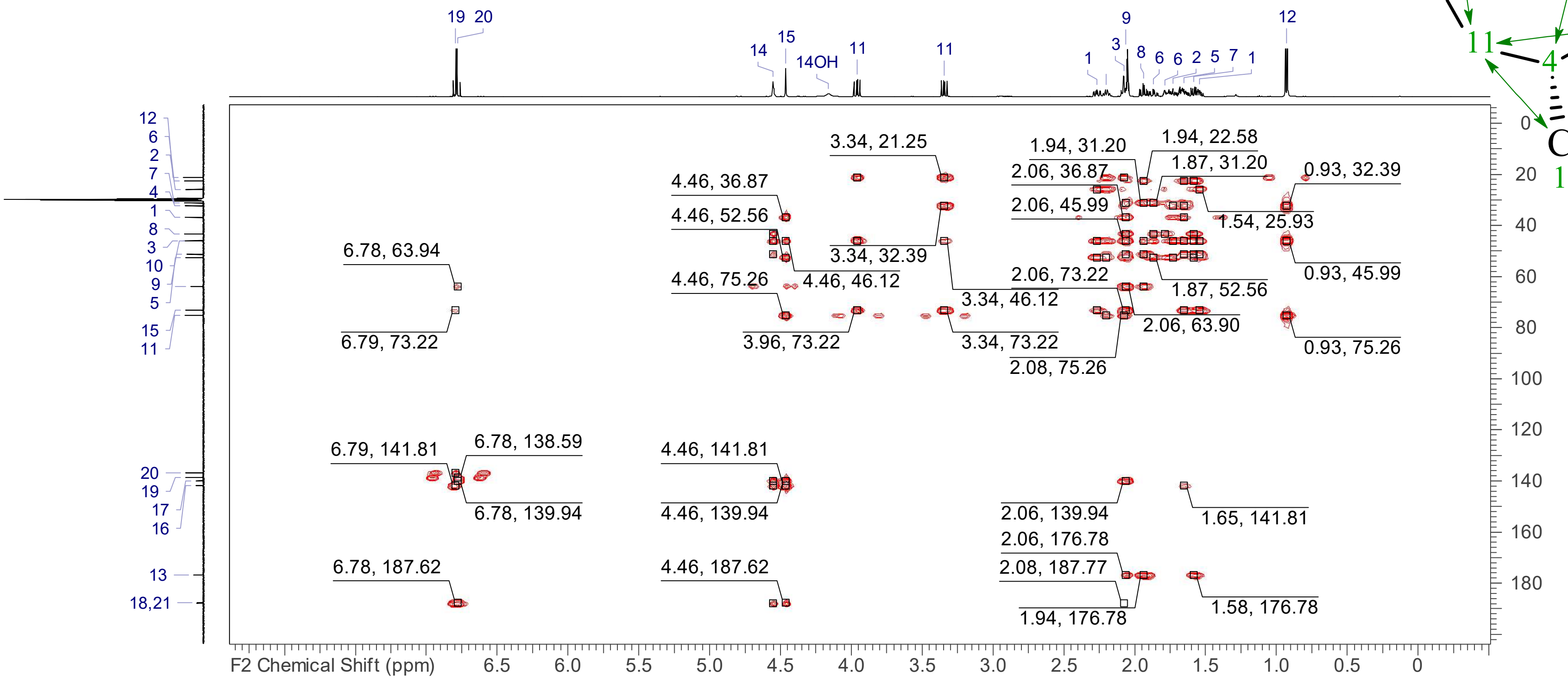

^{1}H, ^{1}H ROESY NMR spectrum (500 MHz, Acetone-*d*6) of 14-Hydroxy-dihydropleurotinic acid (6).

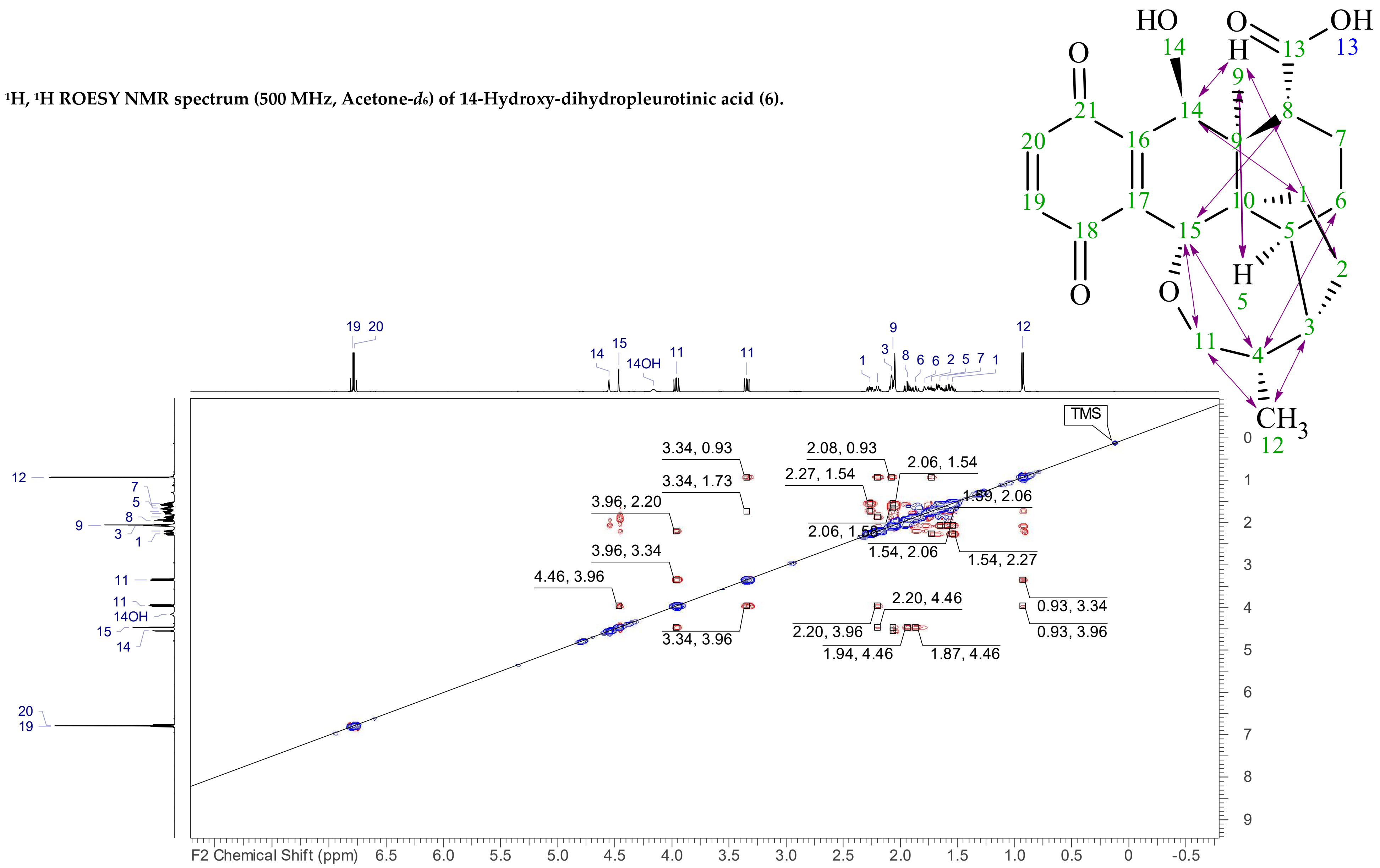

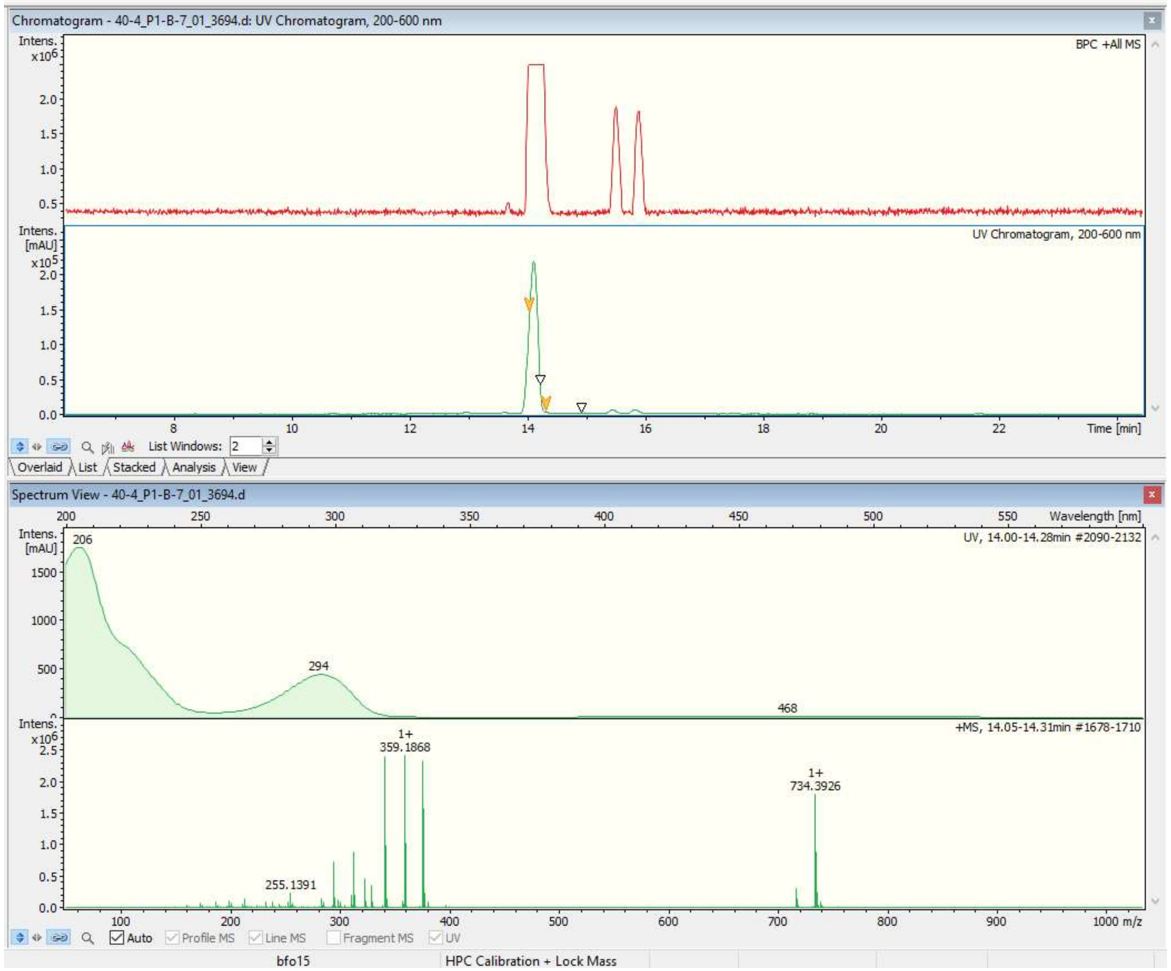

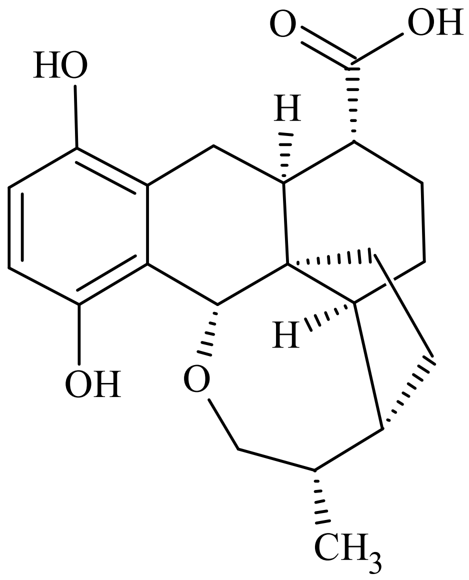

HRESIMS data of Leucopleurotinic acid (7).

^{1}H NMR spectrum (700 MHz, Acetone-*d*6) of Leucopleurotinic acid (7).

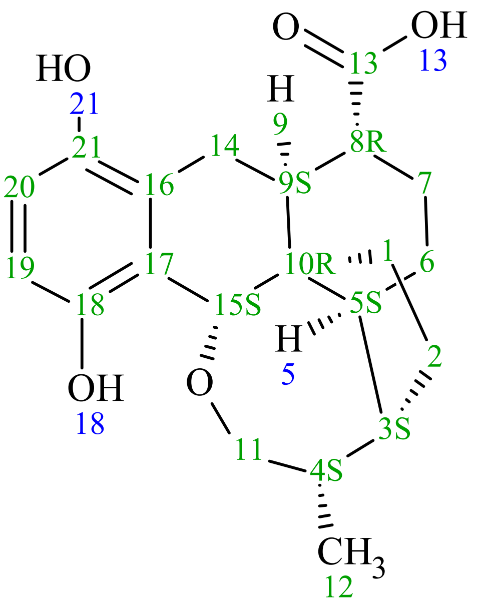

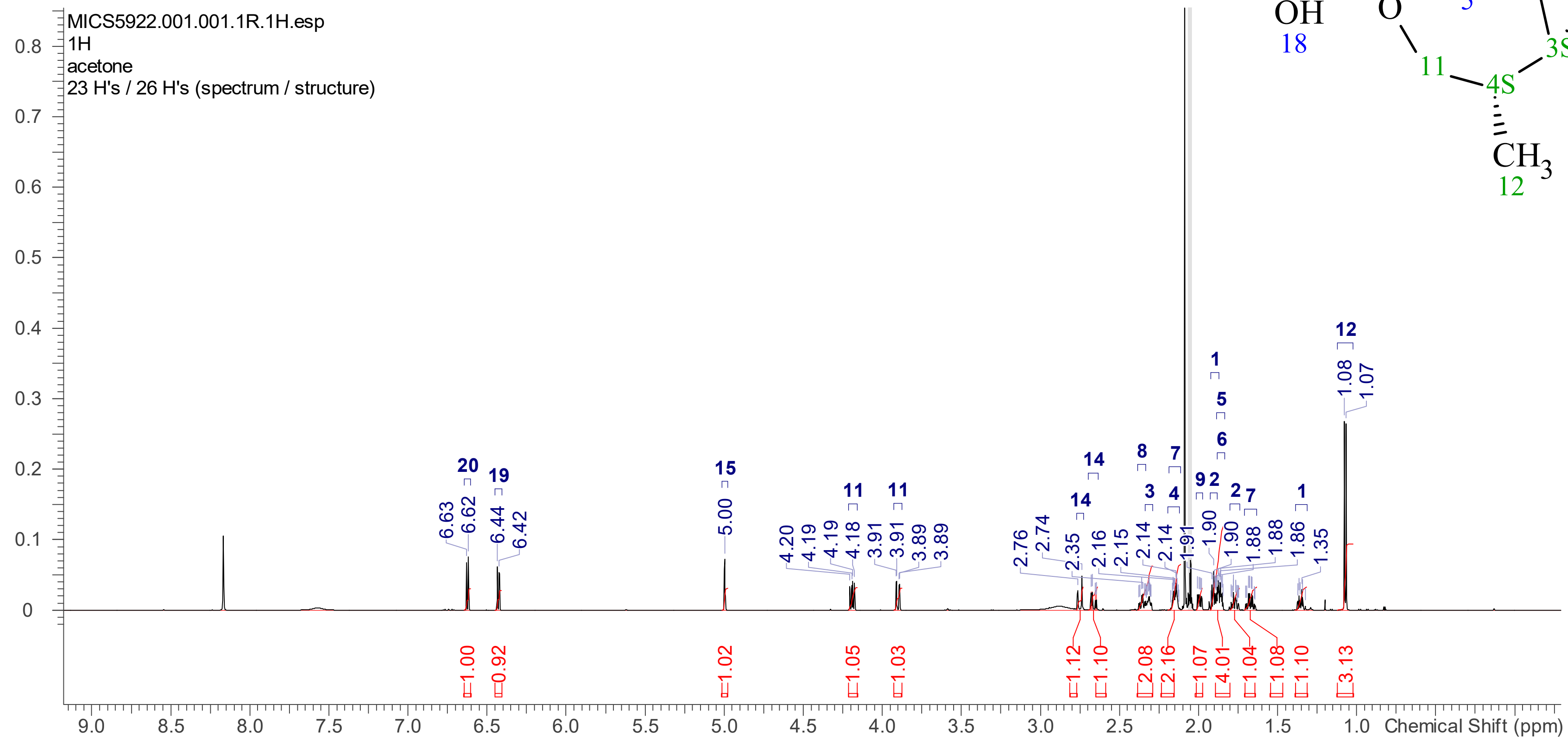

^{13}C NMR spectrum (176 MHz, Acetone-d_6) of Leucopleurotinic acid (7).

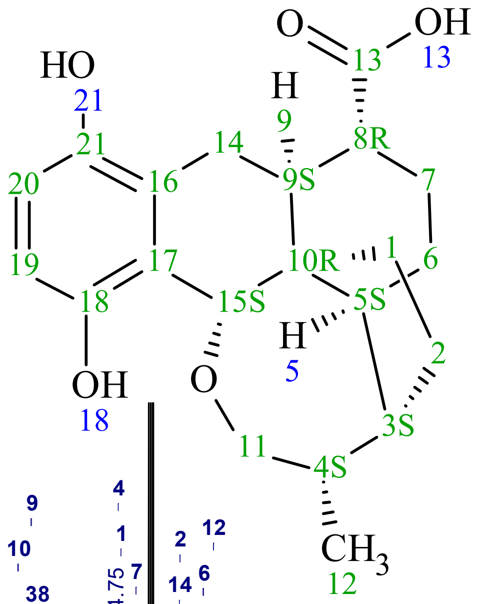

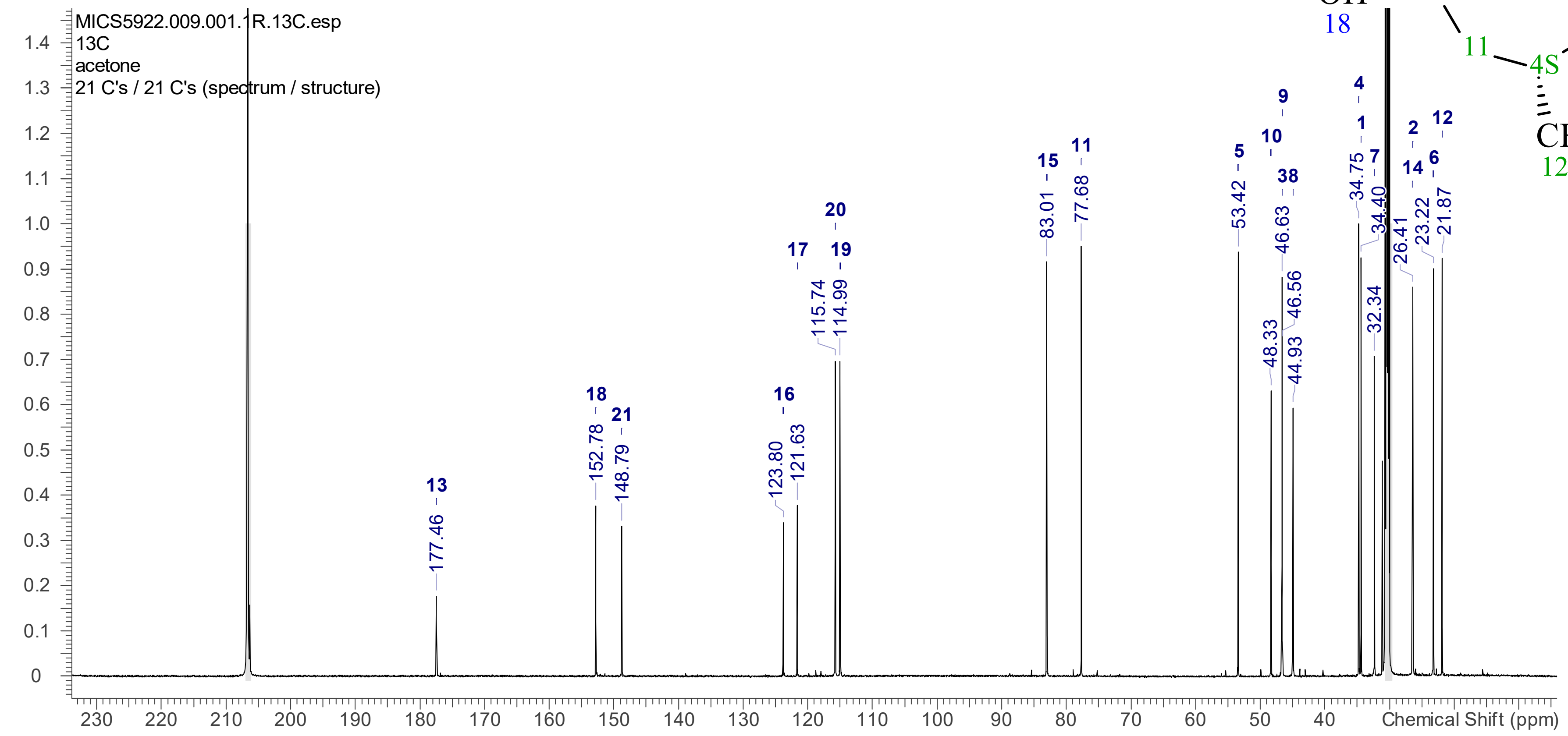

HSQC NMR spectrum (700 MHz, Acetone-d_6) of Leucopleurotinic acid (7).

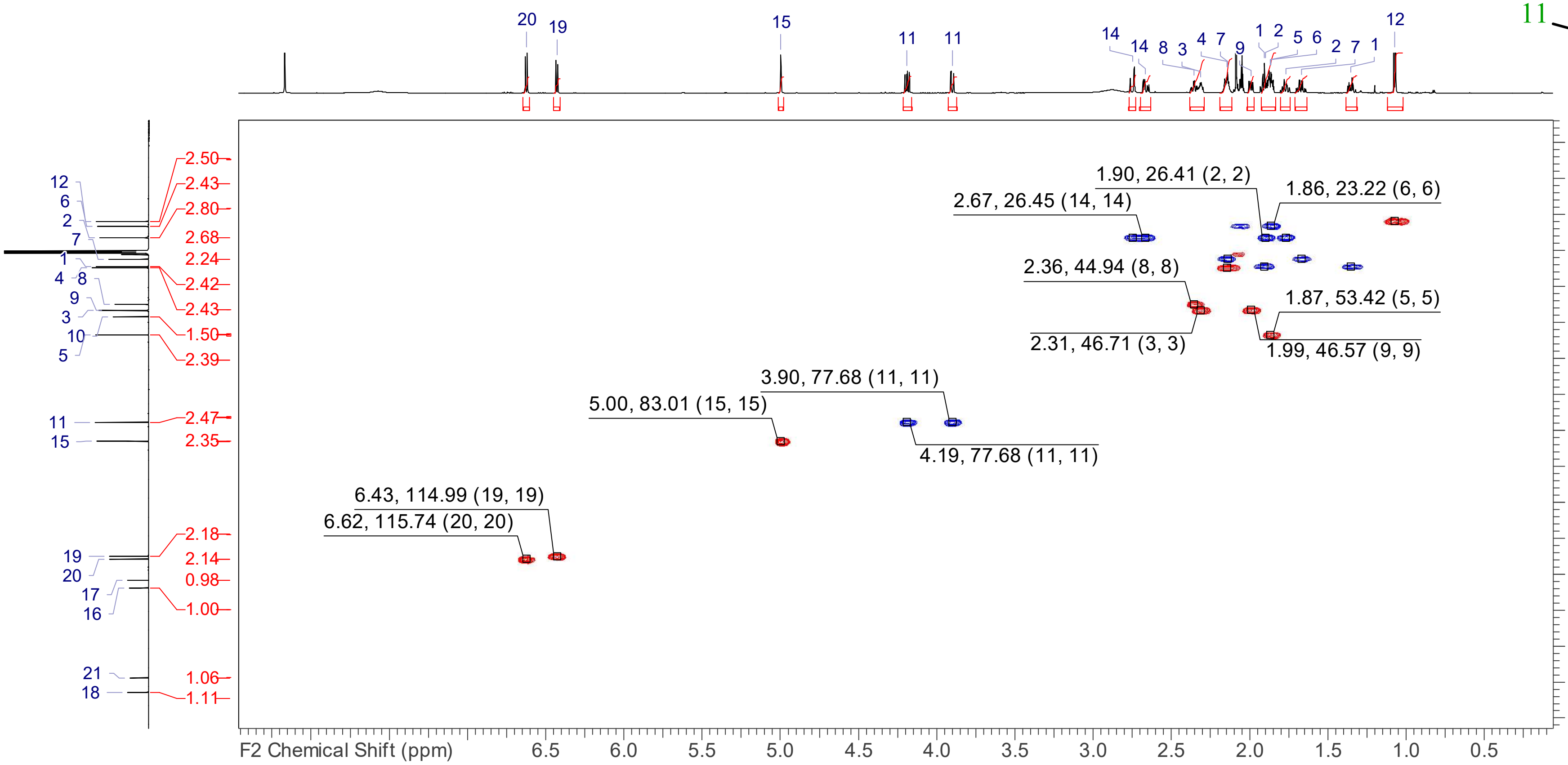

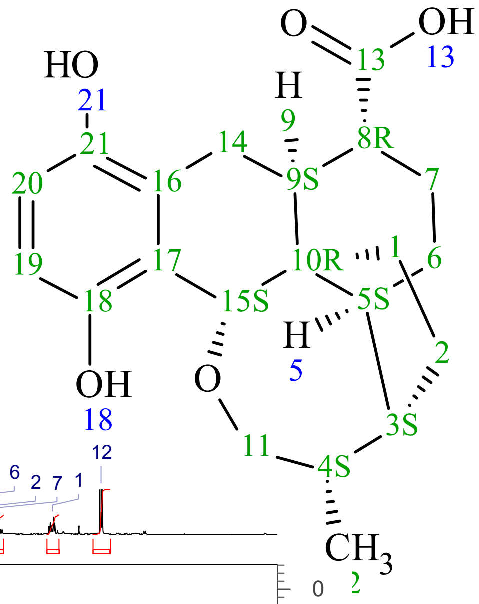

^{1}H, ^{1}H COSY NMR spectrum (700 MHz, Acetone-d_6) of Leucopleurotinic acid (7).

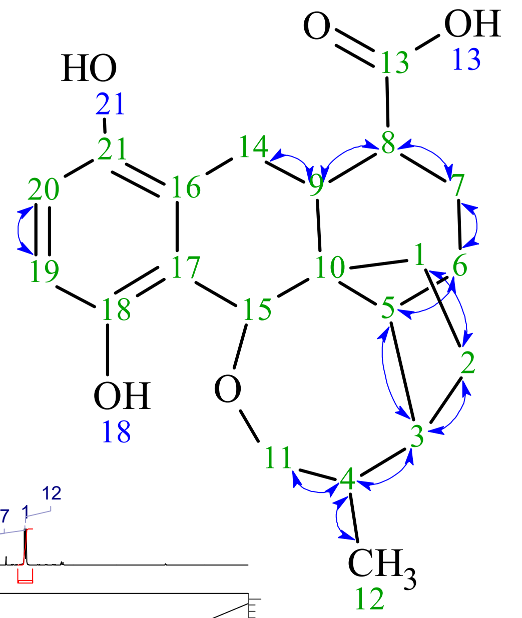

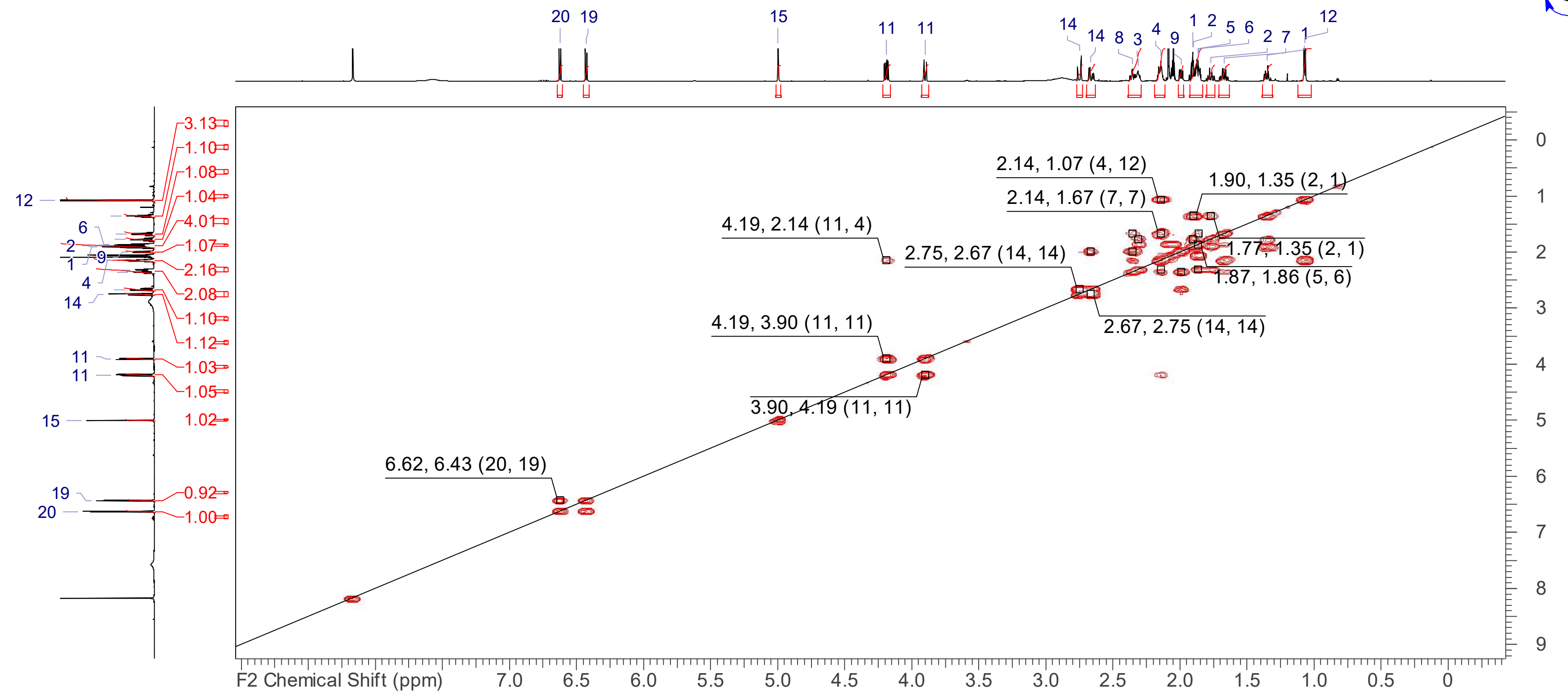

HMBC NMR spectrum (700 MHz, Acetone-*d*$_6$) of Leucopleurotinic acid (7).

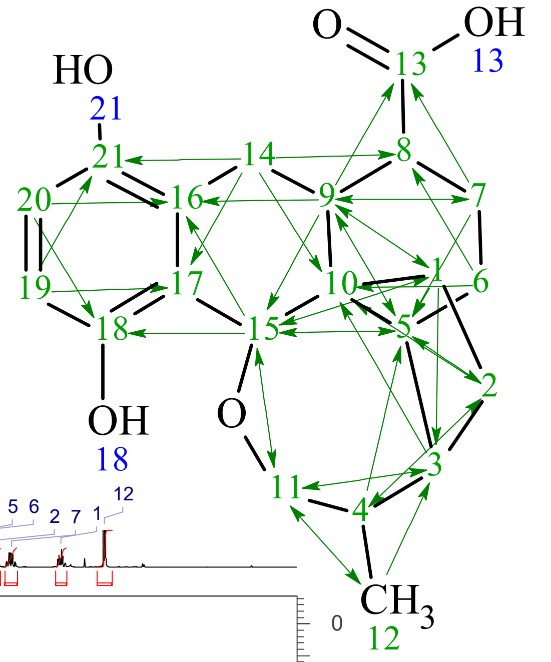

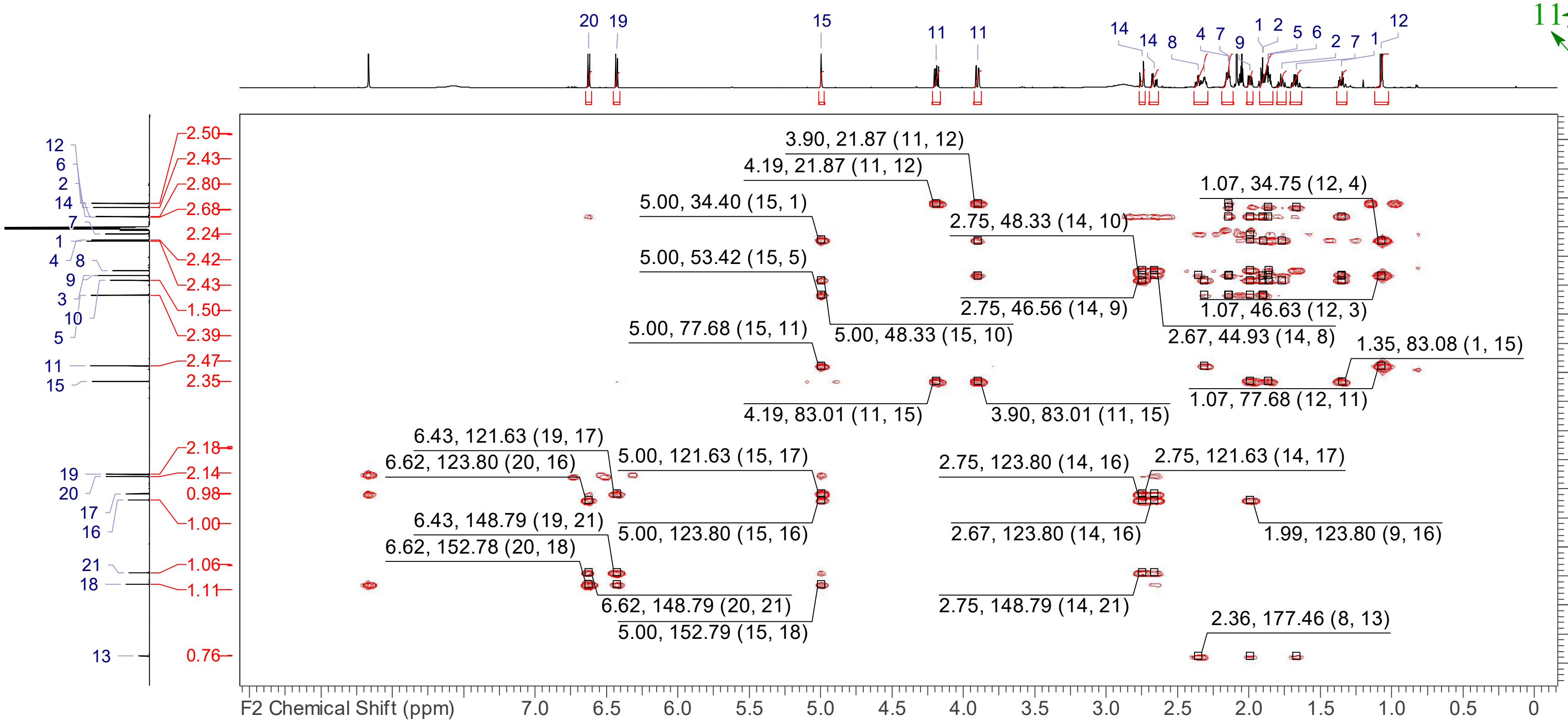

¹H, ¹H ROESY NMR spectrum (700 MHz, Acetone-d_6) of Leucopleurotinic acid (7).

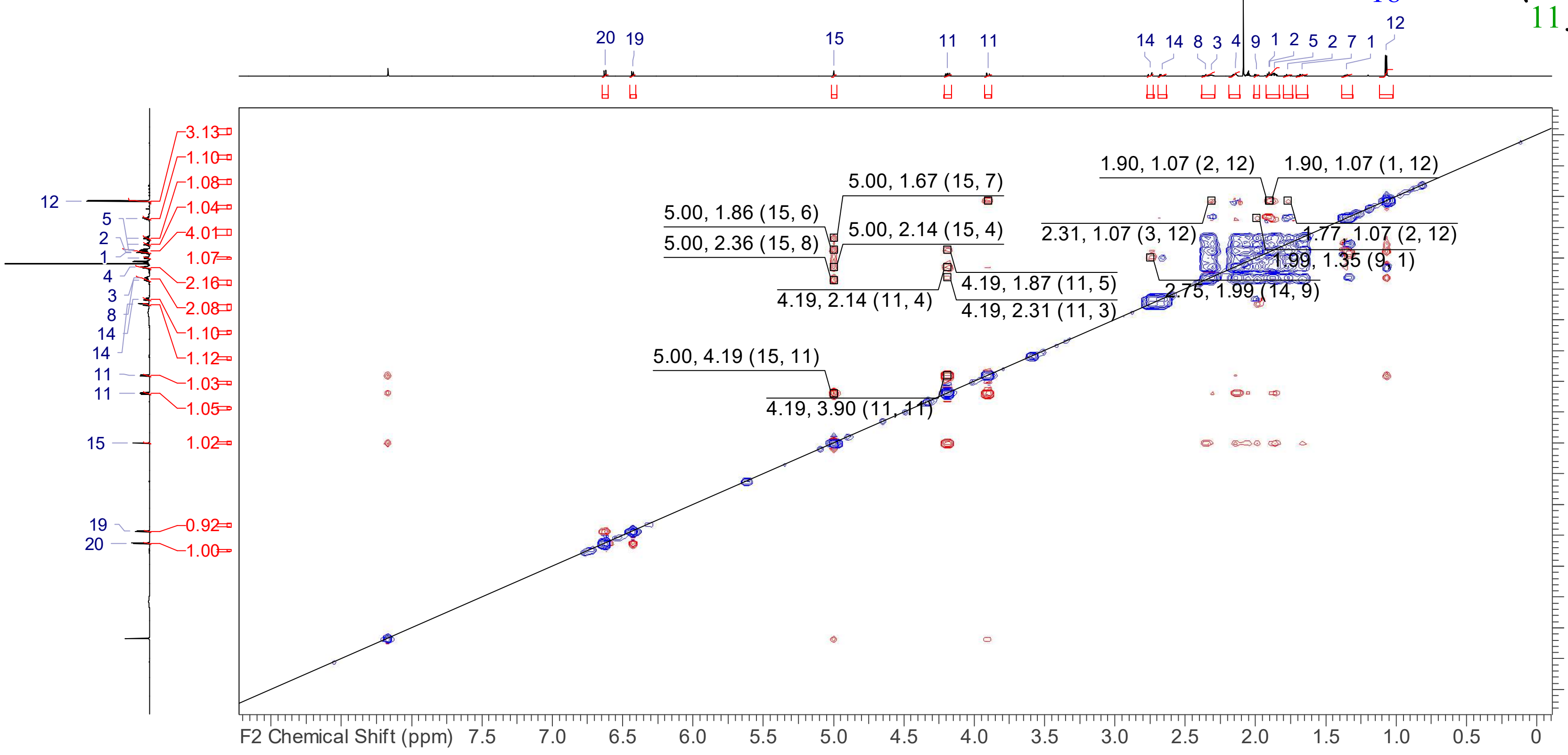

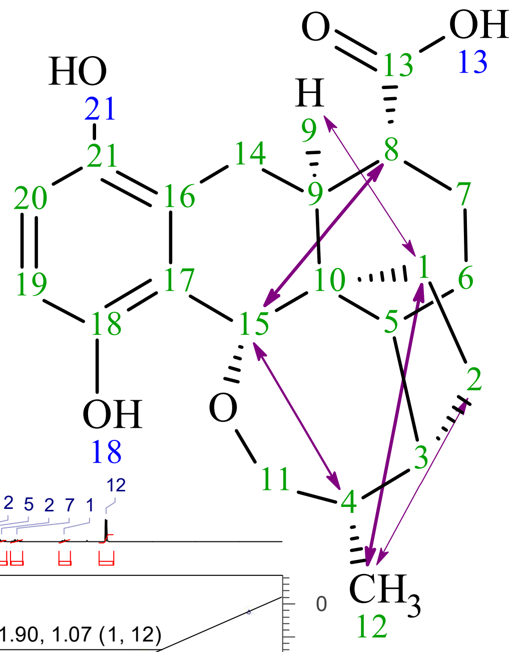

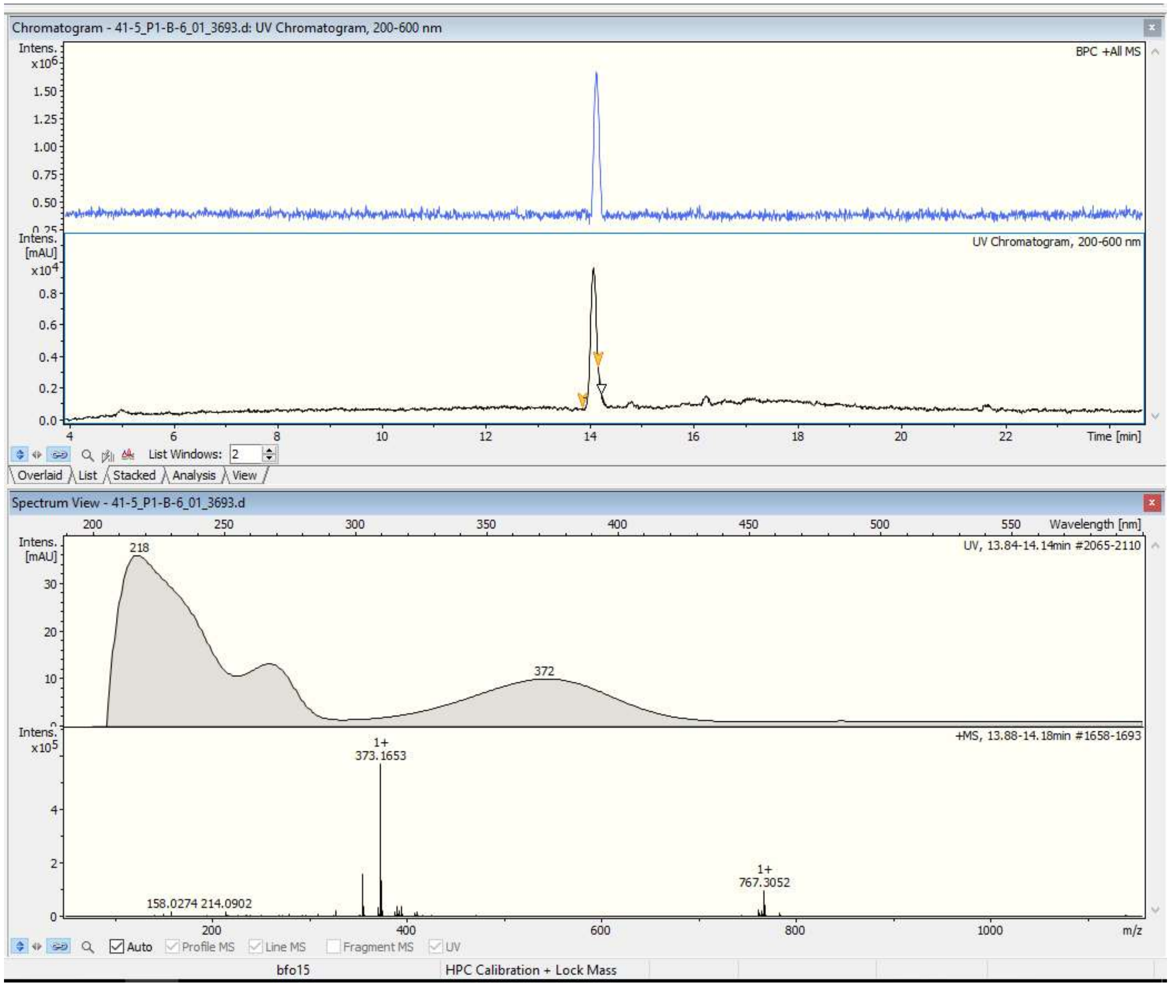

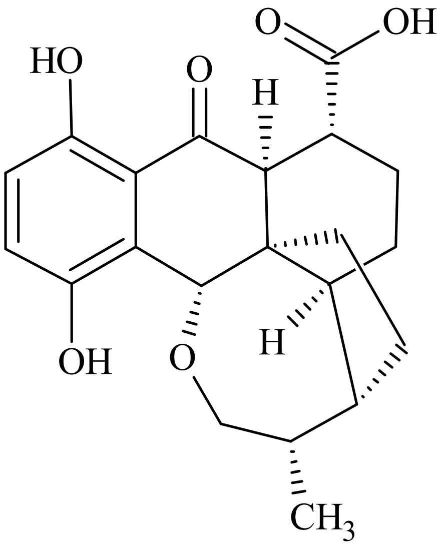

HRESIMS data of 14-Oxo-leucopleurotinic acid (8).

¹H NMR spectrum (700 MHz, Chloroform-*d*) of 14-Oxo-leucopleurotinic acid (8).

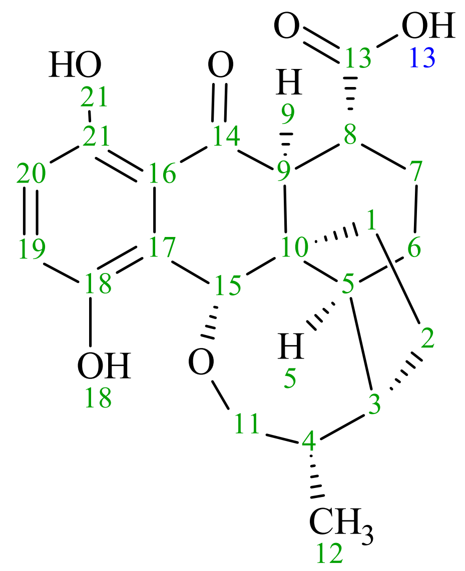

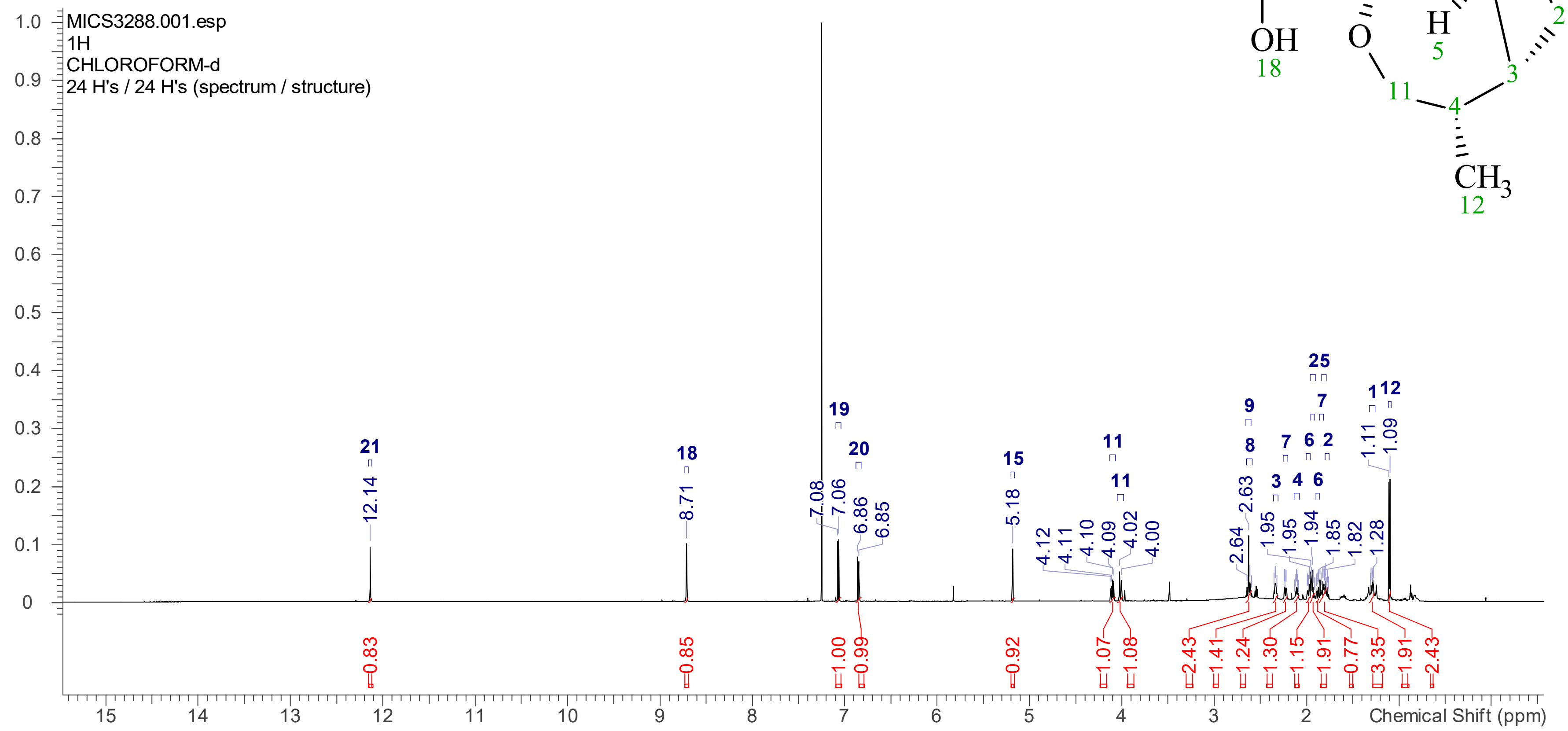

^{13}C NMR spectrum (176 MHz, Chloroform-*d*) of 14-Oxo-leucopleurotinic acid (8).

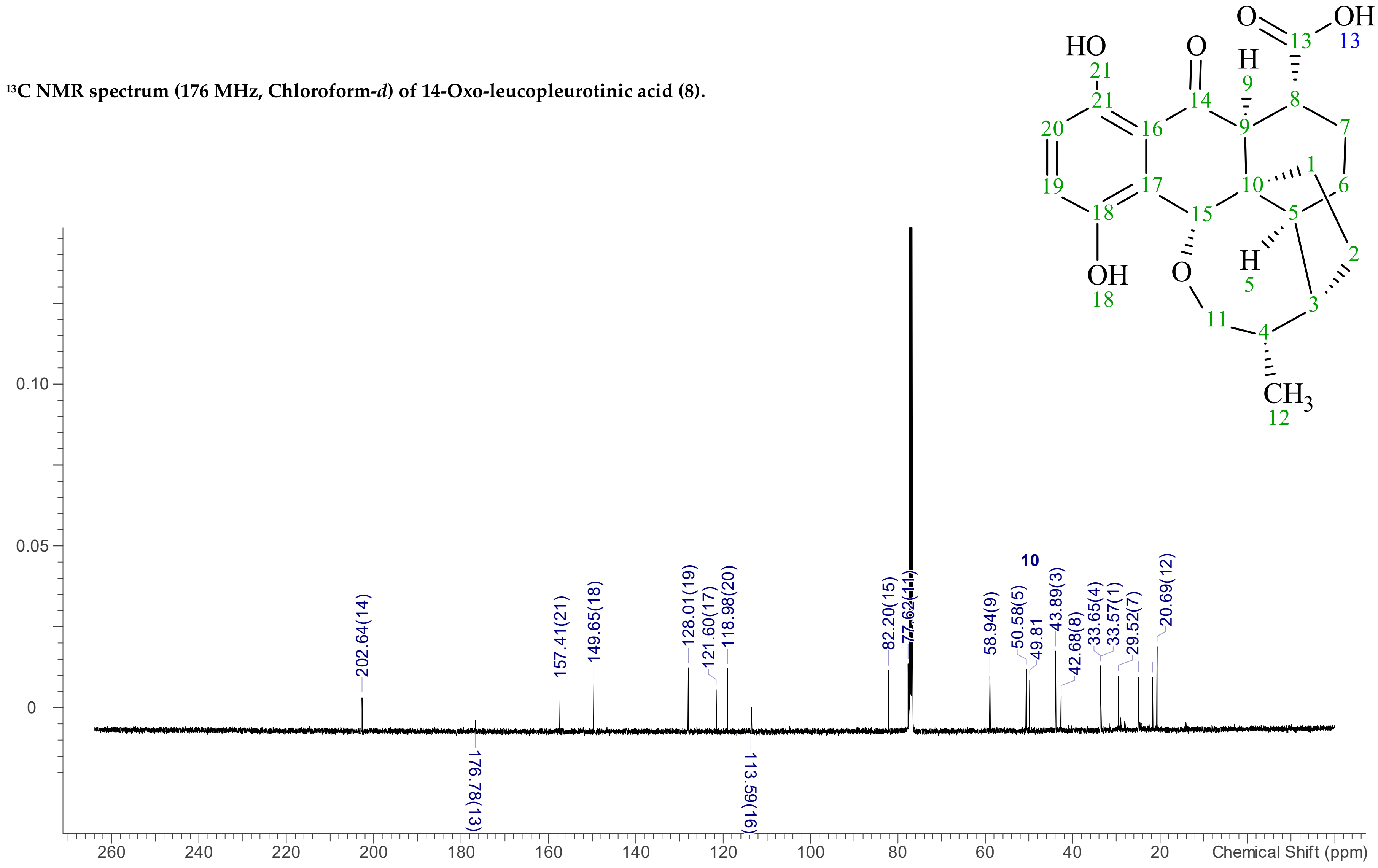

¹H, ¹H COSY NMR spectrum (700 MHz, Chloroform-*d*) of 14-Oxo-leucopleurotinic acid (8).

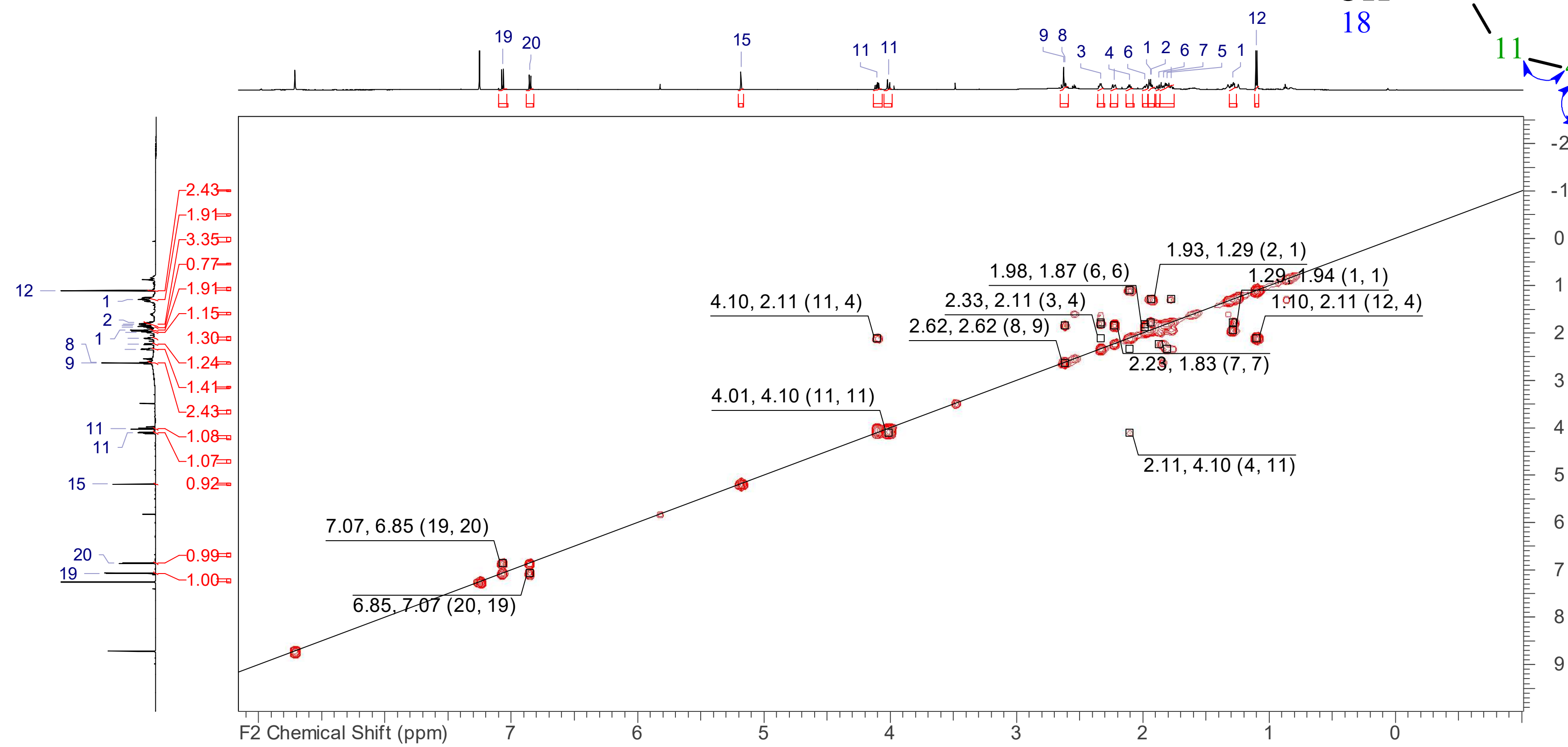

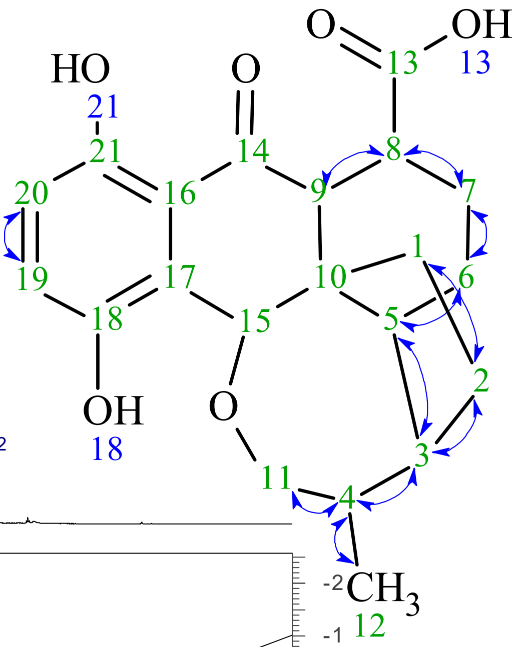

HSQC NMR spectrum (700 MHz, Chloroform-*d*) of 14-Oxo-leucopleurotinic acid (8).

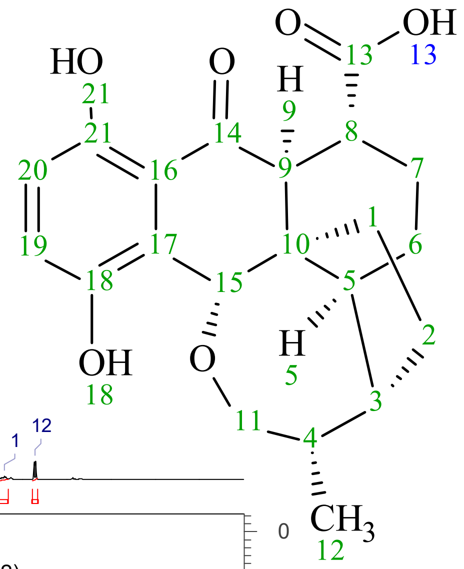

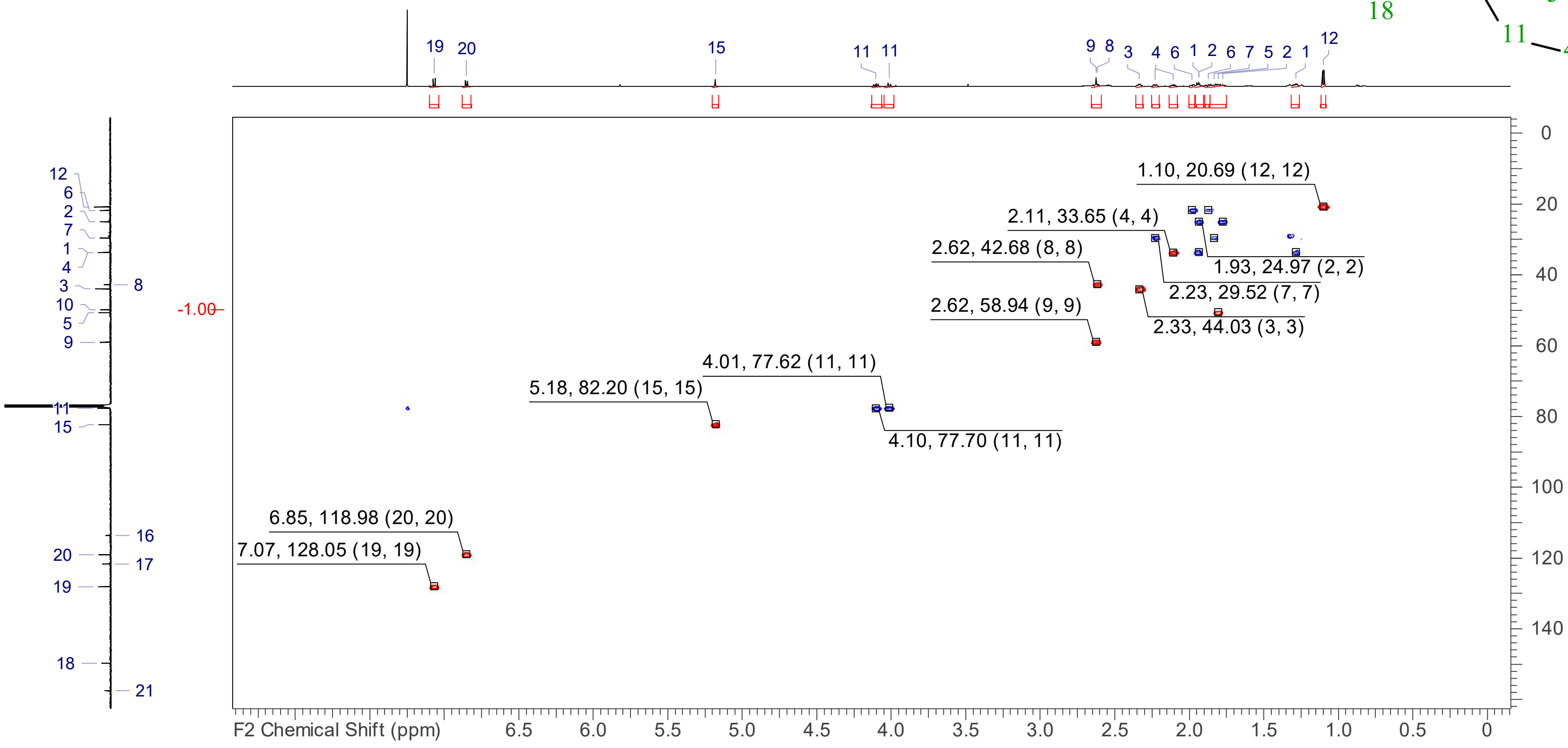

HMBC NMR spectrum (700 MHz, Chloroform-*d*) of 14-Oxo-leucopleurotinic acid (8).

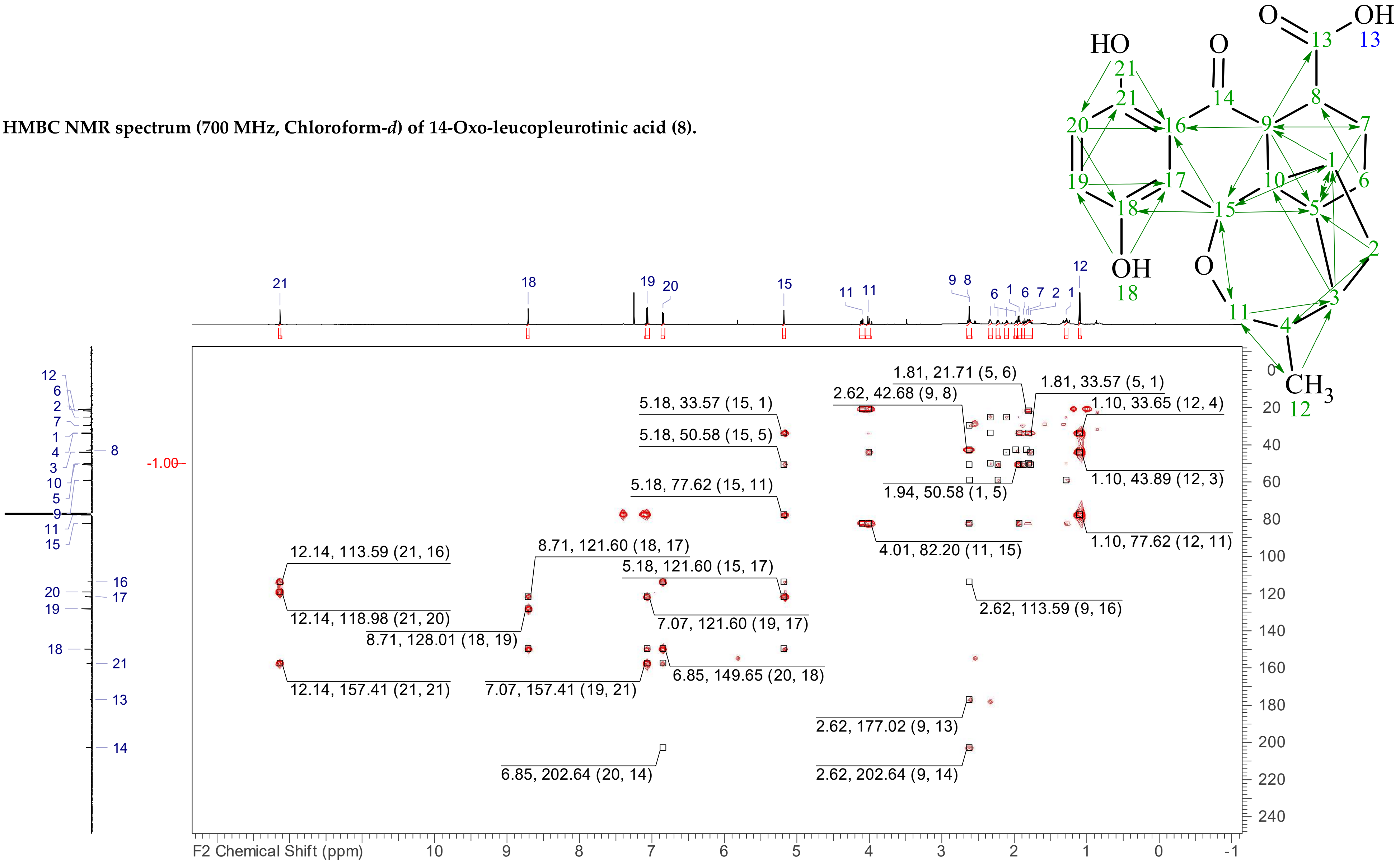

¹H, ¹H ROESY NMR spectrum (700 MHz, Chloroform-*d*) of 14-Oxo-leucopleurotinic acid (8).

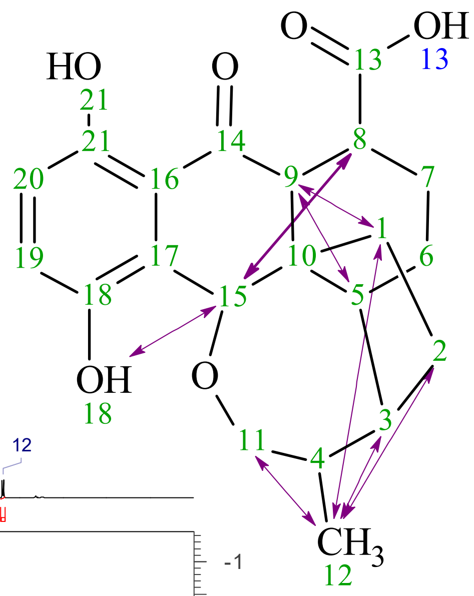

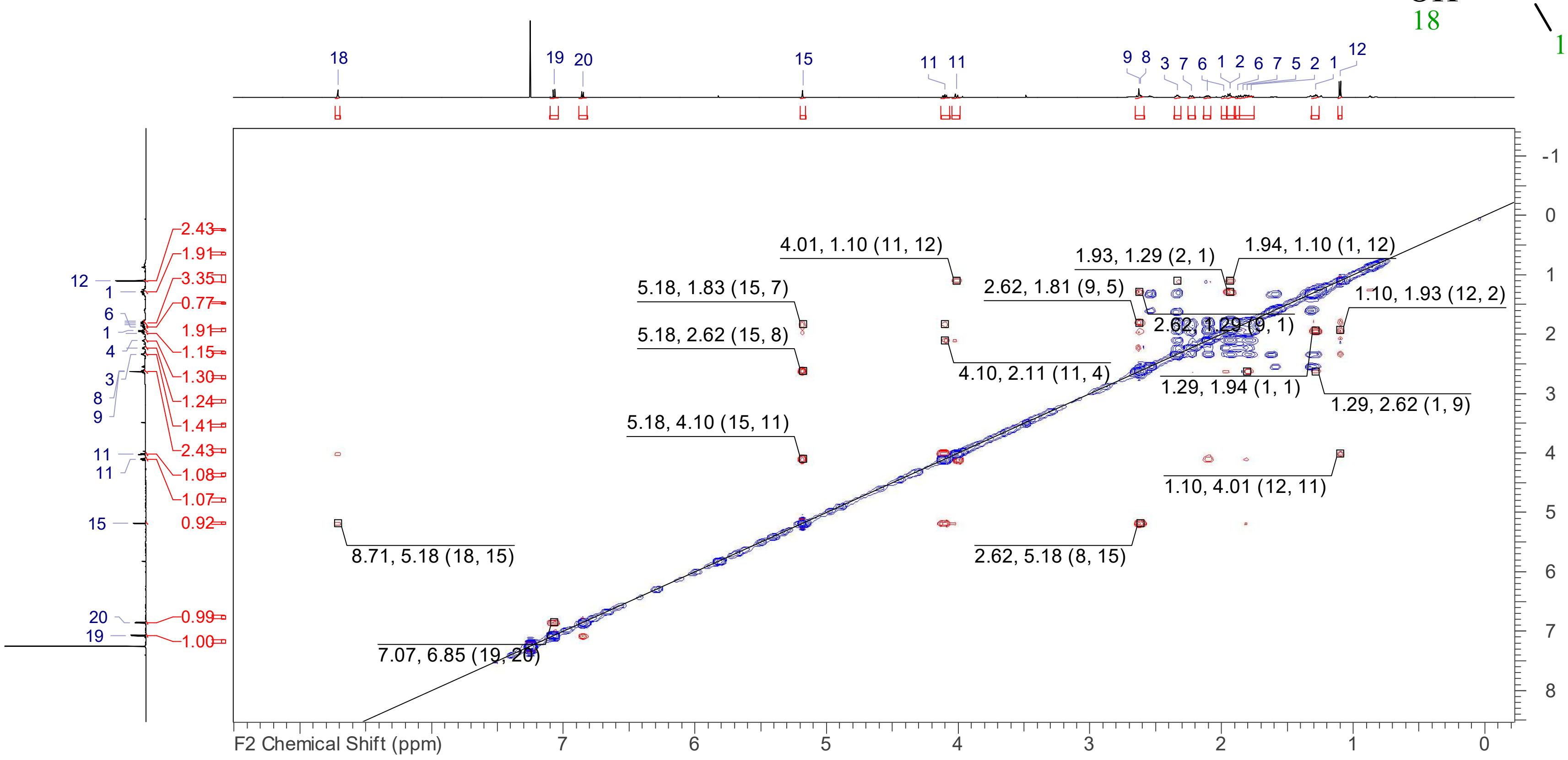

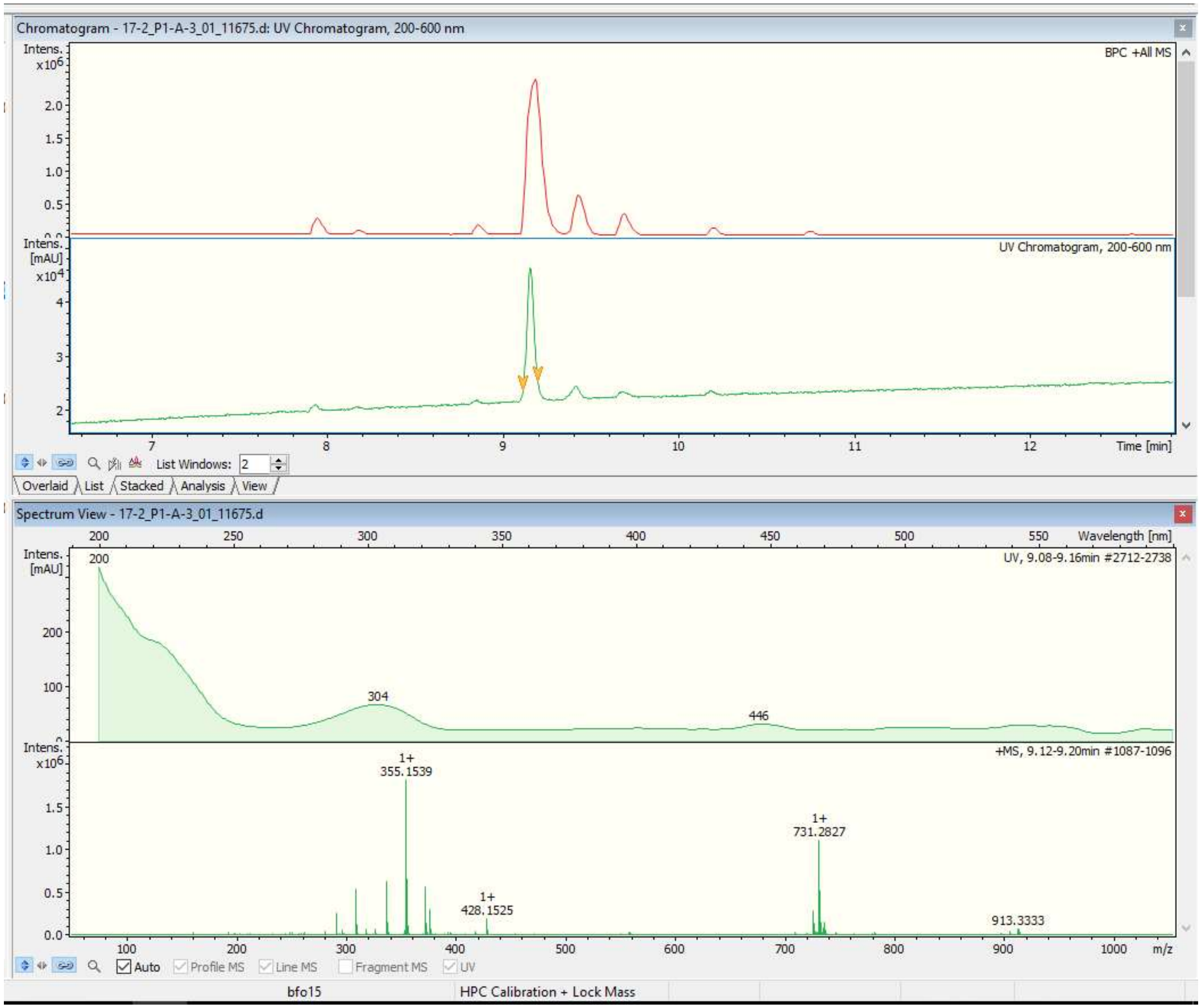

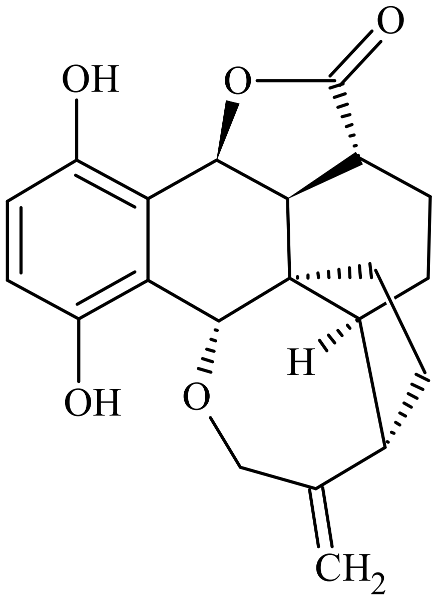

HRESIMS data of Nematoctone (9).

^{1}H NMR spectrum (700 MHz, Chloroform-*d*) of Nematoctone (9).

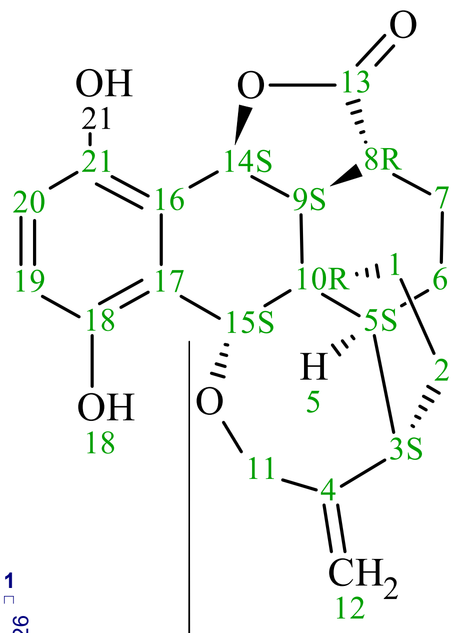

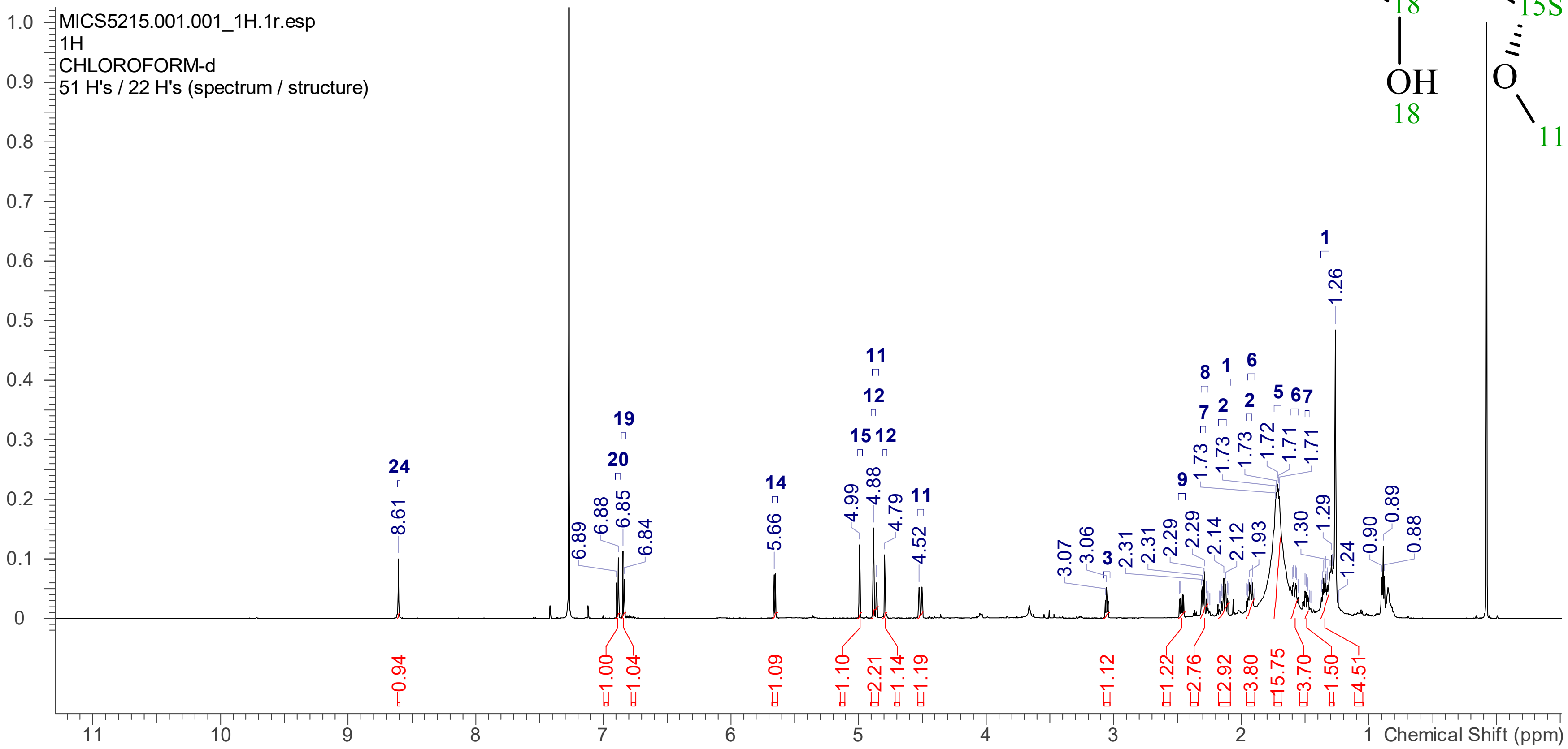

^{13}C NMR spectrum (176 MHz, Chloroform-*d*) of Nematoctone (9).

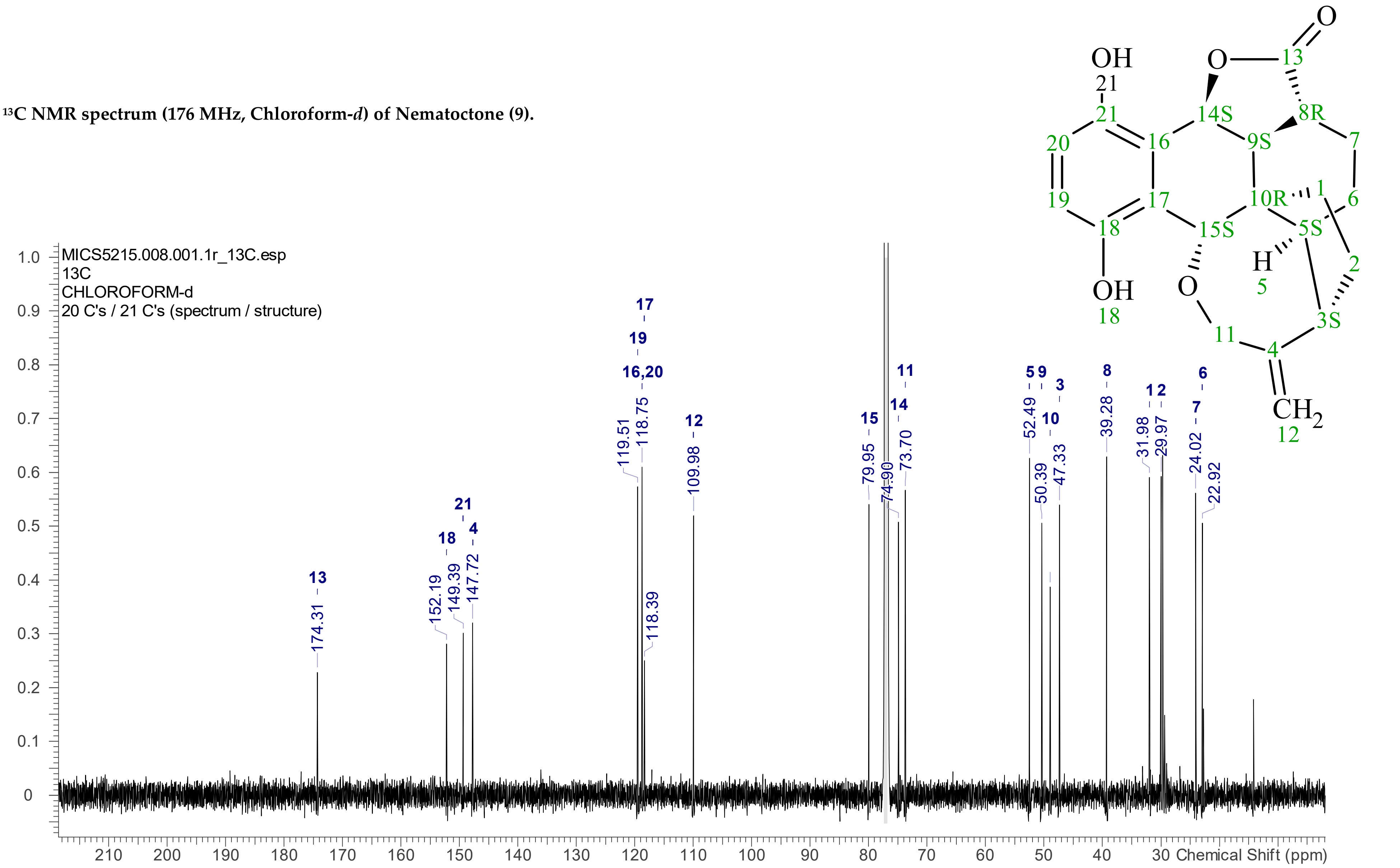

^1H, ^1H COSY NMR spectrum (700 MHz, Chloroform-*d*) of Nematoctone (9).

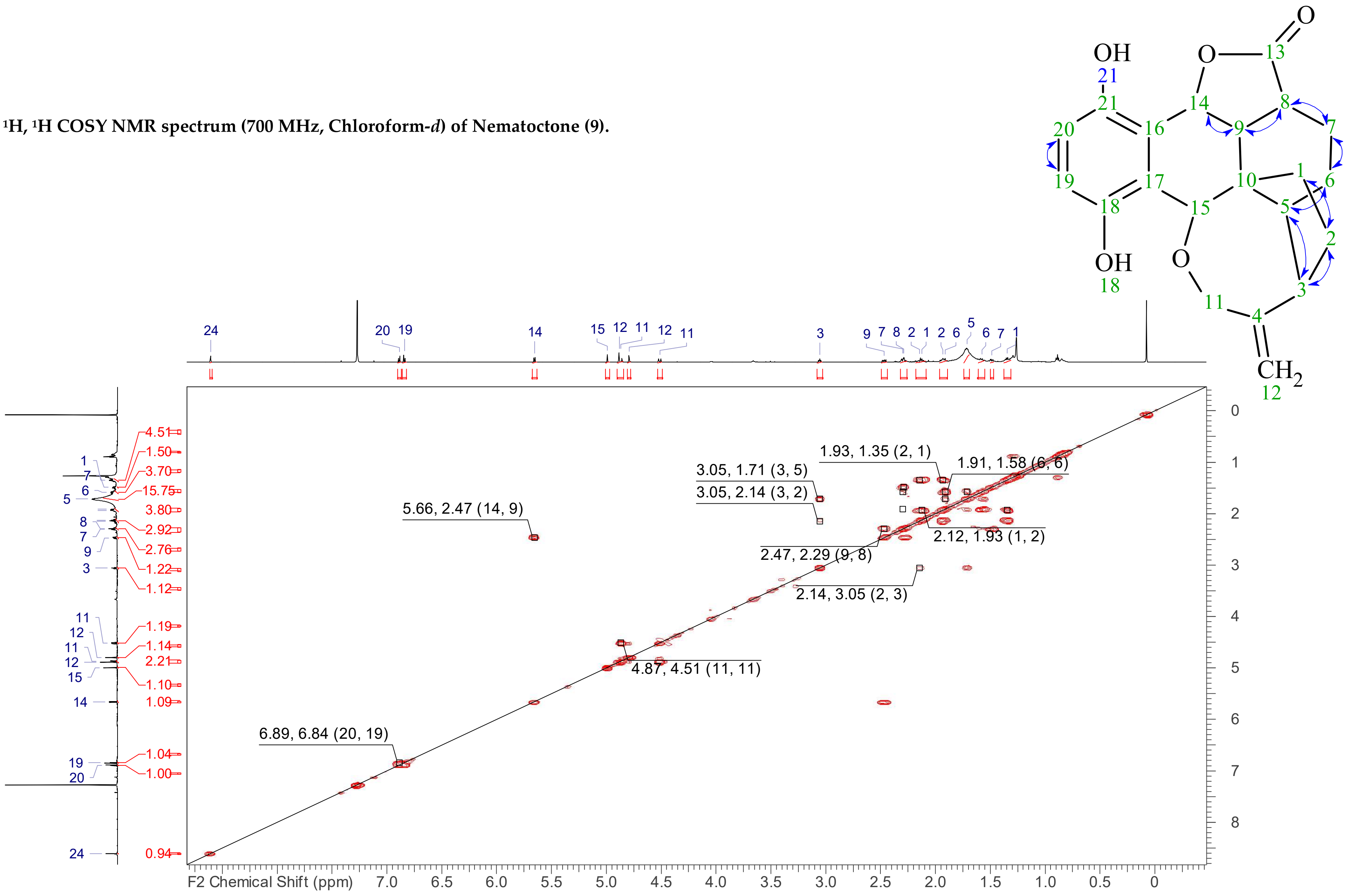

HSQC NMR spectrum (700 MHz, Chloroform-*d*) of Nematoctone (9).

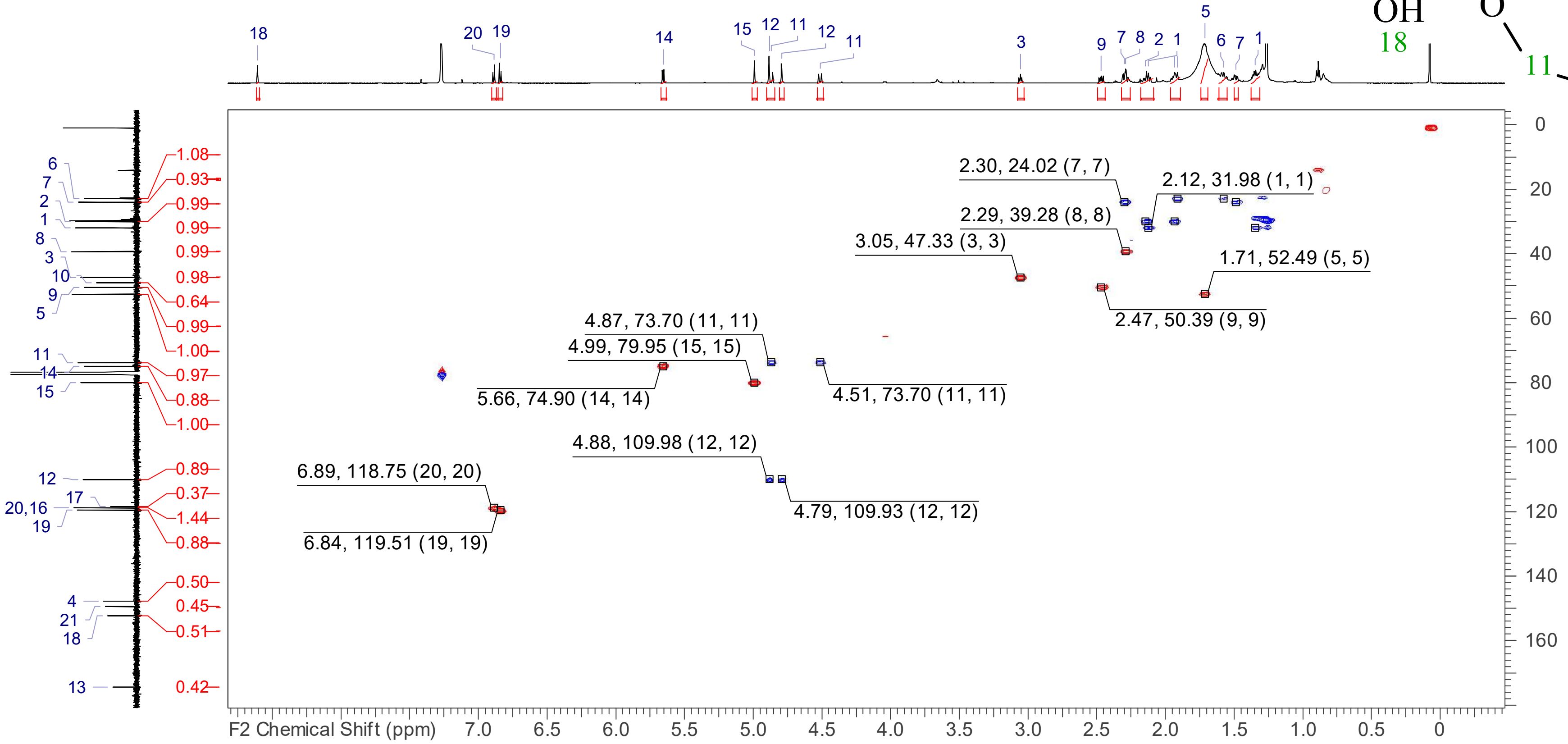

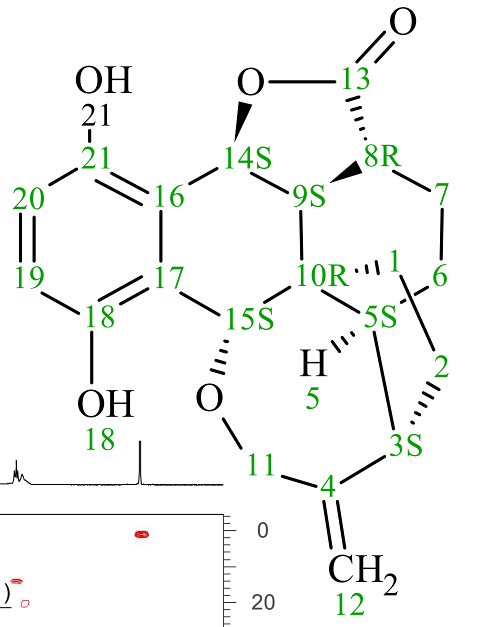

HMBC NMR spectrum (700 MHz, Chloroform-*d*) of Nematoctone (9).

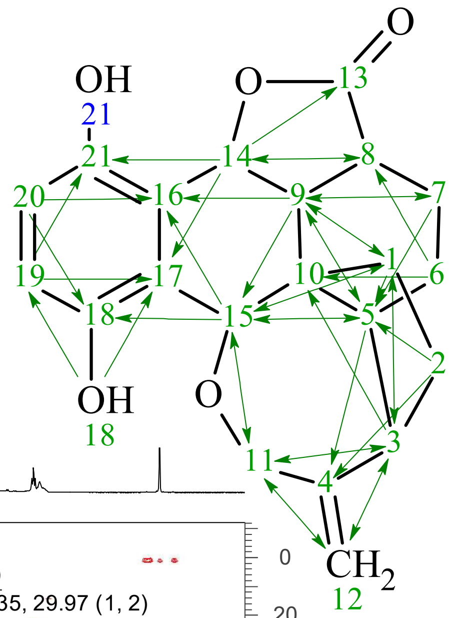

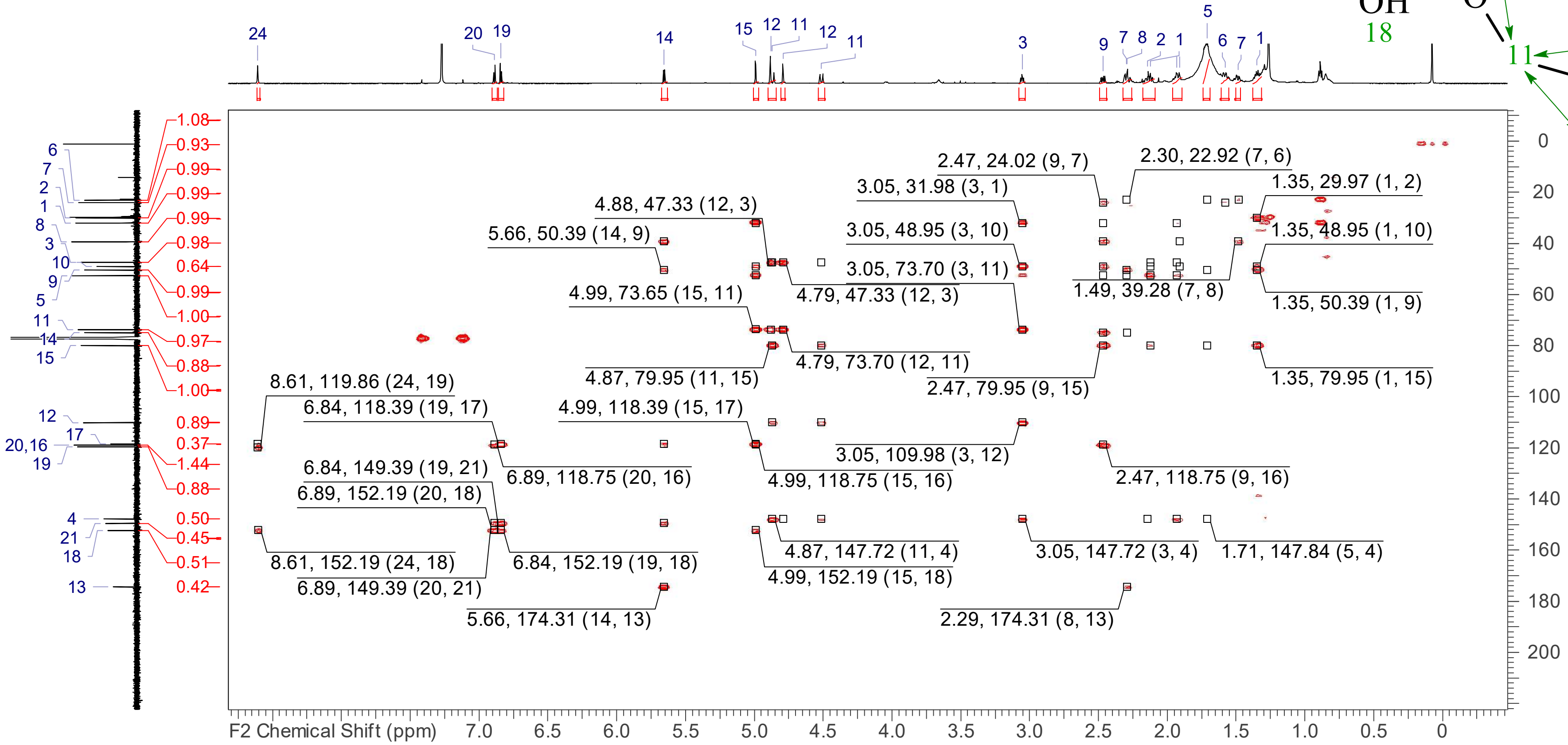

¹H, ¹H ROESY NMR spectrum (700 MHz, Chloroform-*d*) of Nematoctone (9).

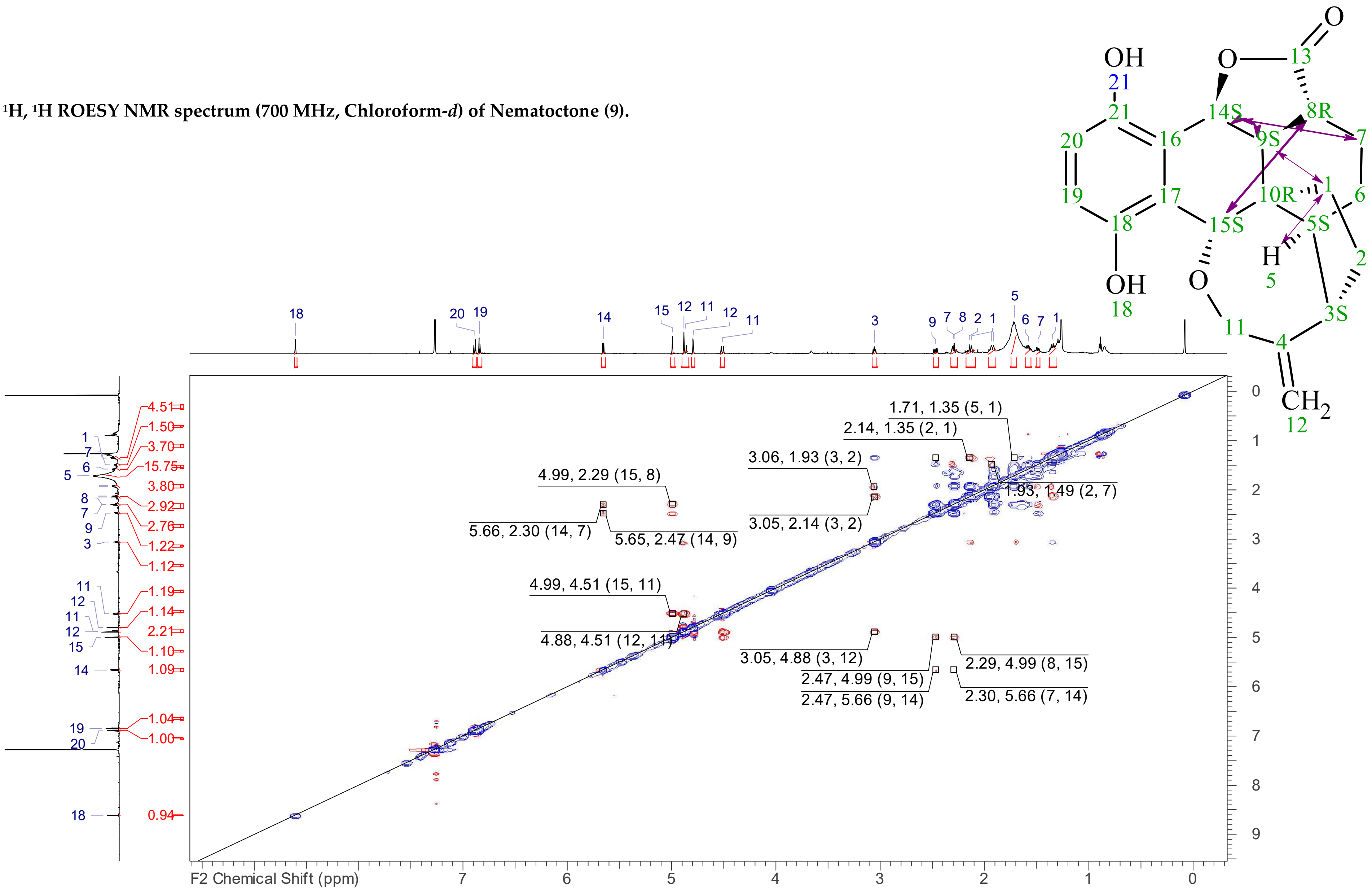

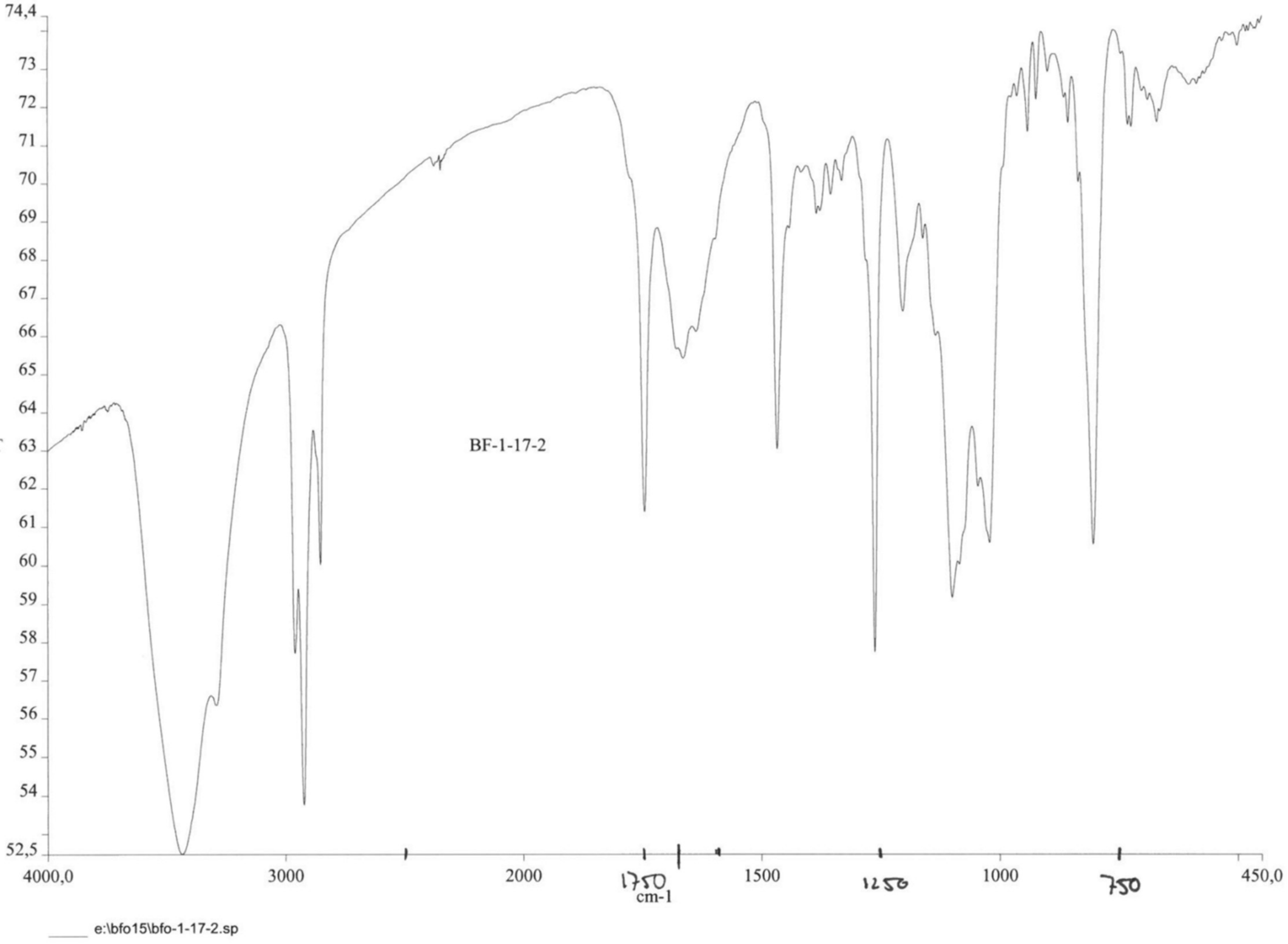
74,4
73
72
71
70
69
68
67
66
65
64
63
62
61
60
59
58
57
56
55
54
52,5
BF-1-17-2
4000,0
3000
2000
1750
1500
1250
1000
750
450,0
cm-1
e:\bfo15\bfo-1-17-2.sp

Figure S4: IR spectrum (KBr) of Nematoctone (9).

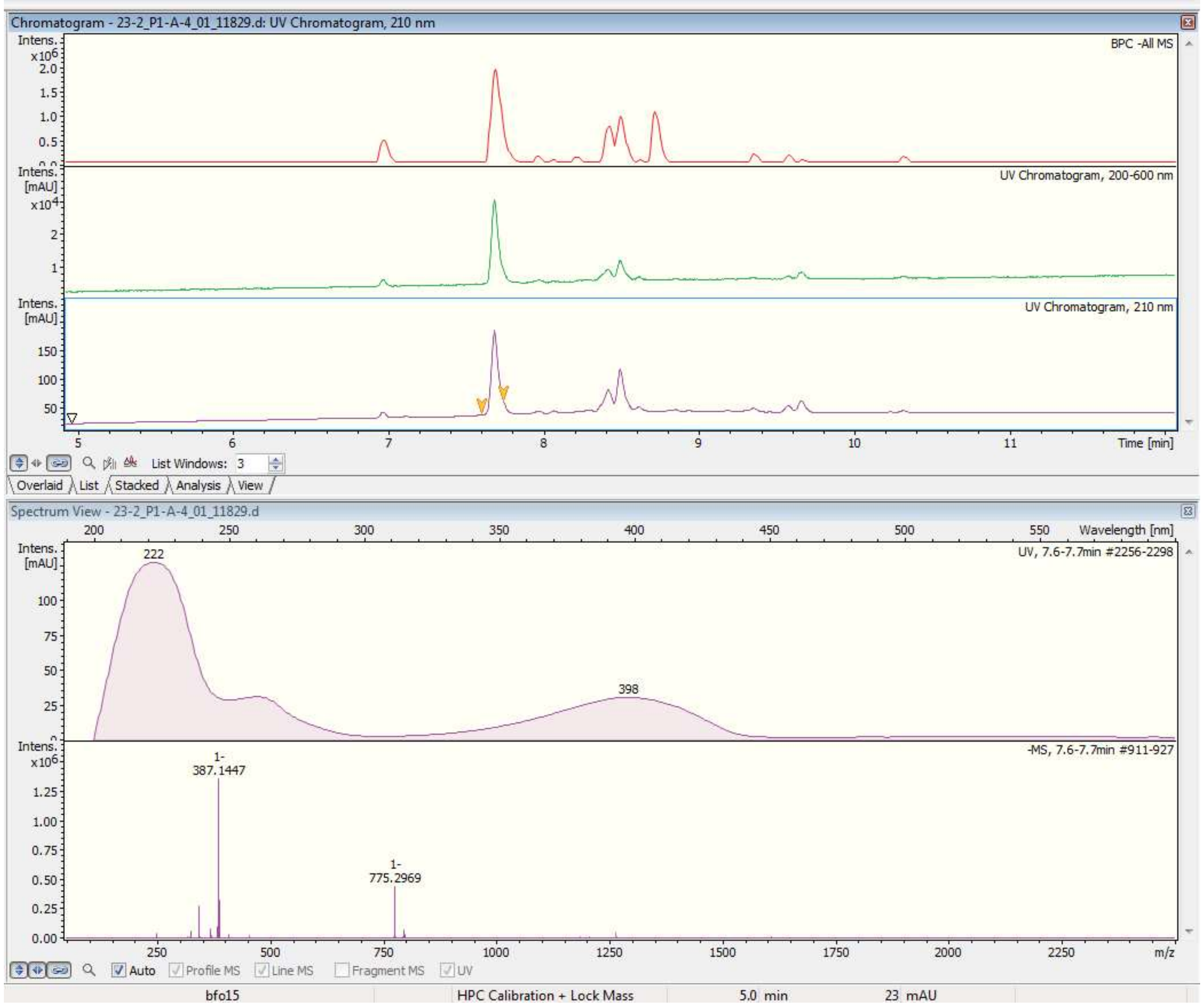

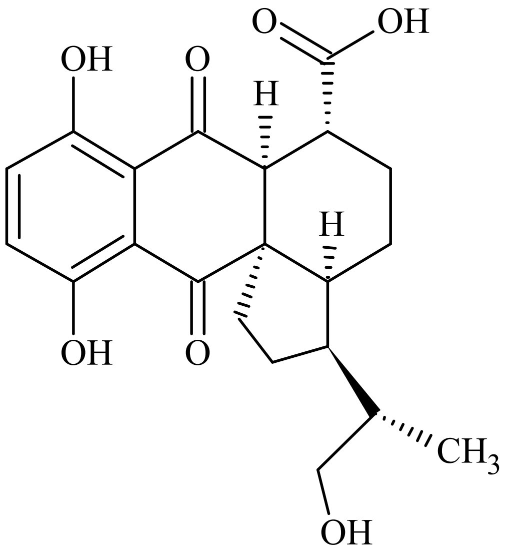

HRESIMS data of Di-oxo-leucopleurotinic acid (10).

¹H NMR spectrum (700 MHz, Chloroform-*d*) of Di-oxo-leucopleurotinic acid (10).

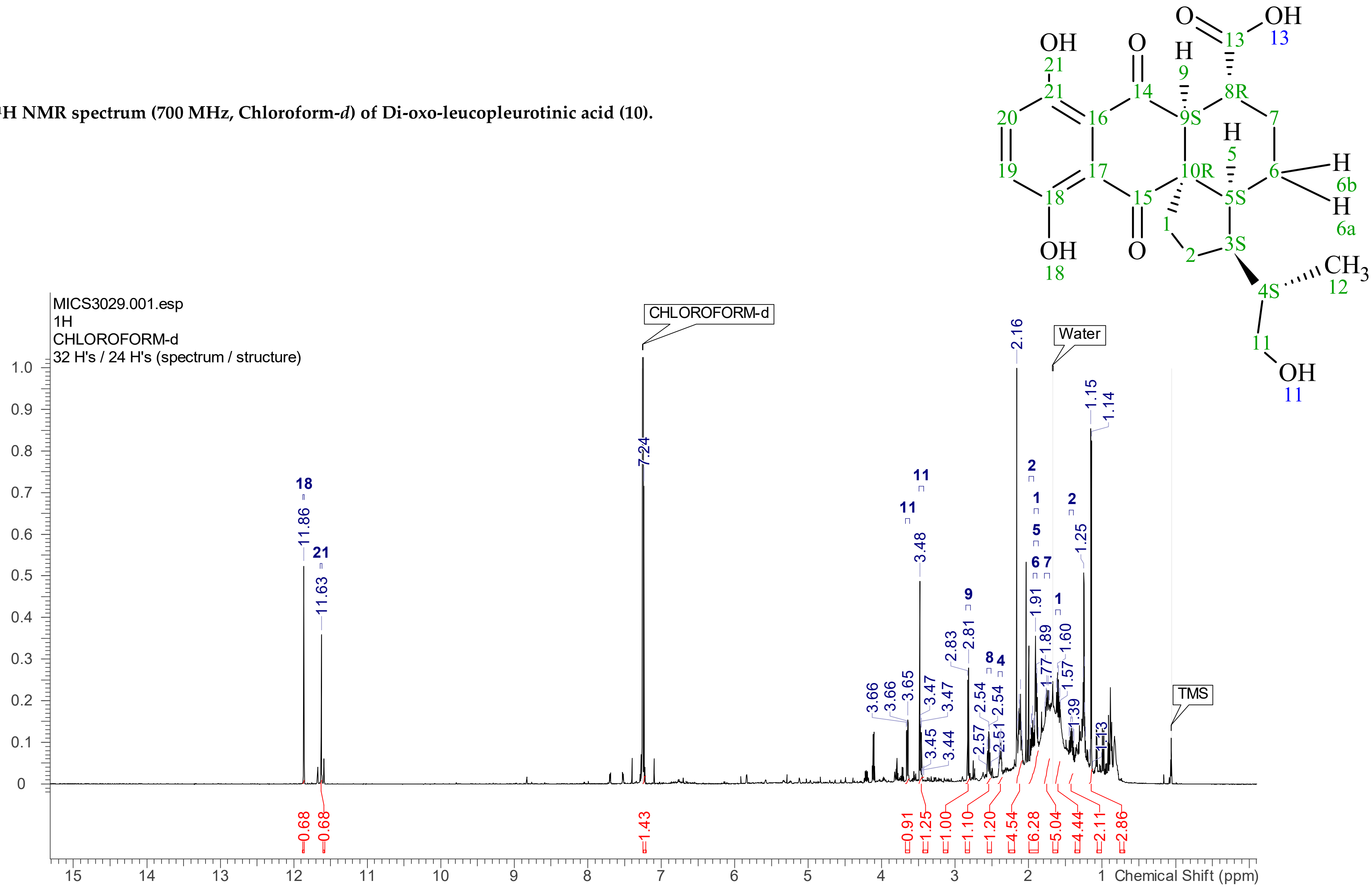

^{13}C NMR spectrum (176 MHz, Chloroform-*d*) of Di-oxo-leucopleurotinic acid (10).

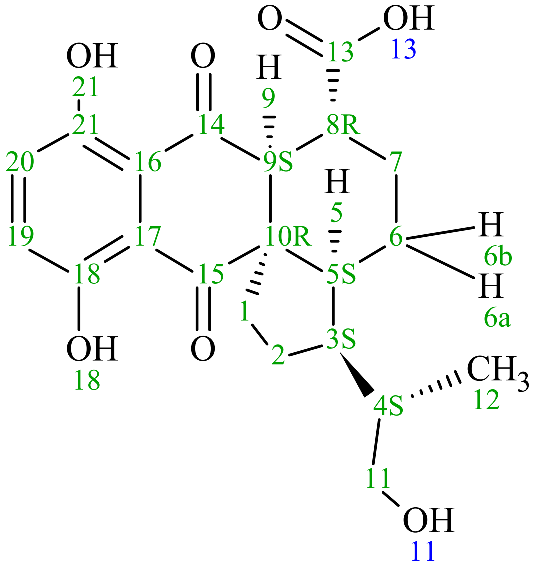

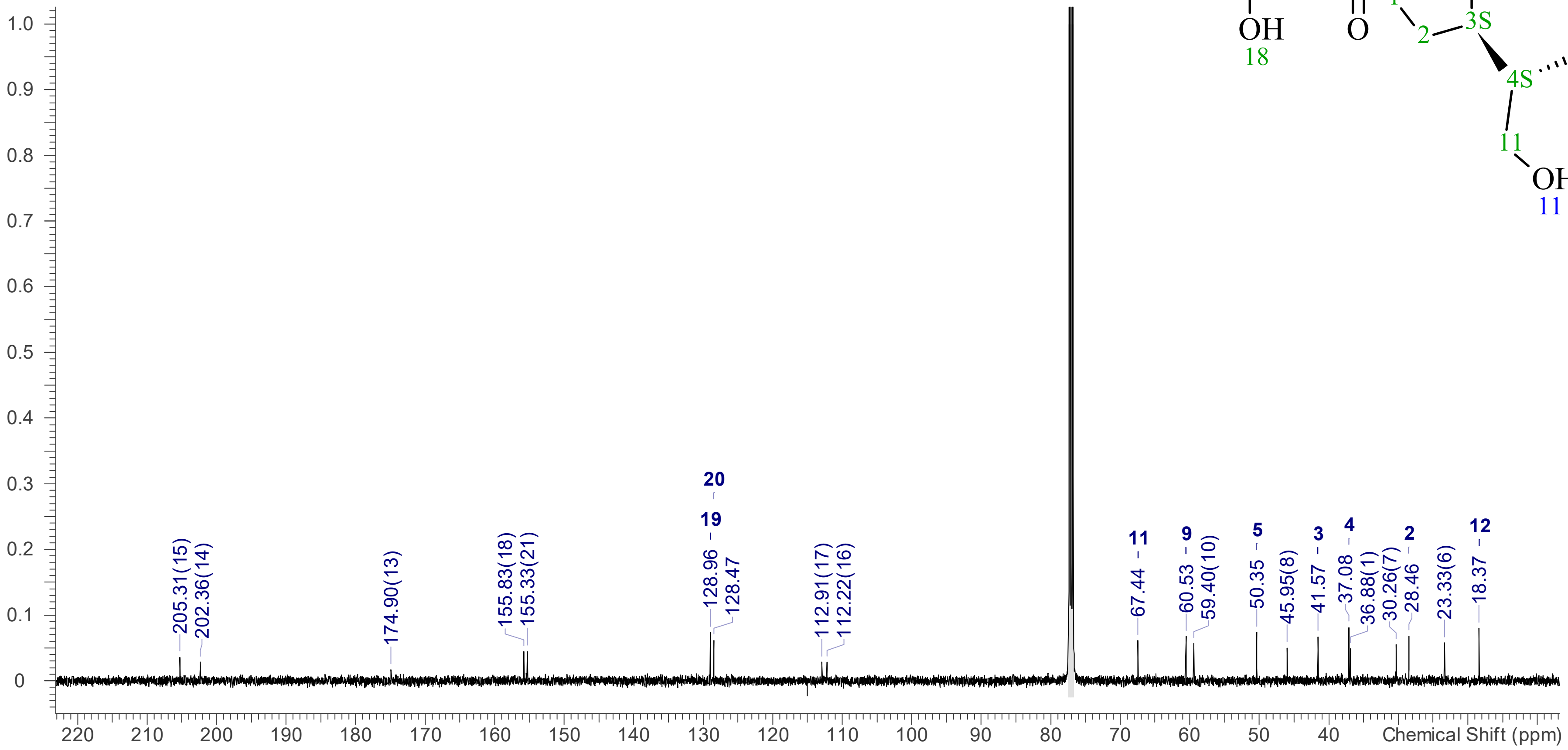

¹H, ¹H COSY NMR spectrum (700 MHz, Chloroform-*d*) of Di-oxo-leucopleurotinic acid (10).

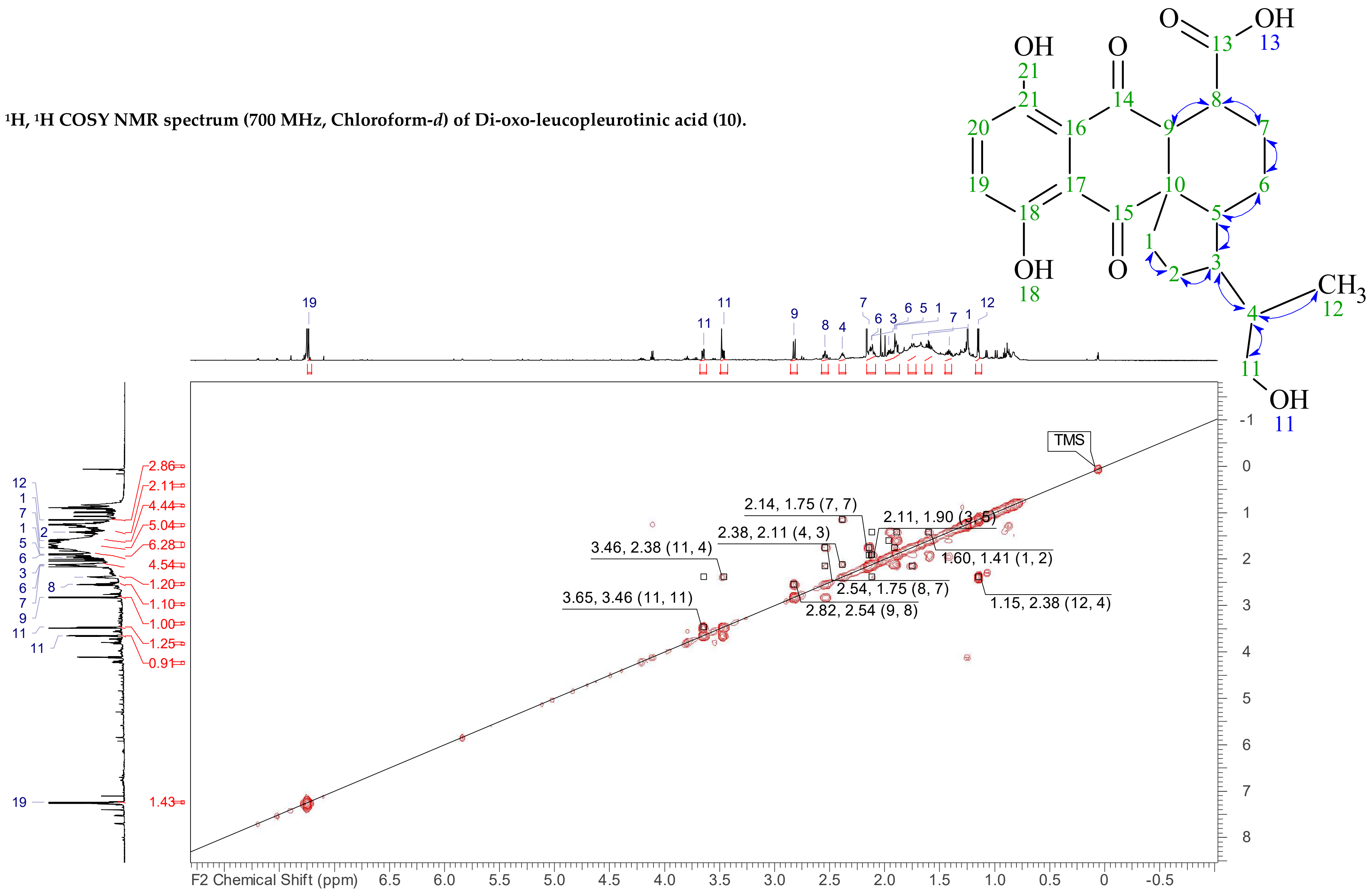

HSQC NMR spectrum (700 MHz, Chloroform-*d*) of Di-oxo-leucopleurotinic acid (10).

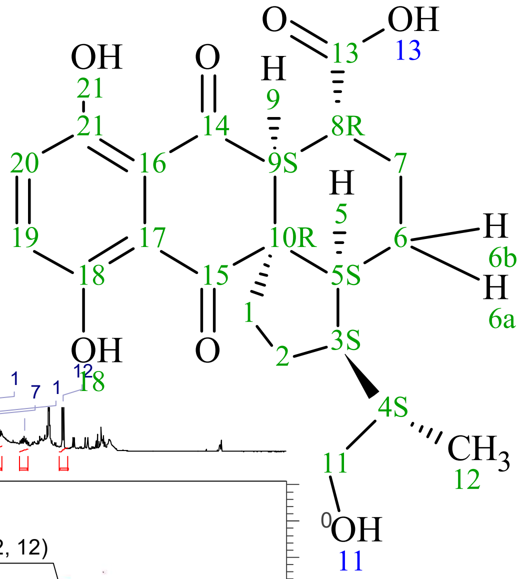

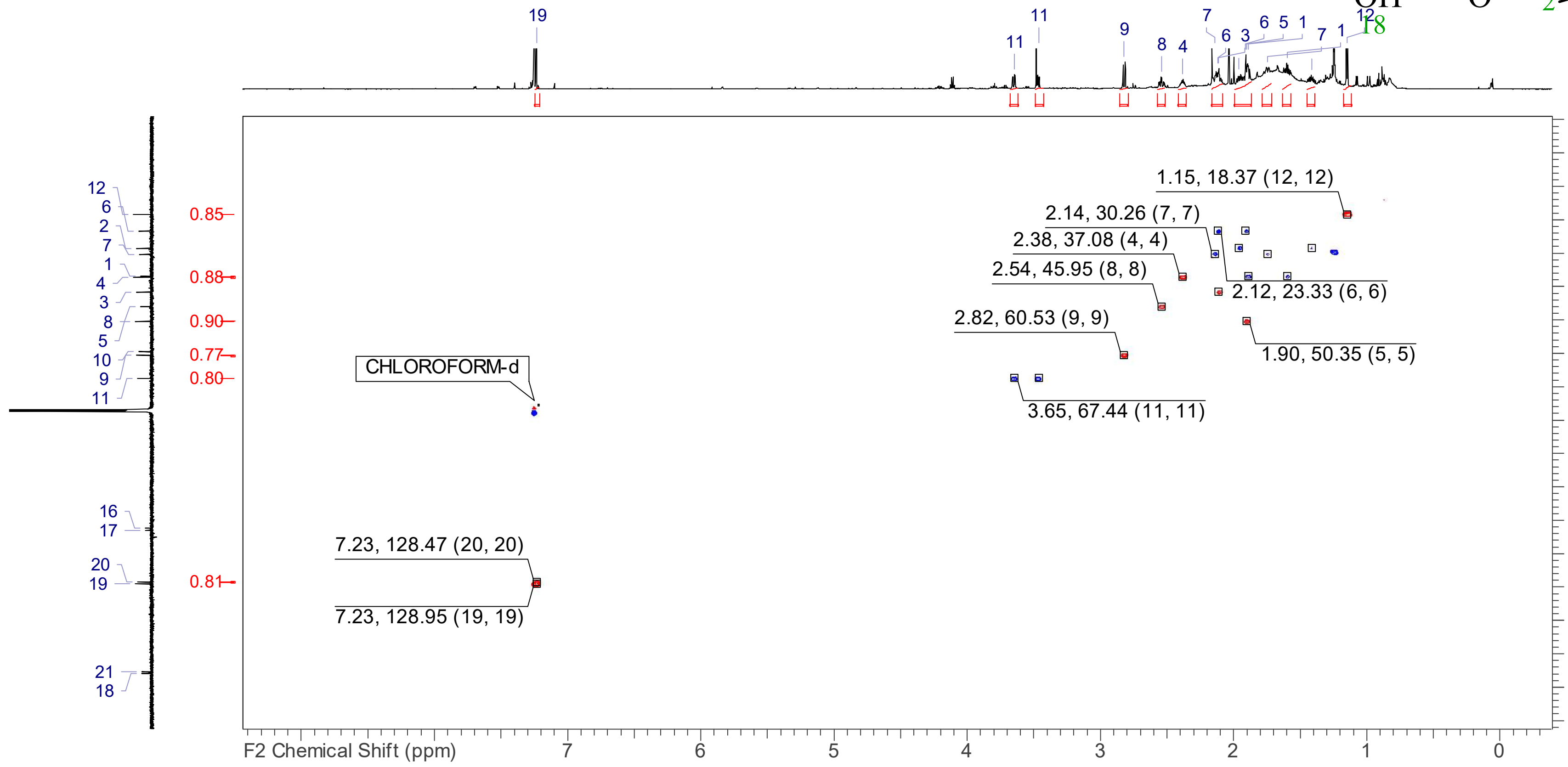

HMBC NMR spectrum (700 MHz, Chloroform-*d*) of Di-oxo-leucopleurotinic acid (10).

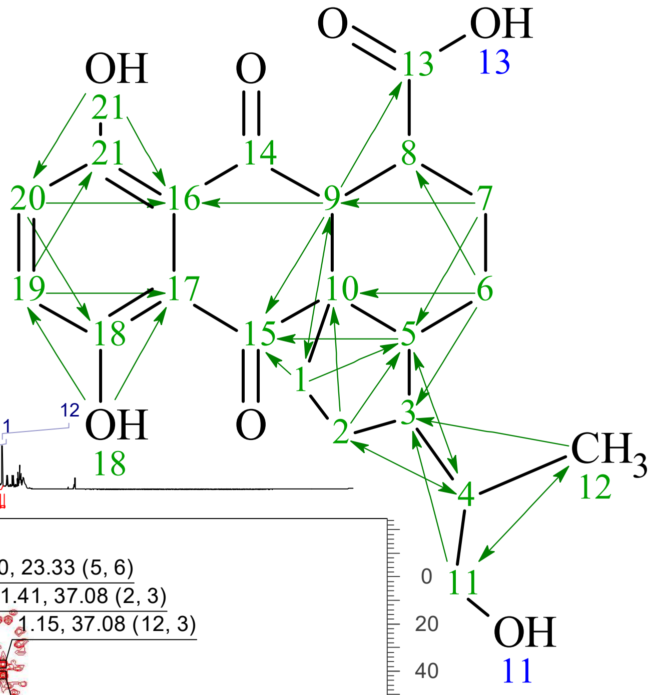

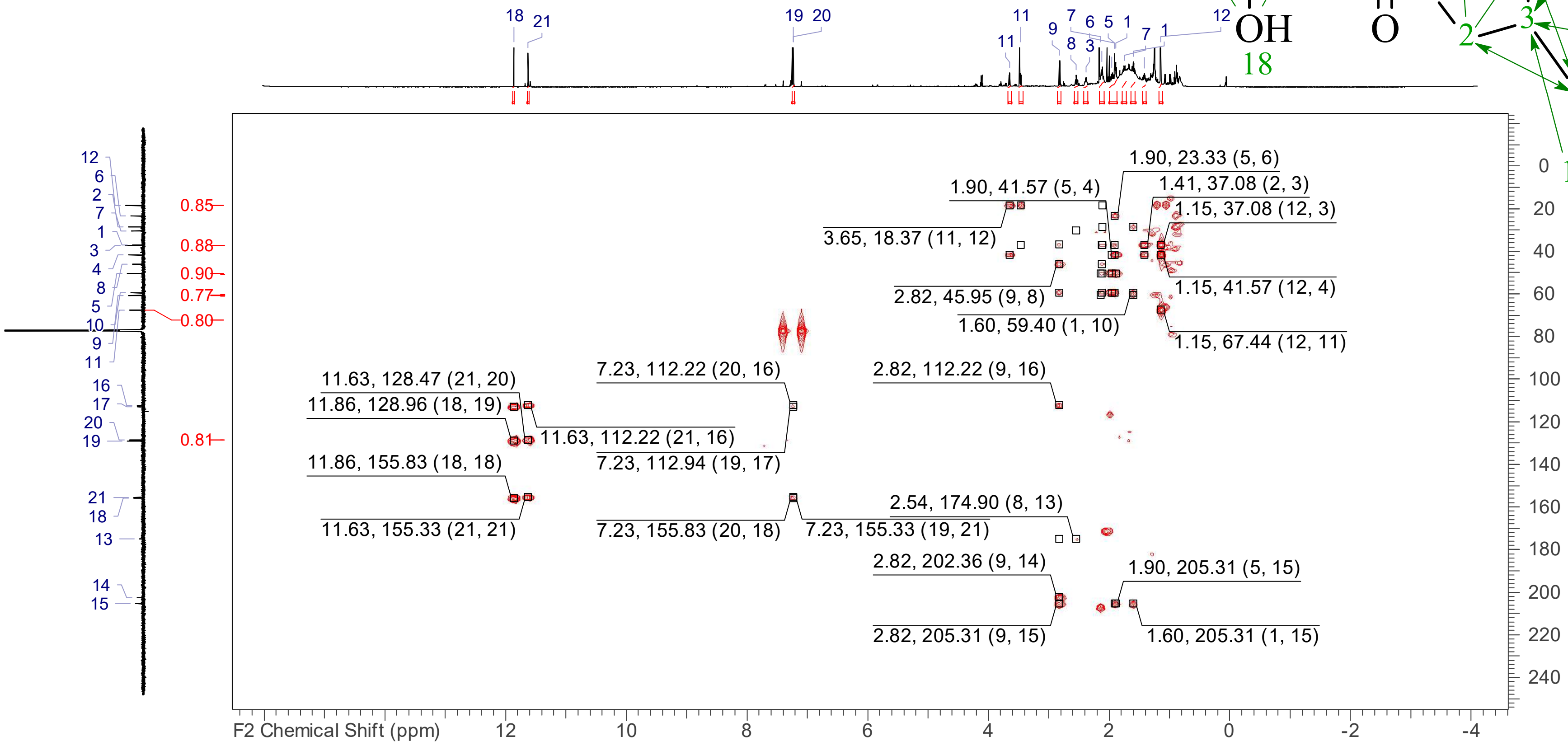

^{1}H, ^{1}H ROESY NMR spectrum (700 MHz, Chloroform-*d*) of Di-oxo-leucopleurotinic acid (10).

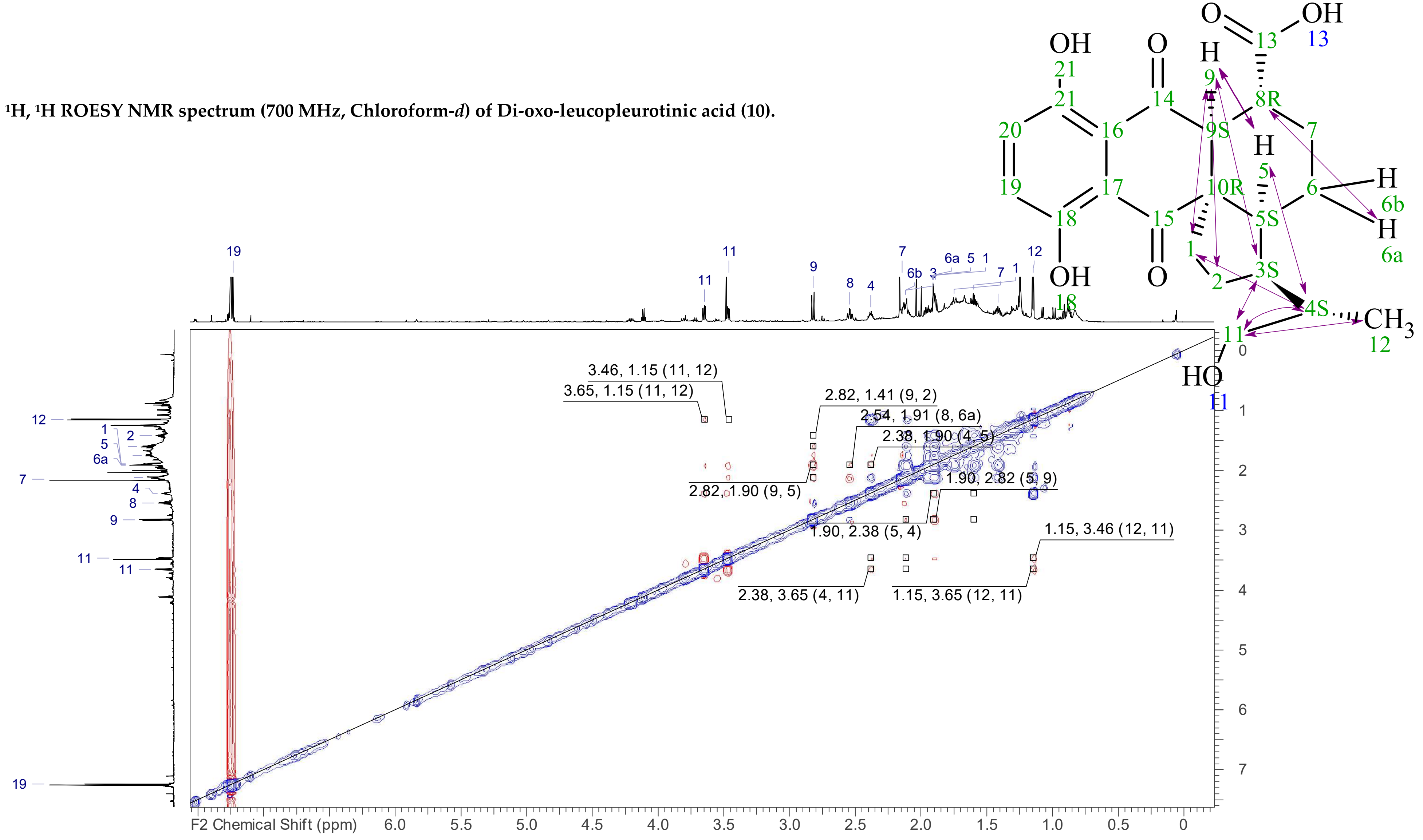

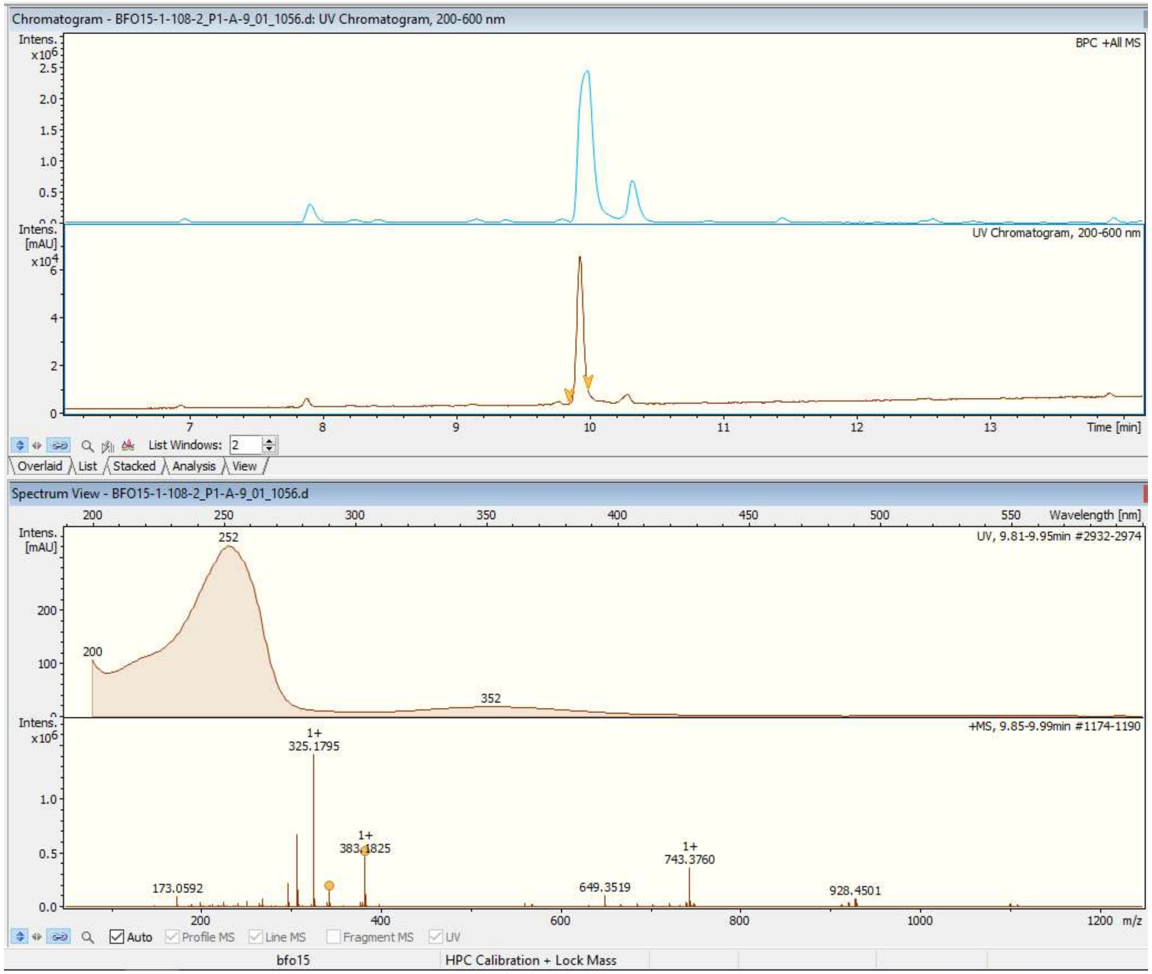

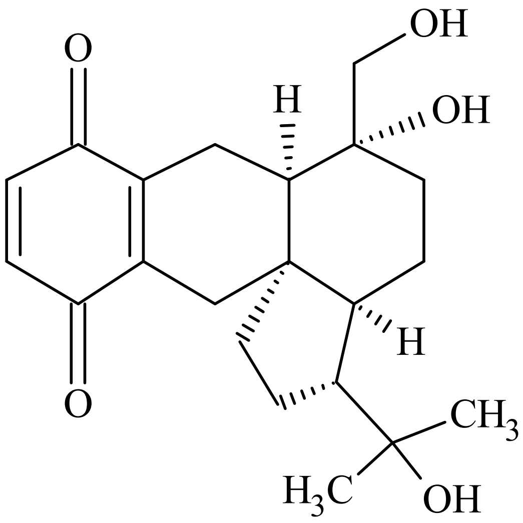

HRESIMS data of 4-Hydroxy-pleurogrisein (11).

¹H NMR (500 MHz, Acetone-*d*₆) of 4-Hydroxy-pleurogrisein (11).

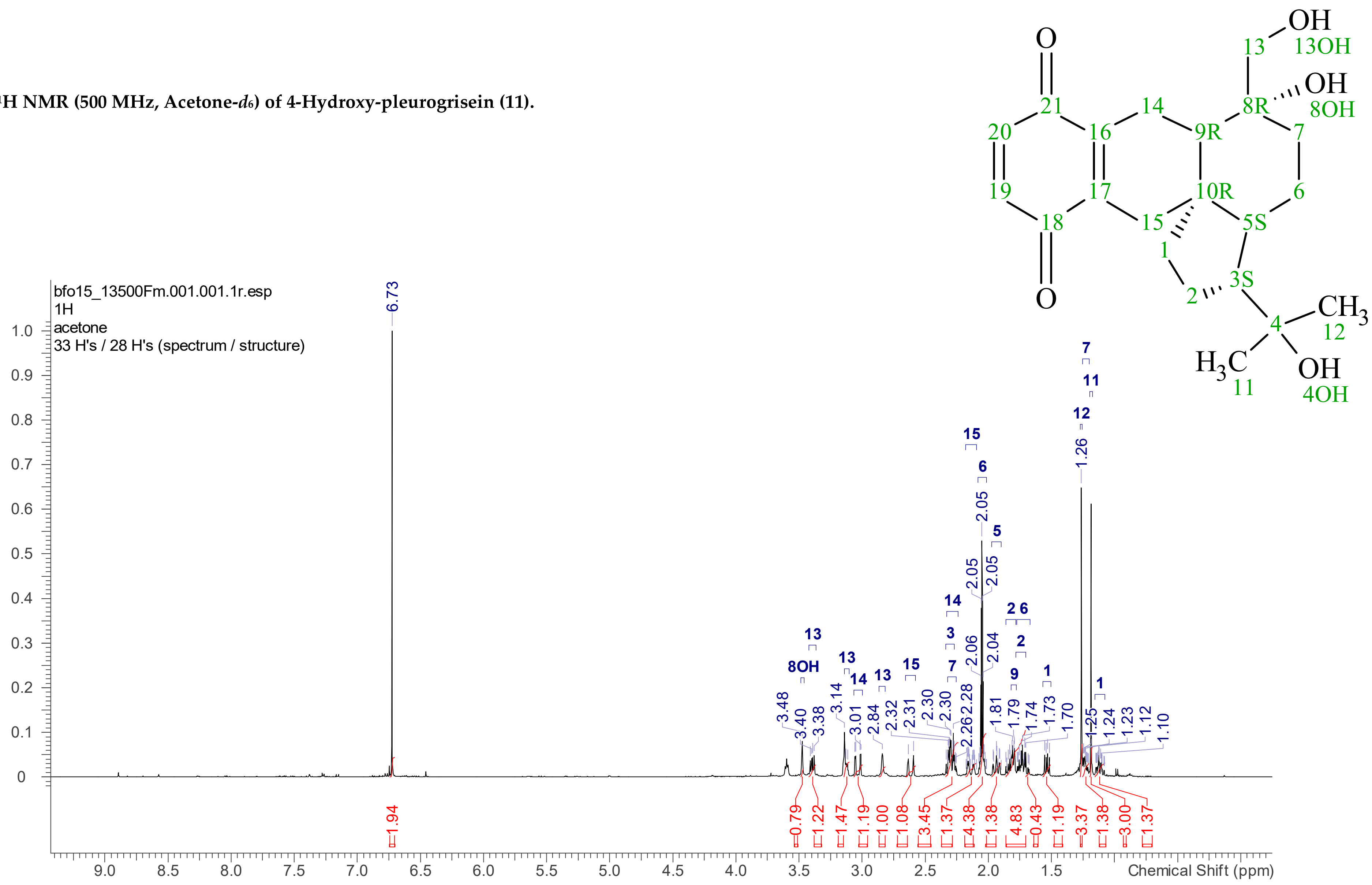

^{13}C NMR (125 MHz, Acetone-d_6) of 4-Hydroxy-pleurogrisein (11).

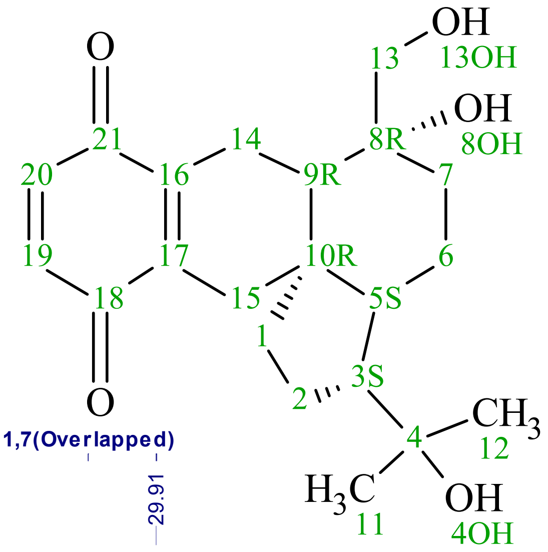

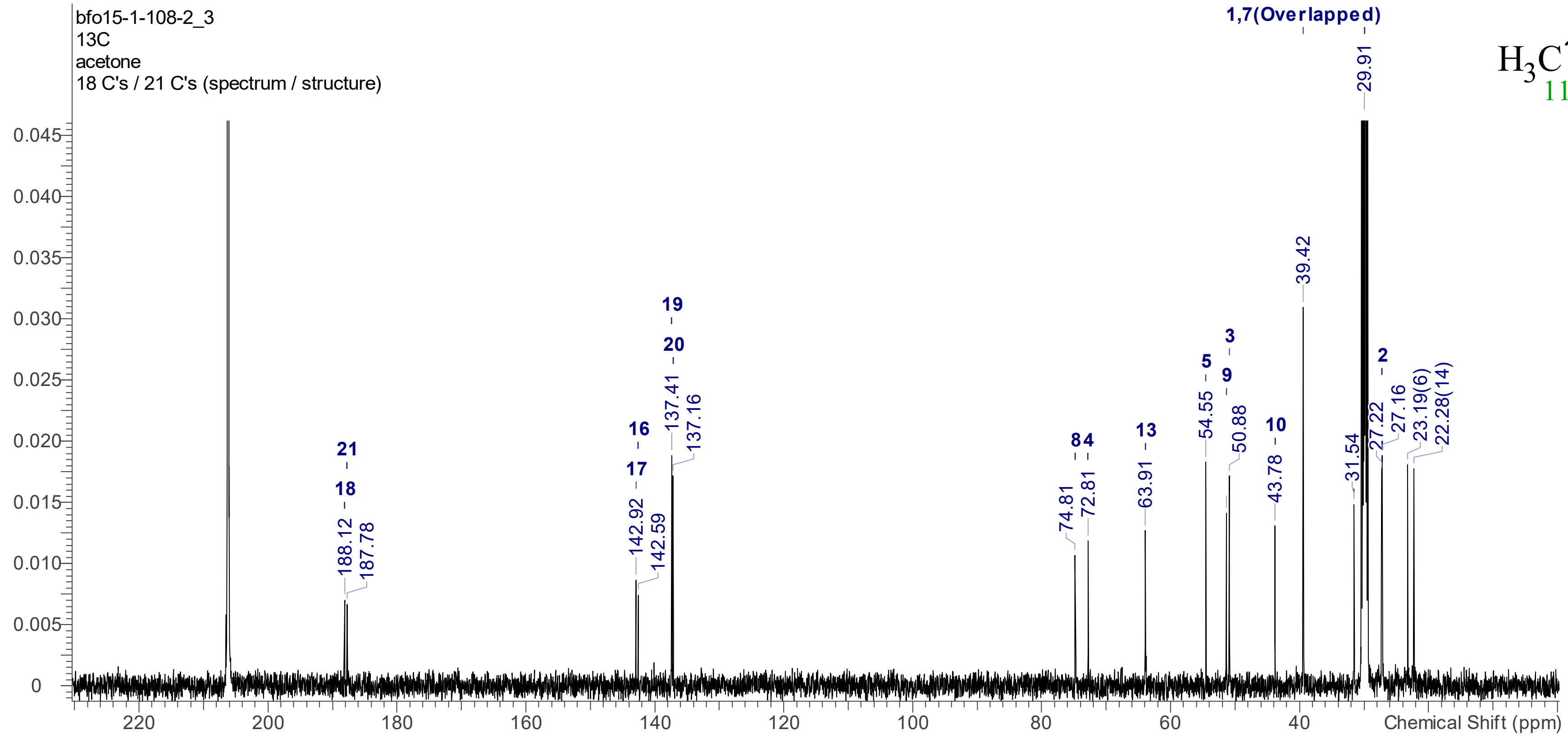

¹H, ¹H COSY NMR (500 MHz, Acetone-d_6) of 4-Hydroxy-pleurogrisein (11).

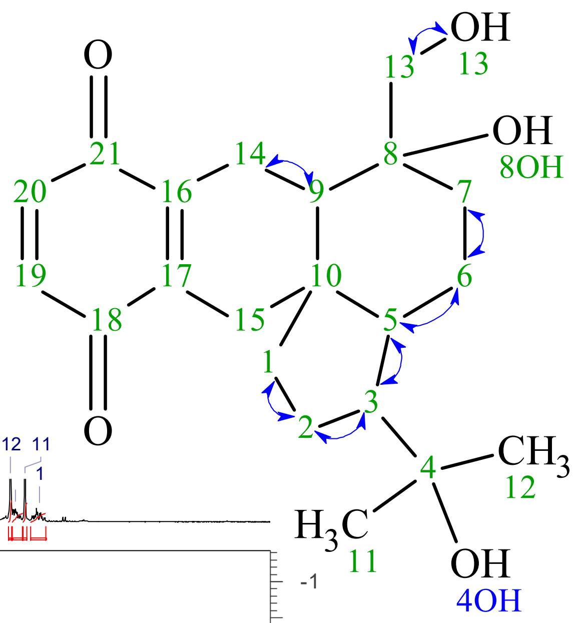

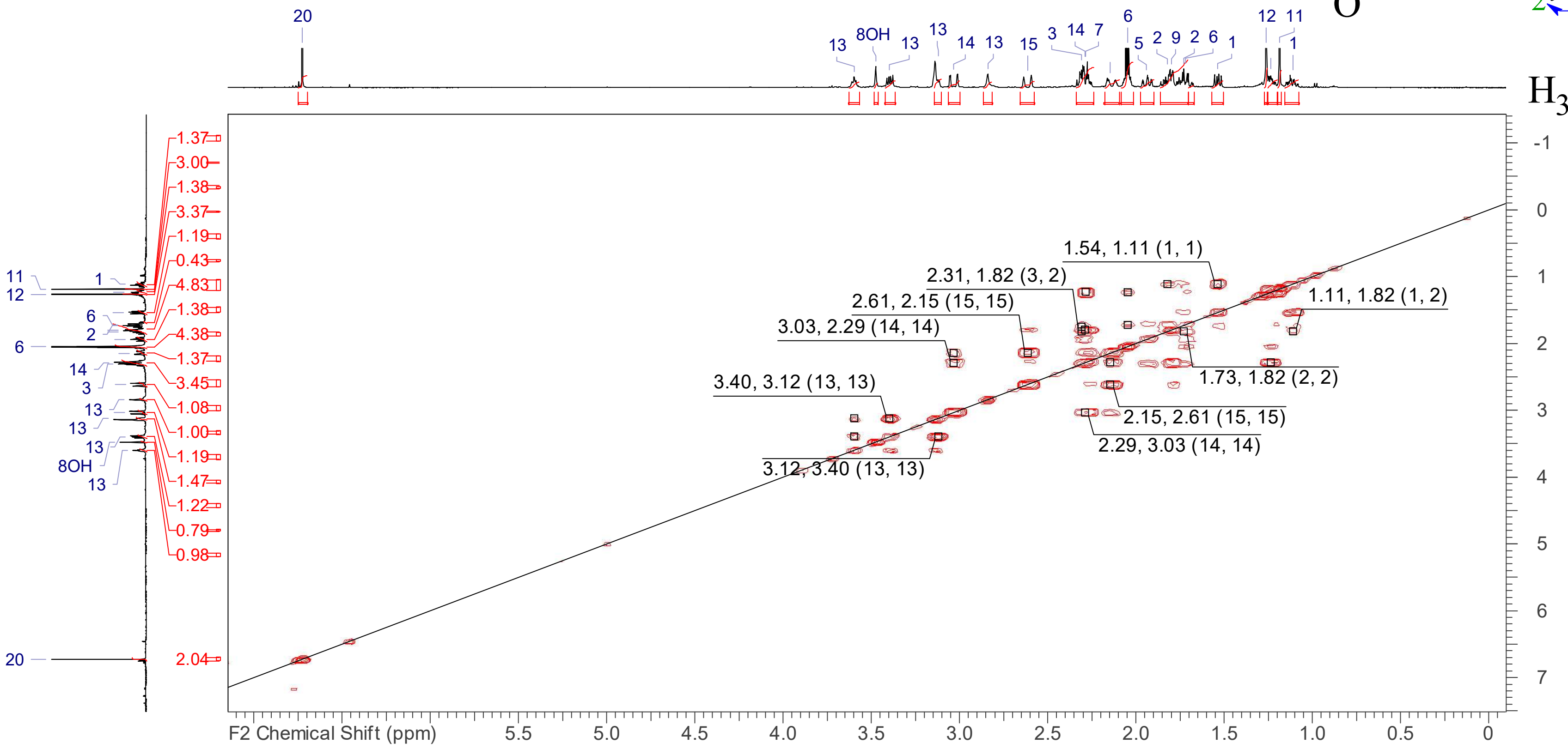

HSQC NMR (500 MHz, Acetone-*d*₆) of 4-Hydroxy-pleurogrisein (11).

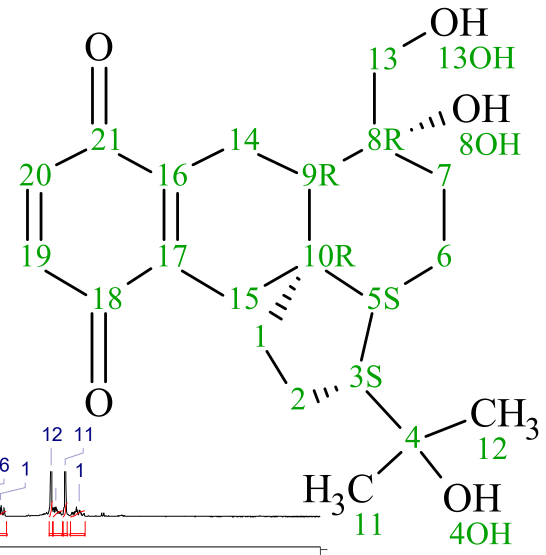

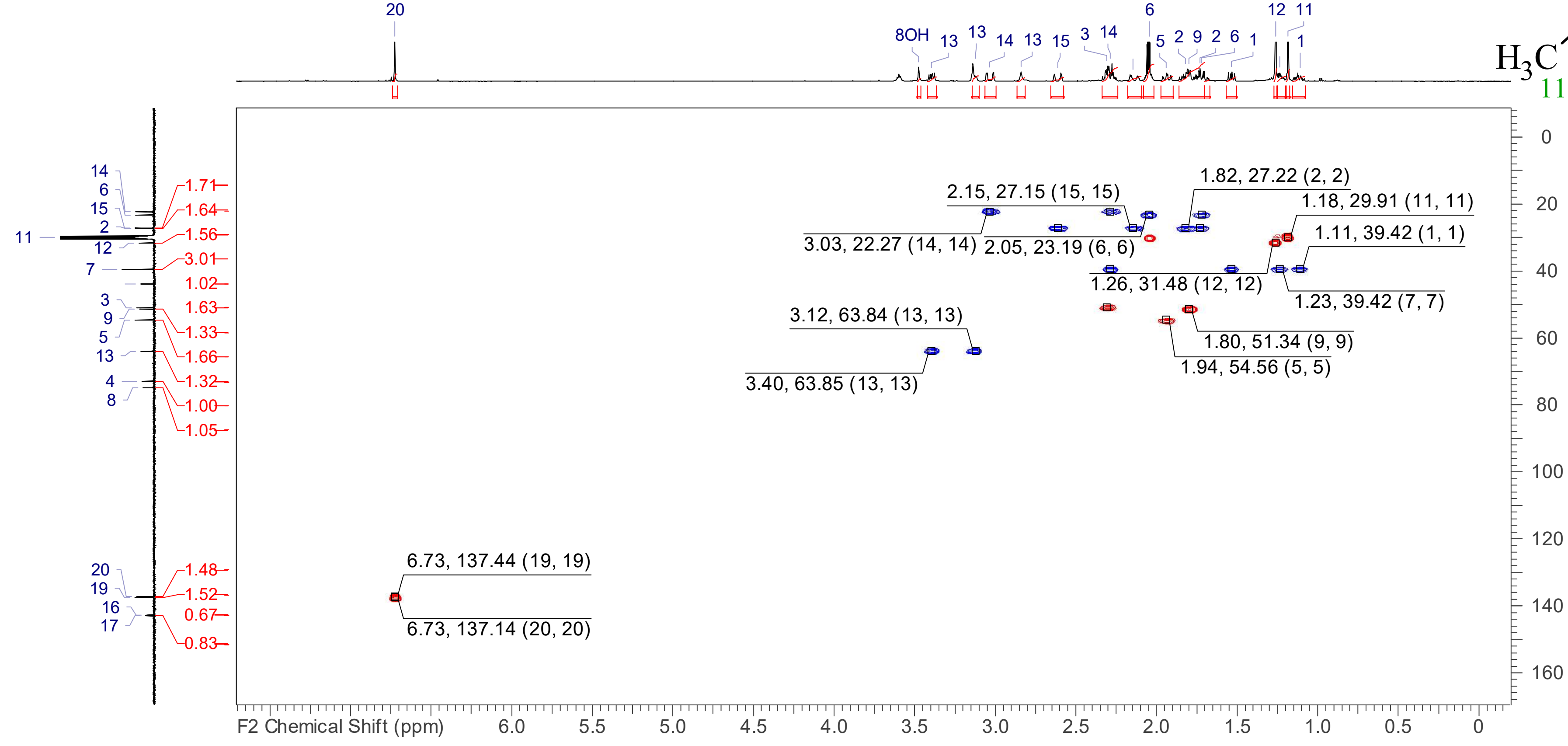

HMBC NMR (500 MHz, Acetone-d_6) of 4-Hydroxy-pleurogrisein (11).

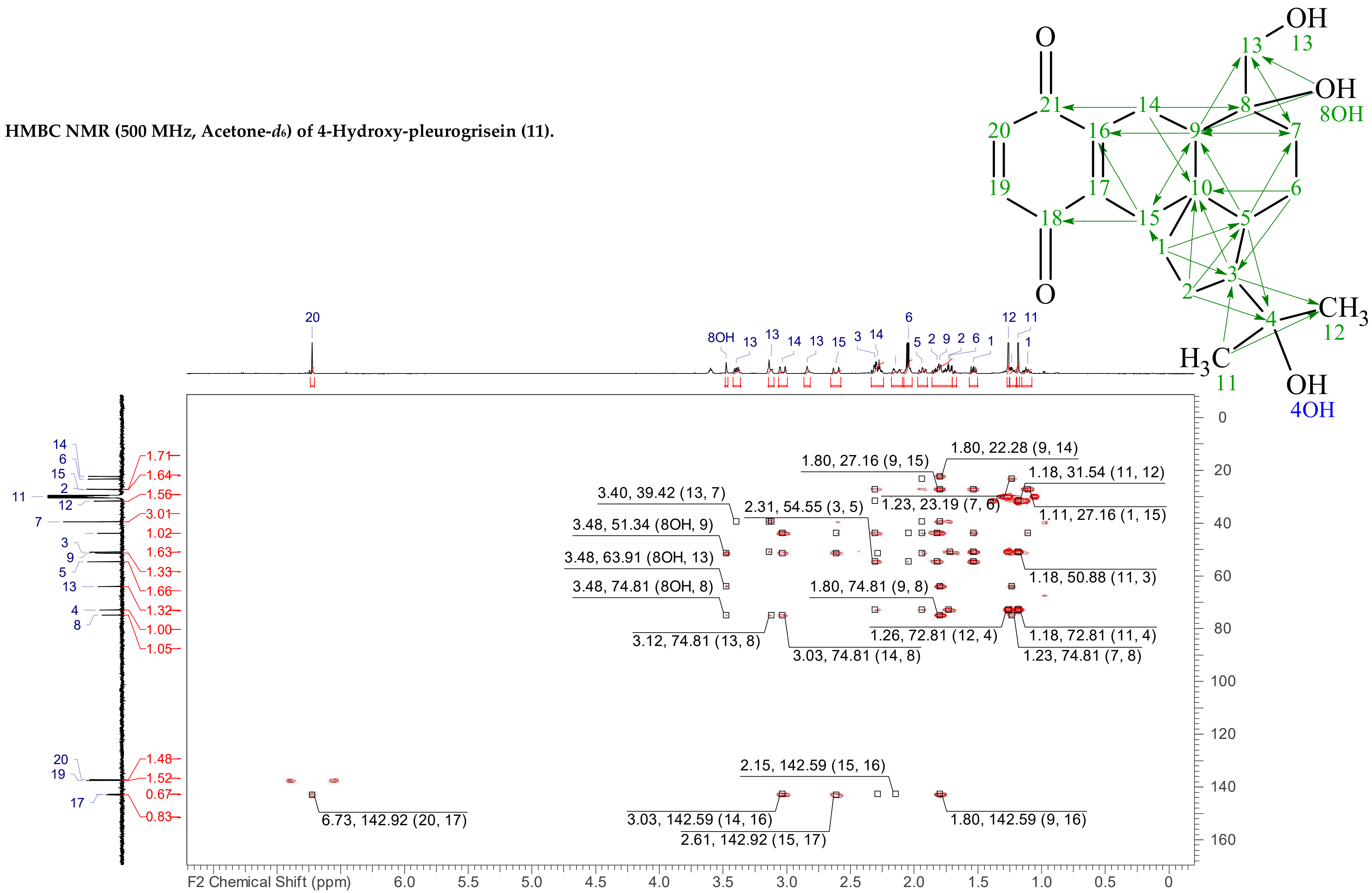

^{1}H, ^{1}H ROESY NMR (500 MHz, Acetone-d_6) of 4-Hydroxy-pleurogrisein (11).

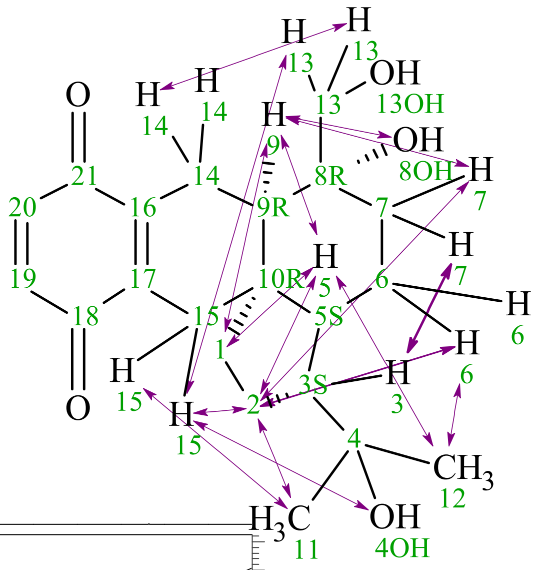

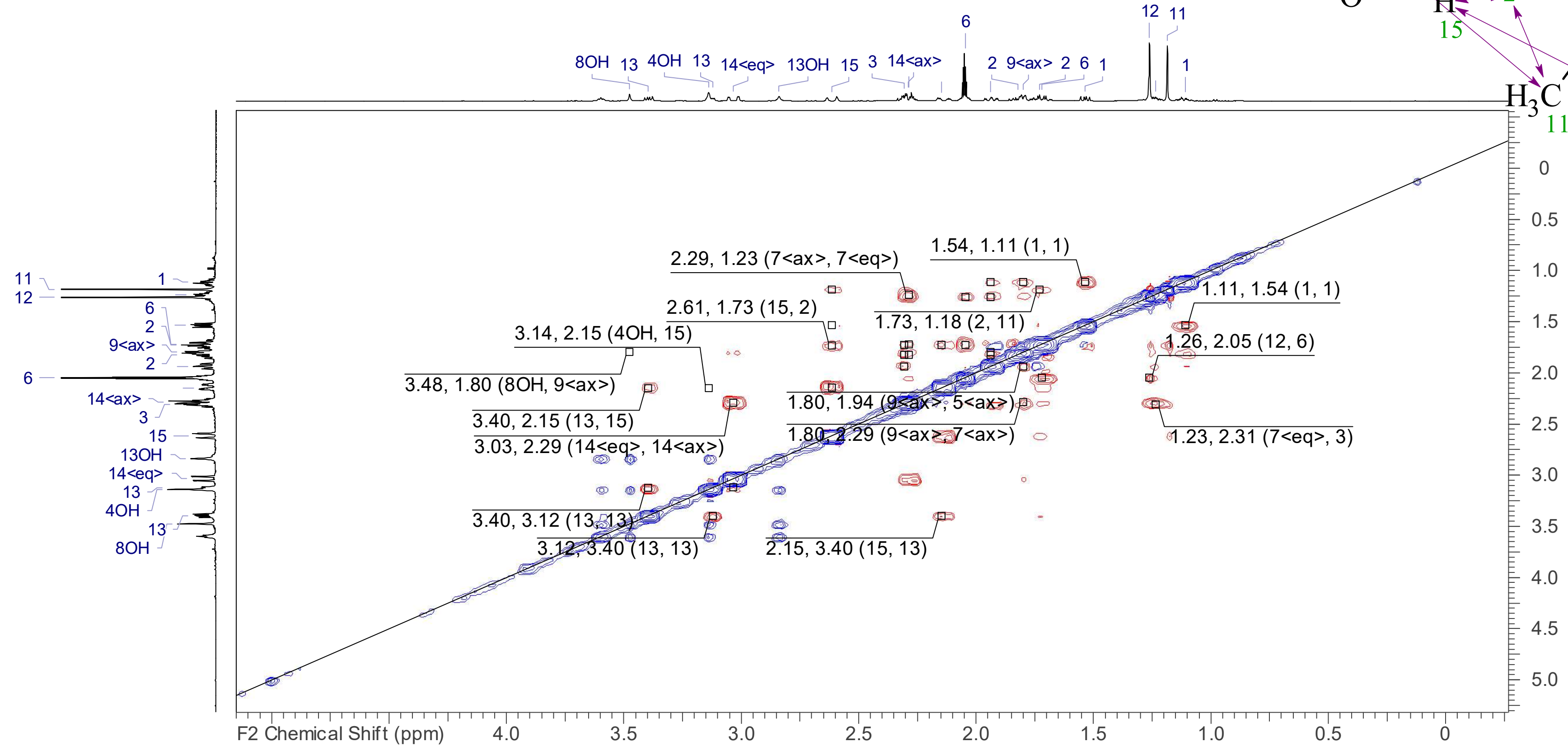

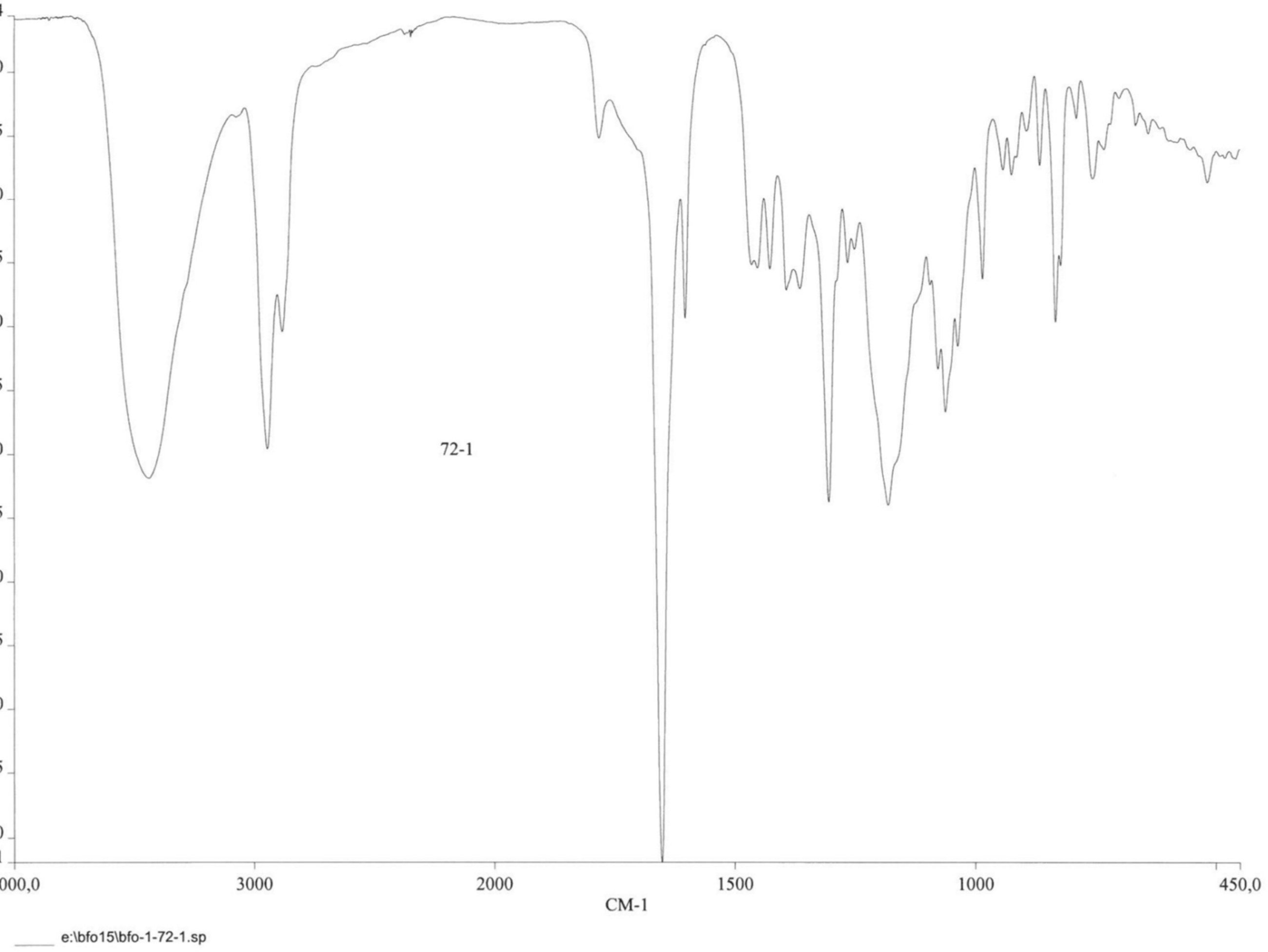

72-1
000,0
3000
2000
1500
1000
450,0
CM-1
e:\bfo15\bfo-1-72-1.sp

Figure S5: IR spectrum (KBr) of 4-Hydroxy-pleurogrisein (11).

Table S2: ^{1}H, ^{13}C NMR data (500 MHz, DMSO-d_6) of 4-Hydroxy-pleurogrisein (11).

Pos	δ_C,	Type	δ_H	(J in Hz)
1	38.3	CH_2	0.99,	m
			1.41,	dd (11.5, 7.1)
2	26.0	CH_2	1.70,	m
			1.58,	m
3	49.5	CH	2.16,	m
4	71.2	C		
5	53.1	CH	1.78,	m
6a	21.9	CH_2	1.56,	m
6b			1.88,	m
7a	38.2	CH_2	1.08,	s
7b			2.14,	m
8	73.6	C		
9	50.1	CH	1.66,	br d (7.5)
10	42.3	C		
11	29.4	CH_3	1.07,	s
12	30.7	CH_3	1.14,	s
13	62.5	CH_2	3.14,	d (10.8)
			2.90,	d (10.8)
14a	21.1	CH_2	2.88,	d (20.1)
14b			2.17,	m
15a	25.9	CH_2	2.07,	br d (21)
15b			2.44,	d (21)
16	141.3	C		
17	141.6	C		
18	187.3	C		
19	136.5*	CH	6.78,	s
20	136.2*	CH	6.78,	s
21	187.0	C		

* inter-changable

¹H NMR (500 MHz, DMSO-d_6) of 4-Hydroxy-pleurogrisein (11).

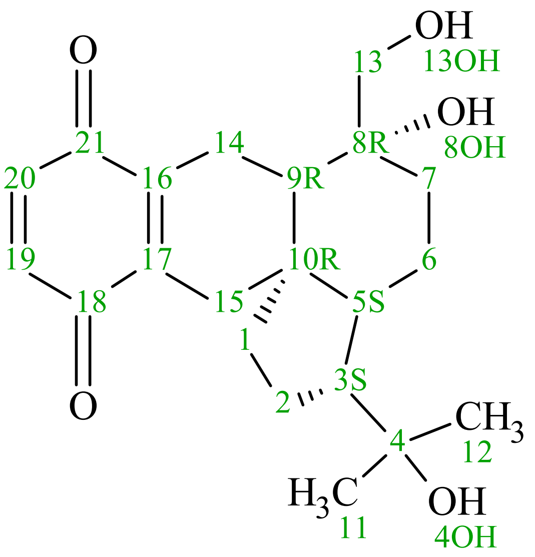

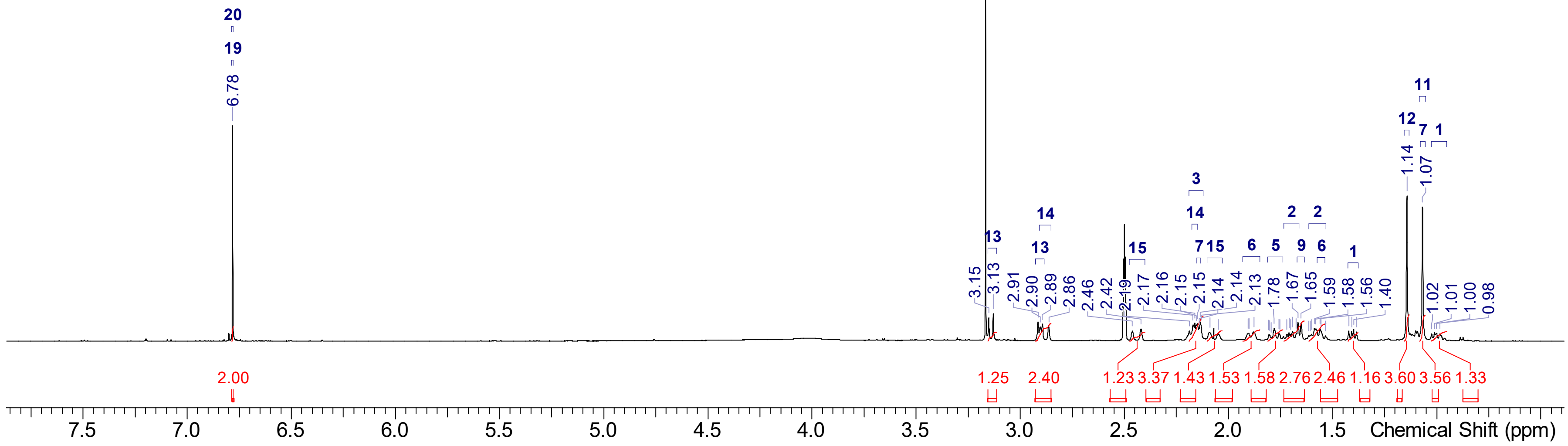

^{13}C NMR (125 MHz, DMSO-d_6) of 4-Hydroxy-pleurogrisein (11).

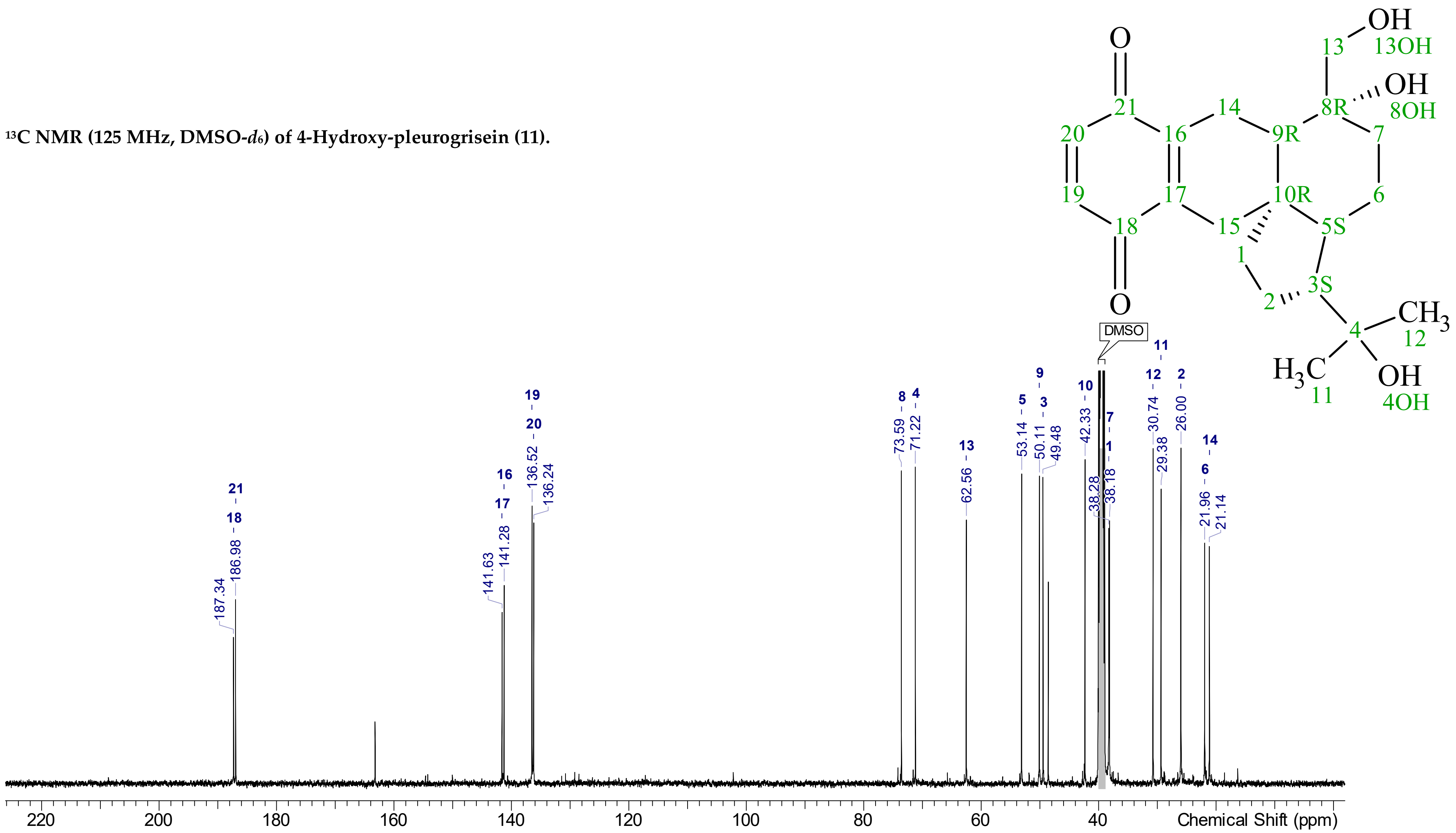

^{1}H, ^{1}H COSY NMR (500 MHz, DMSO-*d*6) of 4-Hydroxy-pleurogrisein (11).

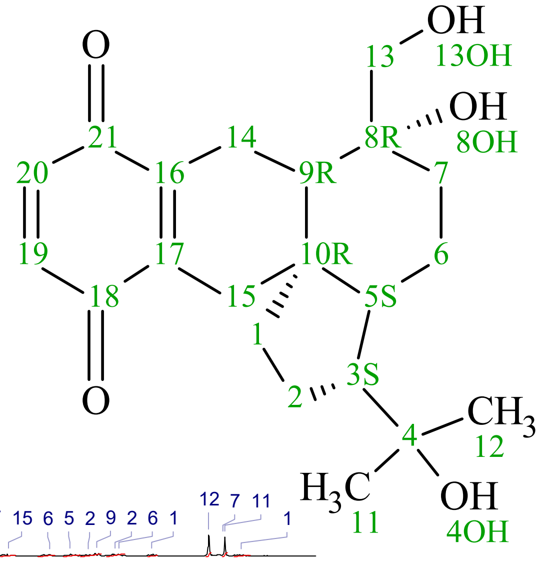

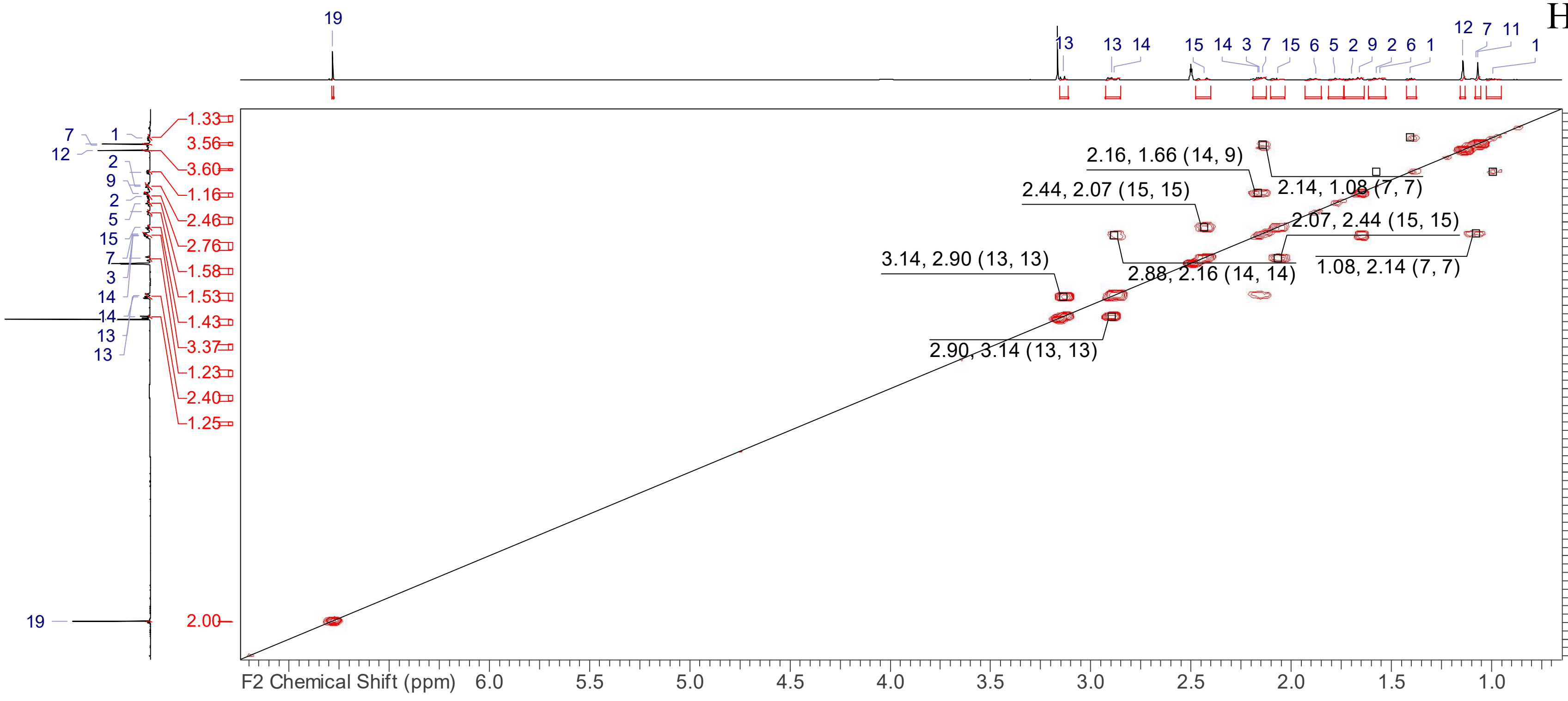

HSQC NMR (500 MHz, DMSO-d_6) of 4-Hydroxy-pleurogrisein (11).

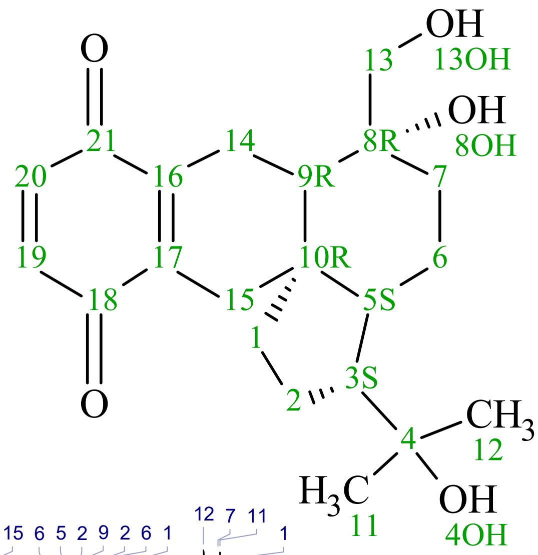

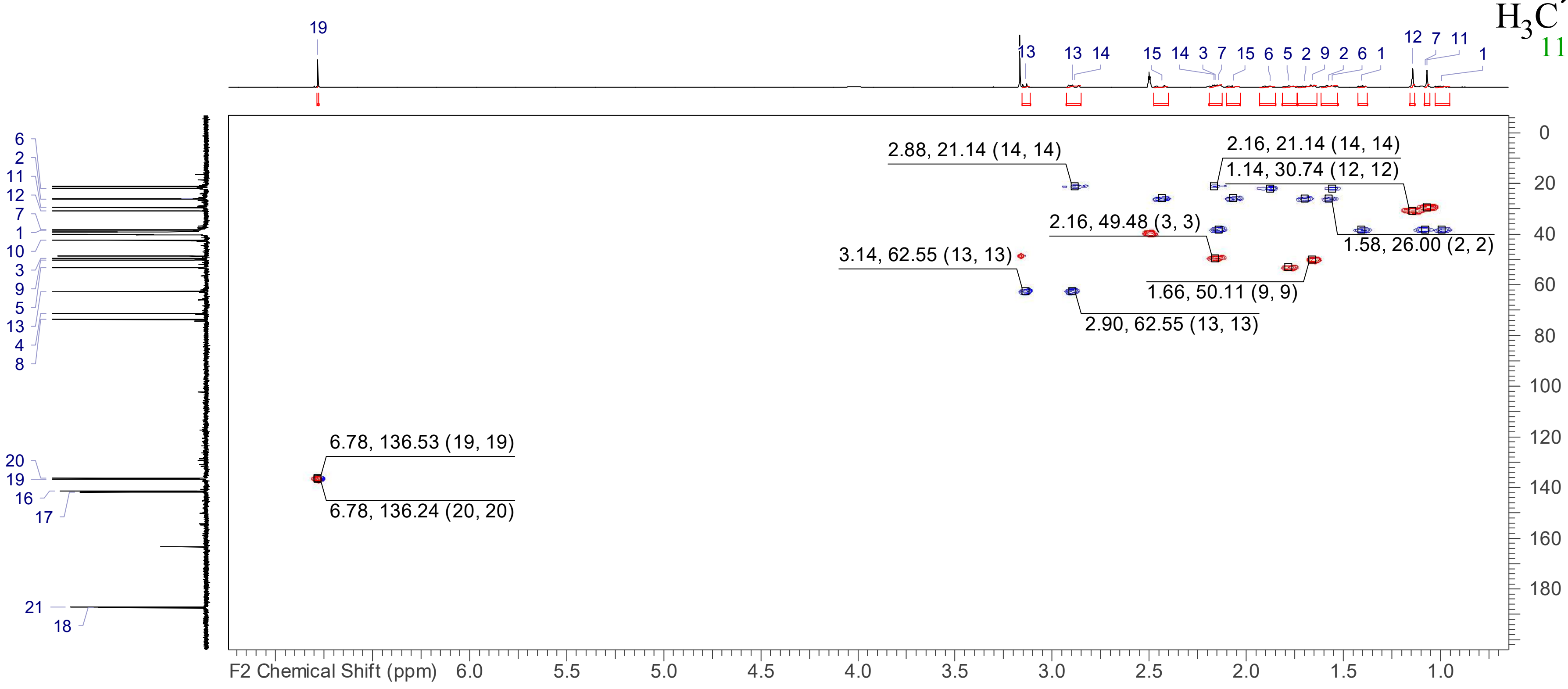

HMBC NMR (500 MHz, DMSO-d_6) of 4-Hydroxy-pleurogrisein (11).

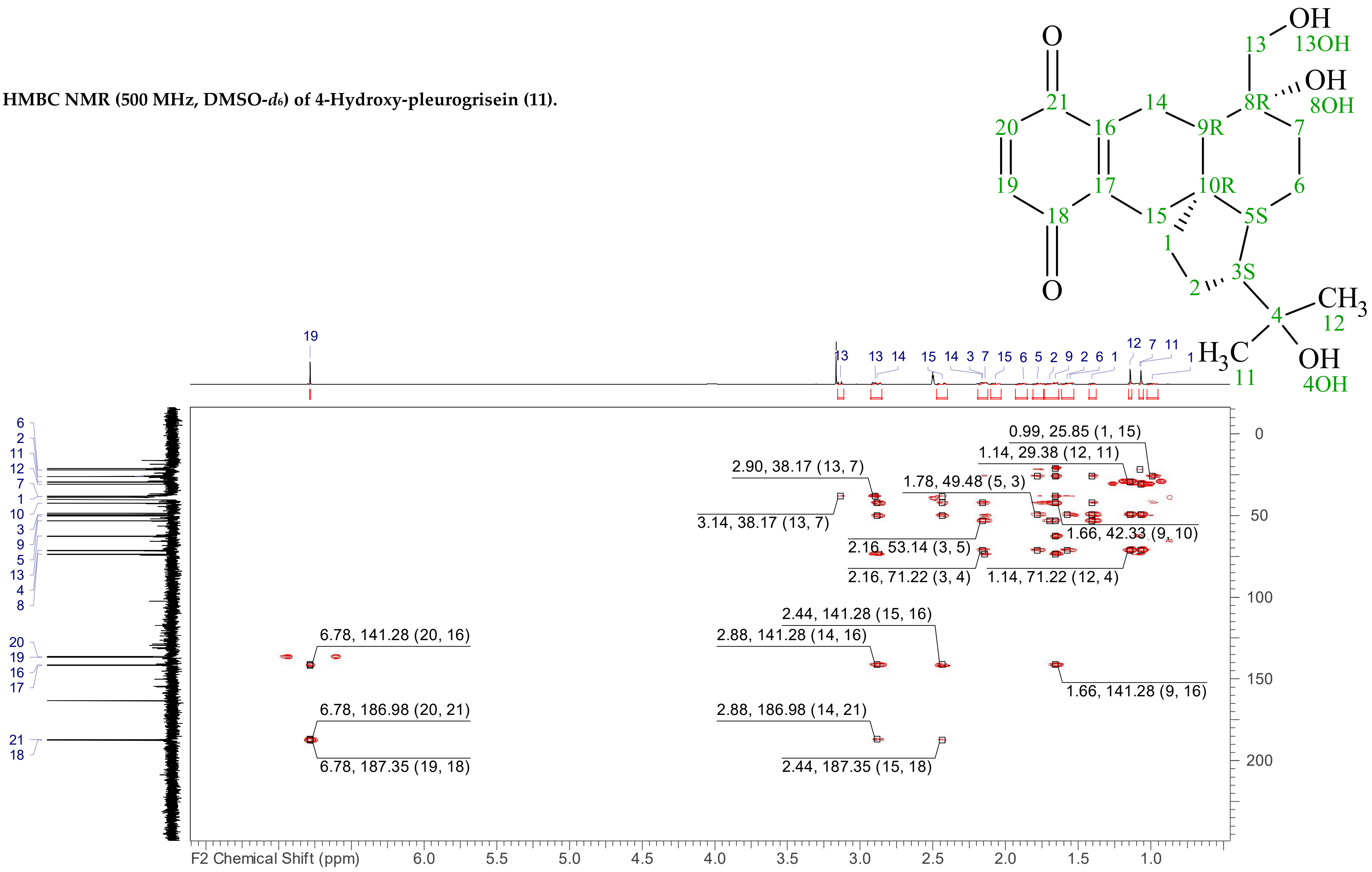

1,1 ADEQUATE NMR (500 MHz, DMSO-d_6) of 4-Hydroxy-pleurogrisein (11).

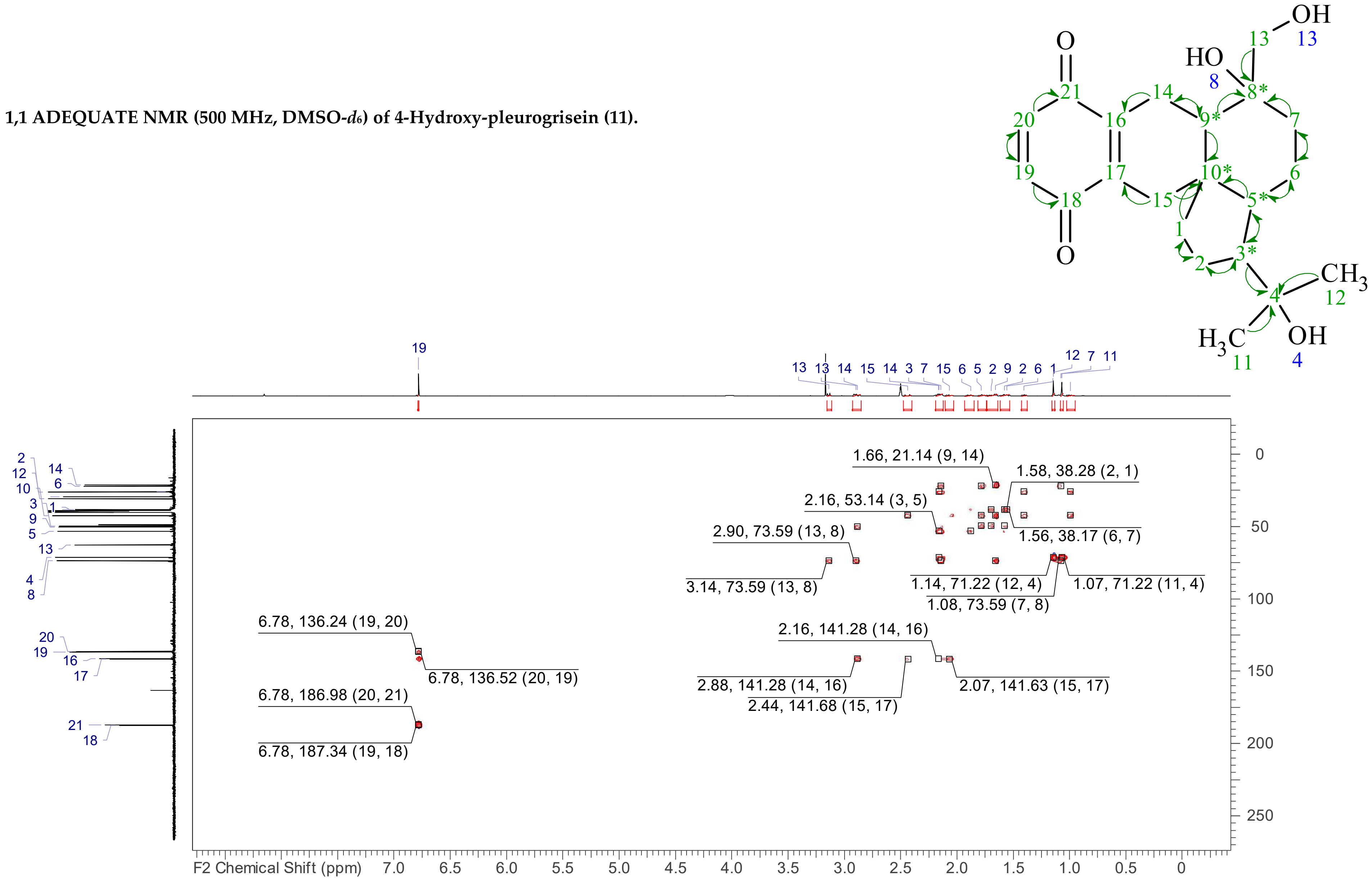

^{1}H, ^{1}H ROESY NMR (500 MHz, DMSO-*d*$_6$) of 4-Hydroxy-pleurogrisein (11).

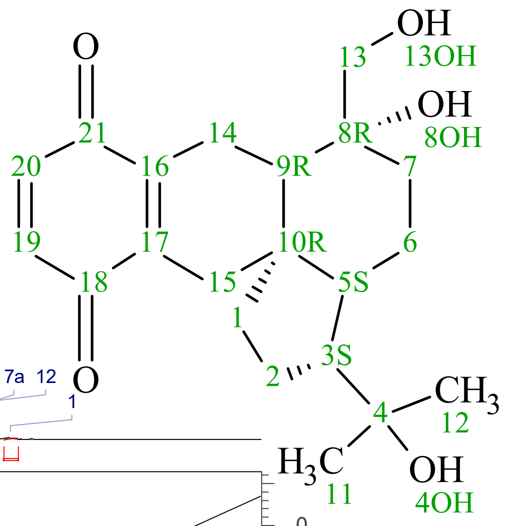

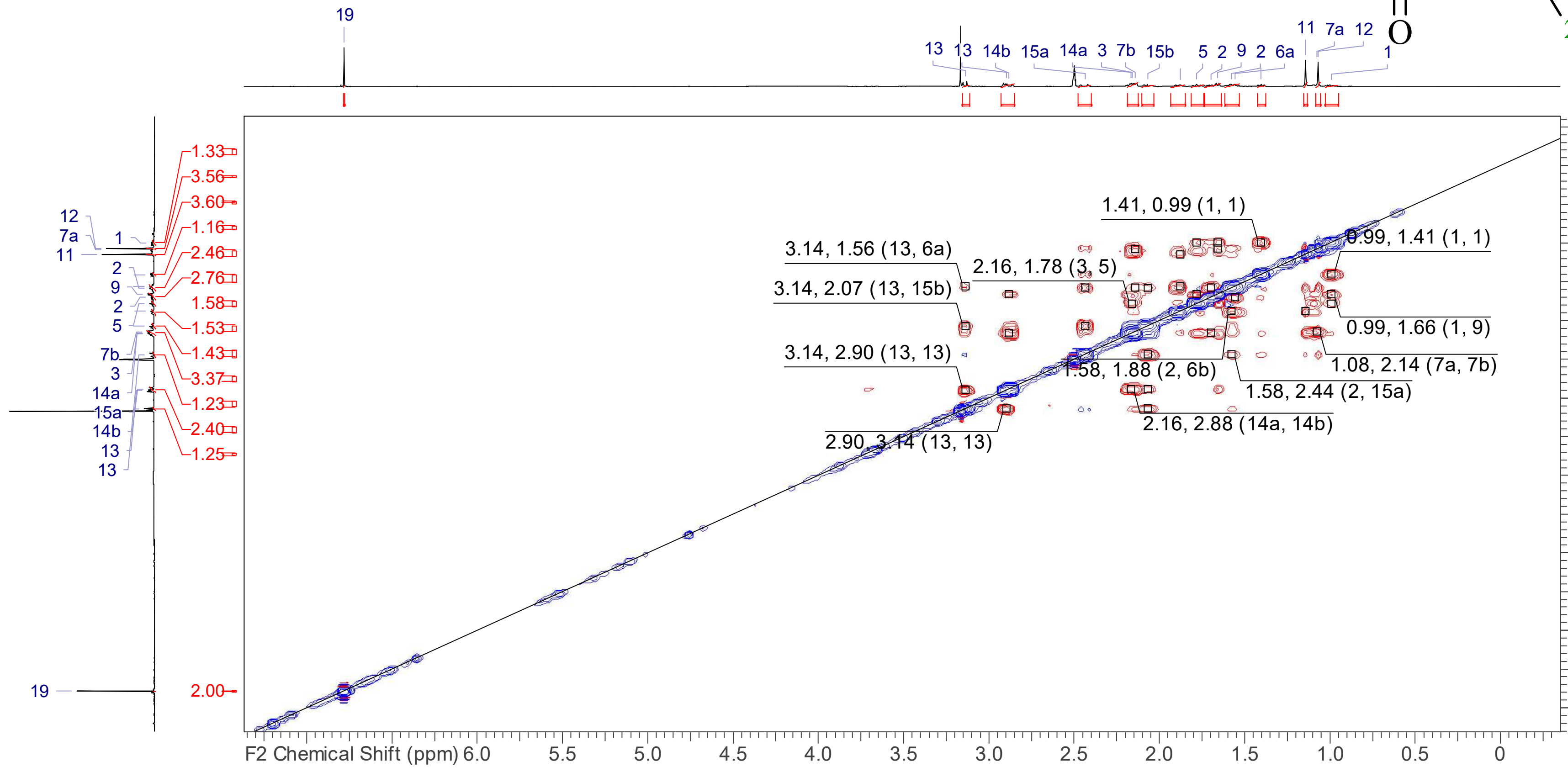

Supplement: Supplementary file 1 [file molecules-23-02697-s001.pdf]
